# Future climate conditions accelerate wheat straw decomposition alongside altered microbial community composition, assembly patterns, and interaction networks

**DOI:** 10.1038/s41396-022-01336-2

**Published:** 2022-11-09

**Authors:** Sara Fareed Mohamed Wahdan, Li Ji, Martin Schädler, Yu-Ting Wu, Chakriya Sansupa, Benjawan Tanunchai, François Buscot, Witoon Purahong

**Affiliations:** 1 Department of Soil Ecology, UFZ-Helmholtz Centre for Environmental Research, Halle (Saale), Germany; 2 Department of Botany & Microbiology, Faculty of Science, Suez Canal University, Ismailia, Egypt; 3 School of Forestry, Central South University of Forestry and Technology, Changsha, PR China; 4 German Centre for Integrative Biodiversity Research (iDiv) Halle-Jena-Leipzig, Leipzig, Germany; 5 Department of Community Ecology, UFZ-Helmholtz Centre for Environmental Research, Halle (Saale), Germany; 6 Department of Forestry, National Pingtung University of Science and Technology, Pingtung, Taiwan; 7 Department of Biomedical Science and Environmental Biology, Kaohsiung Medical University, Kaohsiung, Taiwan, ROC; 8 Department of Biology, Faculty of Science, Chiang Mai University, Chiang Mai, Thailand

**Keywords:** Soil microbiology, Environmental sciences

## Abstract

Although microbial decomposition of plant litter plays a crucial role in nutrient cycling and soil fertility, we know less about likely links of specific microbial traits and decomposition, especially in relation to climate change. We study here wheat straw decomposition under ambient and manipulated conditions simulating a future climate scenario (next 80 years) in agroecosystems, including decay rates, macronutrient dynamics, enzyme activity, and microbial communities. We show that future climate will accelerate straw decay rates only during the early phase of the decomposition process. Additionally, the projected climate change will increase the relative abundance of saprotrophic fungi in decomposing wheat straw. Moreover, the impact of future climate on microbial community assembly and molecular ecological networks of both bacteria and fungi will strongly depend on the decomposition phase. During the early phase of straw decomposition, stochastic processes dominated microbial assembly under ambient climate conditions, whereas deterministic processes highly dominated bacterial and fungal communities under simulated future climate conditions. In the later decomposition phase, similar assembly processes shaped the microbial communities under both climate scenarios. Furthermore, over the early phases of decomposition, simulated future climate enhanced the complexity of microbial interaction networks. We concluded that the impact of future climate on straw decay rate and associated microbial traits like assembly processes and inter-community interactions is restricted to the early phase of decomposition.

## Introduction

Climate change influences ecosystem functions carried out by microorganisms and their metabolic activities in several ways [1, 2]. One of the principal ecosystem functions governed by microorganisms is litter decomposition, which impacts both carbon (C) sequestration and nutrient cycling in terrestrial ecosystems [3]. Bacteria and fungi are major domains responsible for approximately 90% of all organic matter decomposition [4]. Therefore, exploring how climate change influences potential decomposer microorganisms is indispensable for developing accurate projections of soil C stock and altered nutrient dynamics in the future. While most studies have focused on litter decomposition in forests [5–7], less attention has been paid to this ecological process in agricultural lands. In agroecosystems, large quantities of straw are generated as byproducts from several crops, such as wheat and other cereals, and aid in maintaining soil organic matter content and supplying nutrients to subsequent crops [8].

Previous studies have addressed litter decomposition rates based on multiple interacting factors such as climate, substrate traits, quantity, and the presence of decomposer soil microorganisms [9–12]. Elevated temperature and precipitation were found to accelerate decomposition rates through simulating exudation of decomposition-related enzymes [13, 14]; however, chronic increase in temperature causes a rapid decline in enzyme activity and a reduction in litter quality. These two mechanisms combined result in complex and often unexpected responses on decomposition rate [15, 16]. In addition, drought reduces decomposition rates by limiting the metabolic activity of decomposers and reducing litter quality [17]. Therefore, continuous cycles of drying and re-wetting cause variations in decomposition rates and the proportions of mineralized and accumulated nutrients in decomposed residues over time [15]. It is thus uncertain how future climate with its anticipated, increased temperatures and altered precipitation patterns, will modify decomposition rates and the release rates of decomposed compounds to soil.

Microorganisms show remarkable differences in the degree to which they tolerate abiotic conditions [18]. Therefore, environmental filtering, which captures along with niche partitioning how abiotic factors that shape communities, is expected to cause changes in the initial microbial communities colonizing organic matter and lead to distinct successional patterns over time [6]. When microbial community composition changes over time, antagonistic and synergistic interactions among species also change. Consequently, microbial assembly is driven by different temporal deterministic biotic (species interaction) and abiotic factors (environment and nutrient resources) [19]. Conversely, stochastic events, such as dispersal and random drifts play important roles in shaping the microbial community structure [20, 21]. Previous studies have confirmed that both deterministic and stochastic factors interact simultaneously to shape the assembly of microbial communities [22, 23]. For instance, Bao et al. reported that deterministic processes govern bacterial community composition within decomposition stages of paddy straw while ecological drift showed a relative importance across decomposition stages [24]. There has also been environmental evidence that extreme environmental conditions mediate the importance of stochastic community assembly [25, 26]. For instance, Chas demonstrated that under extreme environmental conditions, a large proportion of species might be eliminated by filtering from the local community [26]. Consequently, even if the process of assembly is completely random, the smaller species pool that survives in extreme conditions, lead to a higher site-to-site similarity among communities, and a niche-assembled structure. However, the mechanisms by which climate change alters the interplay between deterministic and stochastic assembly processes of decomposer microorganisms at the temporal scale remains unclear.

Considerable efforts have been made to gauge the effects of climate change on microbial communities and their feedback on the decomposition process. For instance, Treseder et al. found that warming induces shifts in fungal communities, which might be accompanied by an increase in the breakdown of recalcitrant carbon [27]. In addition, a decline in microbial abundance under drought conditions is associated with a decline in litter mass loss [17]. Moreover, a shift in the microbial community composition in response to climate change affects the decomposition rate [9, 28]. Despite preliminary findings indicating the importance of these relationships, the manner in which changes of microbial community composition and functional guilds in response to climate change may influence decomposition on a temporal scale remains understudied.

Climate change was found to alter potential microbial interactions, as investigated by molecular ecological network analysis (MENA). For instance, experimental warming was found to decrease the complexity of prokaryotic networks and simplify interactions among prokaryotes and fungi [29]. Mounting evidence has revealed changes in soil functions as a consequence of changes in cross-trophic interactions under different climates [29–31]. Although, most studies have focused on the ecological interactions of microorganisms in soil, less attention has been paid to microbial interactions in litter microhabitats. Consequently, the possible impact of microbial interactions on the decomposition process is poorly understood. In this regard, it is necessary to consider a combination of all microbial community traits (community composition, diversity, assembly, ecological guilds, activities, and interaction networks) to better understand actual microbial responses to future climate and the significance of their influence on decomposition process [32, 33].

Climate models predict an increase in global temperatures and regional changes in precipitation patterns for the rest of the century [34]. This has inspired the design of the Global Change Experimental Facility (GCEF), located in Central Germany, in the framework of which this work was performed. This facility was designed to explore the consequences of a predicted future climate scenario, projected for the years between 2070 and 2100, on ecosystem functions and processes on large natural field plots in Central Germany [35]. The central goal of this research was to understand whether and how future climate affects the decomposition process in agroecosystems as well as the assembly process, structure, functions, and interactions of associated microorganisms, thus helping to provide a clue for the way of carbon and nutrient turnover and microbial contribution to the decomposition process in the next to 5–8 decades. For this purpose, we performed a litterbag experiment and combined assays of chemical composition and extracellular enzymes of wheat straw with high-throughput sequencing of bacterial 16S rRNA gene and fungal ITS2 to examine the wheat straw decomposition rate and nutrient dynamics, as well as the temporal dynamics of microbial communities decomposing the straw residues. The experiment was performed under field conditions subjected to the future climate scenario. These findings were compared with those under ambient climate regimes. We hypothesized that: (i) future climate conditions will increase the nutrient release rate, enzymes production, and accelerate the decomposition rate of wheat straw; (ii) the microbial community will modulate its response to both environmental factors and available chemical nutrients in straw residues under future climate conditions, providing feedback to the decomposition rate under both climate regimes; (iii) future climate conditions will reduce the relative importance of stochastic processes in shaping microbial assembly; and (iv) future climate conditions will reshape microbial interaction networks in decomposing litter over time.

## Materials and methods

### Study site, and research platform

The study was conducted within the Global Change Experimental Facility (GCEF) at the field research station of the Helmholtz Center for Environmental Research in Bad Lauchstädt, Saxony-Anhalt, Germany (51°23′30″ N, 11°52′49″ E, 116 m above sea level). The GCEF consists of 50 field plots (400 m^2^ each). Half of these plots (25/50) are subjected to ambient climate, and the other half (25/50) are under settings mimicking a future climate regime (Supplementary Fig. S1) [35]. The future climate regime is a consensus scenario across three models [36–38] of climate change which project altered precipitation patterns and increased temperature in Central Germany in the next 50–70 years. To manipulate both precipitation and temperature, future climate plots (Supplementary Fig. S1) are equipped with mobile shelters and side panels, as well as an irrigation system and a rain sensor. Continuous adjustment of irrigation or roof closing reduced precipitation by ~20% in summer and increased it by ~10% in spring and autumn. To simulate increased temperature, the standard method “passive nighttime warming” was applied by closing the shelters and panels from sundown to sunrise to increase the mean daily and night temperatures by ~0.55 °C and 2°–3 °C, respectively. More details regarding the GCEF design are published elsewhere [35]. The effects of climate manipulation on total precipitation and soil temperature during the study period were shown in Supplementary Fig. S2. The soil of the study field is a highly fertile Haplic Chernozem, characterized by high water-holding capacity and humus content down to a depth of 40 cm [39]. Basal soil physicochemical properties were similar for experimental plots under ambient and future climate regimes (C/N ratio = ∼10–14; pH = 7.16 ± 0.02 (mean ± SE)).

### Wheat straw decomposition experiment

We set up a litterbag experiment on the conventionally managed agricultural plots subjected to both ambient (5 plots) and future climate scenarios (5 plots). The conventional farming practice is characterized by a regional crop rotation consisting of winter wheat, winter barley and winter rape with application of fungicides, pesticides, additives and mineral fertilizer (Supplementary Table S1). Details of the experimental design are described elsewhere [28]. Briefly, wheat straw (10 cm aboveground) was collected from GCEF plots after harvest of winter wheat (*Triticum aestivum* L., variety RGT Reform), and allowed to oven-dry at 25 °C for three days. Litterbags (20 cm × 15 cm, 5 mm mesh size) containing ∼10 g of oven-dried plot-specific straw were returned to the field plots in mid-August (Supplementary Fig. S3). Litterbags were retrieved on six sampling times; in 2018 at the onset of the experiment (0 days; 0 D), on 14 September (30 days after incubation; 30 D), 15 October (60 days after incubation; 60 D), 13 December (120 days after incubation; 120 D), and in 2019 on 12 April (240 days after incubation; 240 D) and 09 October (420 days after incubation; 420 D) (Supplementary Fig. S3). We further refer to the first three sampling times as “Early decomposition phase”, and the later sampling times as “Later decomposition phase”. At each sampling time, one litterbag was taken from each plot and transported on ice to the laboratory. In total, 60 litterbags were collected from both ambient and climate plots (2 climate regimes × 6 sampling times × 5 replicate plots). Wheat straw from ambient and future climate plots were characterized separately.

### Decomposition rate and chemical analysis of wheat straw

The dry mass of wheat straw samples was determined after 24 h of oven drying at 105 °C (Supplementary Table S2). Remaining straw mass *X*_*t*_ was calculated as the difference between initial straw mass *X*_*0*_ and straw mass loss at time *t* [40]. The decomposition rate constant (*k*) was calculated using the exponential decay model of Olson [41] according to the equation:

$$k \,=\, - \ln \left( {X_t/X_0} \right)/t,$$

Wheat straw quality was initially determined in undecomposed straw (0 D) and again at the five sampling events (Supplementary Table S2). Total carbon (TC) and total nitrogen (TN) concentrations in ground straw were determined by dry combustion at 1000 °C with a CHNS-Elemental Analyzer (Elementar Analysensysteme GmbH, Hanau, Germany) as explained by the manufacturer. Available phosphorus was extracted and measured according to Bray 1 method [42]. Cations (K^+^, Mg^2+^, and Ca^2+^) were determined by atomic absorption spectrophotometry according to the manufacturers’ specifications (Hitachi Z 5300, Hitachi—Science & Technology, Japan). The wheat straw pH was measured using WTW Multi 3510 IDS (Weilheim, Germany). The release rate of nutrients during straw decomposition in each treatment was calculated as follows: Release rate (%) = (C_0_ – C_t_)/C_0_ × 100%, where C_0_ and C_t_ are concentrations of each chemical component before and after decomposition, respectively, for each sampling time *t*. Positive and negative results mean net mineralization and accumulation, respectively.

### Decomposition-related microbial function

Aliquots of all wheat straw samples from 0 to 420 days were used to determine potential activity of microbial extracellular hydrolytic (β-glucosidase (EC 3.2.1.21), N-acetyl-glucosaminidase (EC 3.2.1.50), acid phosphatase (EC 3.1.3.2)), and oxidative (phenol oxidase (EC 1.10.3.1), and peroxidase (EC 1.11.1.7)) enzymes related to straw decomposition. The activity of β-glucosidase, *N*-acetyl-glucosaminidase, and acid phosphatase were measured spectrophotometrically with methylumbelliferone-linked substrates at room temperature with sodium acetate buffer [43, 44]. Ligninolytic activities of phenol oxidase, and peroxidase were analyzed with 3,3′,5,5′-tetramethyl-benzidine (TMB) as described previously [45]. Fluorescence (excitation wavelength 360 nm, emission wavelength 460 nm) or absorbance (wavelength 450 nm) were measured with a 96-well plate reader (Synergy HT, Biotek). Enzyme activity was then calculated as nmol activity h^−1^ g dry straw^−1^ using blanks, controls, and standards present on each plate (Supplementary Table S3).

### Microbial community analyses

We investigated straw-inhabiting bacterial and fungal communities with MiSeq sequencing targeting the 16S rRNA and ITS2 gene amplicons, respectively. Genomic DNA was extracted using a DNeasy PowerSoil kit (Qiagen, Valencia, CA, USA) following the manufacturer’s instructions. The 16S rRNA gene V5-V7 fragments were amplified using the primer pairs, BAC799F [46] and BAC1193R [47], while the fungal ITS2 region was amplified using fITS7 and ITS4 [48] primer pairs. The PCR products were purified using Agencourt AMPure XP beads (Beckman Coulter Inc., Indianapolis, IN, USA). Indexing of the purified amplicons was done using the Nextera index kit (Illumina, San Diego, CA, USA). Finally, the amplicon libraries were quantified by PicoGreen assays (Molecular Probes, Eugene, OR, United States) and pooled to give an equimolar representation of each. MiSeq sequencing (Illumina) was performed at the Department of Soil Ecology, UFZ-Helmholtz Center for Environmental Research in Halle (Saale), Germany.

The bioinformatics analysis of the raw sequences was carried out using the DADA2 package [49] via the pipeline dadasnake [50]. Briefly, sequences corresponding to the forward and reverse primers were trimmed from the demultiplexed raw reads using cutadapt [51]. Bacterial reads were filtered and trimmed using the parameters: quality score 9, and maximum expected error 2, while the ITS sequences were filtered and trimmed with the parameters: quality score 9, and maximum error 5. Merging was carried out with 2 mismatch and a minimum overlap of 20 nucleotides for both bacterial and fungal sequences. After chimera removal, we obtained 796,755 high-quality bacterial reads clustered into 3341 amplicon sequence variants (ASVs), and 1,763,814 high-quality fungal reads clustered into 551 ASVs. For normalization, the datasets were rarefied to the minimum number of reads per sample (Bacteria: 2829 reads represented by 2923 ASVs; Fungi: 8373 reads represented by 522 ASVs). The taxonomic identification of each ASV was performed by aligning it against the SILVA database v138 [52] for prokaryote 16S rRNA gene and the UNITE v7.2 (01.12.2017) database [53] for fungal ITS. The Tax4Fun [54] R package, which employs 16S rRNA gene-based taxonomic information, and the Kyoto Encyclopedia of Genes and Genomes (KEGG) database were used to predict the metabolic functional attributes of bacterial communities. We focused on predicted genes involved in litter decomposition process. Fungal ecological functions were predicted using the database, FungalTraits [55]. Similarly, we focused on the dynamics of the major ecophysiological functions that potentially related to litter decomposition; plant pathogens, litter, wood, and general saprotrophs.

### Statistical analyses

All statistical analyses were carried out in SPSS software and with the vegan package [56] in R-4.1.0 [57]. To test the influence of climate regime, sampling time, and their interaction on wheat straw mass loss, the dynamics of nutrients release, as well as extracellular enzymes production, we applied repeated-measured analysis of variance (ANOVA). Climate was used as the “between‐subject” factor while time was used as the “within-subject” factor. The Bonferroni method was used to adjust *p* values across pairwise tests. Data normality was checked by Jarque–Bera test [58].

To check the significant differences between microbial communities across the experimental factors (climate regime, and sampling time) we used two-way PERMANOVA [59] based on Bray–Curtis distances. To visualize the variation in microbial communities, principal coordinate analysis (PCoA) was performed. Significant (*p* < 0.05) soil and straw variables were fitted to the PCoA ordination plots using the Goodness-of-fit statistics (*R*^2^). To analyze the impact of climate regime, sampling time, and their interaction on microbial diversity, repeated-measured analysis of variance (ANOVA) was used. To investigate effects of climate and time on the most abundant bacterial and fungal orders and genera, repeated-measured analysis of variance (ANOVA) was used for normally distributed data. On the other hand, Friedman test and Mann–Whitney-U-Test were applied for non-normally distributed data. To explore the impact of climate on microbial phylogeny, maximum likelihood phylogenetic trees were generated for the top 100 frequently detected ASVs using MEGA-X and displayed with the Interactive Tree of Life (iTOL v6, http://itol.embl.de).

### Microbial assembly processes

To discern how climate change could affect assembly patterns of wheat straw microbiomes, we quantified the relative contribution of deterministic and stochastic processes in microbial community assembly. The phylogenetic normalized stochasticity ratio (pNST), an index quantifying average stochasticity within a group of samples based on phylogenetic beta diversity, was carried out based on the null model theory in Stegen et al. [60] and Ning et al. [61]. 50% indicated the boundary point between more deterministic (pNST < 50%) and more stochastic (pNST > 50%) assembly. In addition to pNST, the beta nearest taxon indices (βNTI) and Raup-Crick (RC_bray_) null model based on Bray–Curtis dissimilarity were further used to quantify dispersal-based stochastic ecological processes [62]. According to the information described by Stegen et al. [62], the homogeneous and variable selection are indicated by βNTI < −2 and βNTI > +2, respectively. The relative importance of dispersal limitation and homogenizing dispersal processes were quantified by |βNTI| < 2 but RC_bray_ > +0.95 and RC_bray_ < −0.95, respectively, and the undominated process was estimated by |βNTI| < 2 and |RC_bray_| < 0.95. Both indices above mentioned were calculated by the ‘iCAMP’ R package (https://github.com/DaliangNing/iCAMP1).

### Construction of molecular ecological networks (MENs)

To investigate how future climate regime could impact interactions of microorganisms inhabiting wheat straw, we calculated MENs for both bacteria and fungi. All MENs were constructed and the topological features were analyzed in the online pipeline MENAP [63, 64] using Pearson’s correlation (*r* = 0.7, *p* = 0.01) of non-log transformed ASVs. We constructed four time-series networks; (i) ambient (0–60 D), (ii) future (0–60 D), (iii) ambient (120–420 D), and (iv) future (120–420 D) from ASVs which occurred in 75% of samples in each of the four groups. Based on detected modules, among-module connectivity and inter-module, connectivity were calculated, and nodes were assigned to one of four possible network roles: network hubs, module hubs, connectors or peripherals [63]. To discern the relationships between network structure and wheat straw properties, environmental factors, as well as decomposition-related microbial function (enzymes activity) and ecosystem function (straw decomposition), partial Mantel tests were performed between the distance matrices of the ASV connectivity. Networks and modules were visualized in Gephi 0.9.2.

## Results

### Decomposition rate and chemical analysis of wheat straw

**Fig. 1:**
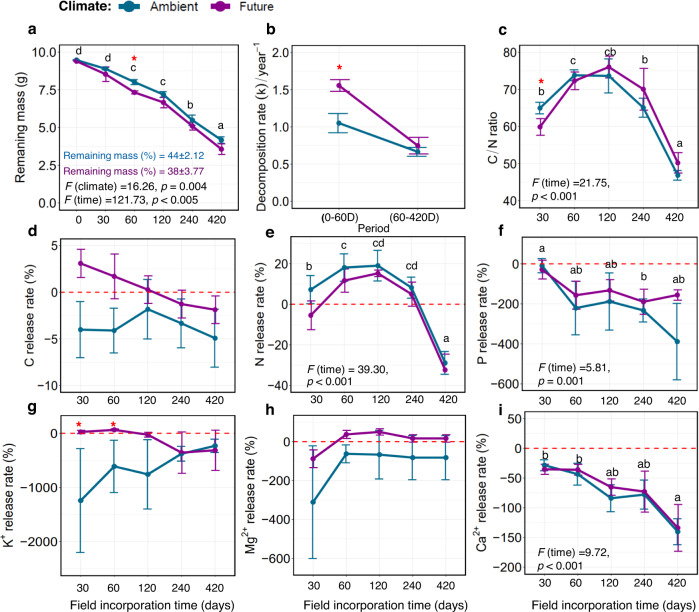
Decomposition rate and chemical analysis of wheat straw. **a** Mass loss and **b** decomposition rates (*k*) of wheat straw under ambient and future climate regimes. **c**–**i** Chemical analysis and dynamics of nutrients content of wheat straw over 420 days of decomposition. Values in a given graph (means ± SE, *n* = 5) labeled with different letters differ significantly (repeated-measured ANOVA, followed by Bonferroni, *p* < 0.05). Asterisks show significantly different values under climate regimes.

After 420 days of field incorporation, only ~44% (±2.12%) and ~38% (±3.77%) of the wheat straw mass remained in the ambient and future climate plots, respectively (Fig. 1a). Under future climate conditions, the loss of wheat straw dry mass significantly accelerated over time (*F*_(climate)_ = 16.26; *F*_(time)_ = 121.73, *p* < 0.005; Fig. 1a). Such a decrease in straw mass is a result of the significantly higher decomposition rate (*k*) in the early phase (0–60 D) of decomposition under future climate regimes (Fig. 1b). The C/N ratio of the straw was 58% (ambient climate) and 64% (future climate), which significantly increased during 60–240 D, then reduced to 47% and 50%, respectively, at the end of our experiment (*F*_(time)_ = 21.75, *p* < 0.001; Fig. 1c). Analysis of the chemical properties of straw revealed a net immobilization of all measured elements under both climate regimes (Fig. 1d–i; Supplementary Table S4). Whereas N was released in the early phase (0–60 D), it was highly accumulated at 420 D (Fig. 1e). Similarly, K^+^ was mineralized in the early phase (0–30 D) under the future climate regime, followed by accumulated in the later phases of decomposition (Fig. 1g). Our results indicate that the impact of future climate on decomposition and nutrient dynamics is restricted to the early phase.

### Decomposition-related microbial function

We found that enzyme patterns in wheat straw were not influenced by the future climate regime settings; however, it showed a dynamic pattern over 420 days (hydrolytic enzymes: *F*_(time)_ = 17.95, *p* < 0.001; oxidative enzymes: *F*_(time)_ = 12.93, *p* < 0.001). Generally, the later phase (120–420 D) of wheat straw decomposition showed higher activity of both hydrolytic and oxidative enzymes (Supplementary Fig. S4).

### Microbial diversity changes over time and contributes to straw mass loss

**Fig. 2:**
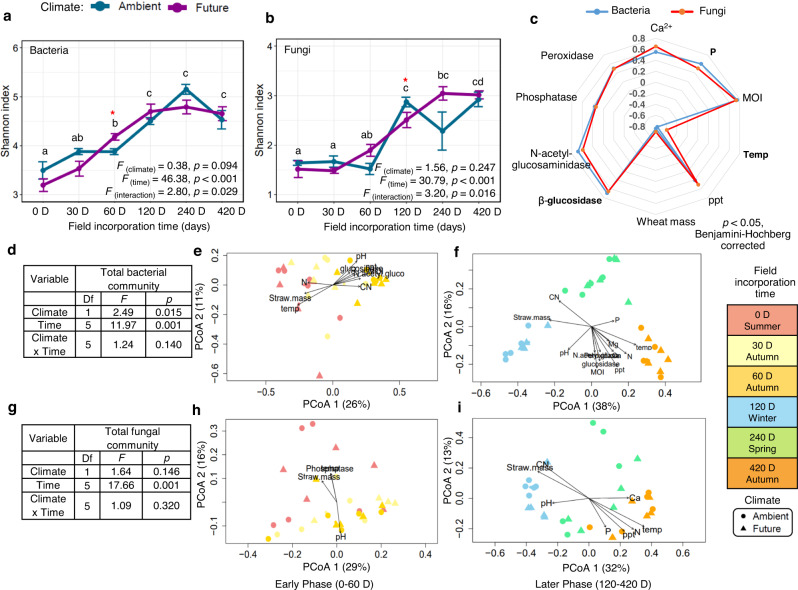
Microbial diversity on decomposing wheat straw over time. **a** Bacteria and (**b**) fungal Shannon’s diversity under ambient and future climate regimes. **c** Radar chart showing factors significantly correlated with bacterial and fungal diversity (Spearman’s rank correlation, *p* < 0.05, Benjamini-Hochberg corrected). Impacts of climate regime, decomposition time, and their interaction on (**d**) bacterial and (**g**) fungal community compositions. Principal Coordinate Analysis (PCoA) dissimilarity matrix (Bray–Curtis dissimilarity matrix, permutations = 999) of the bacteria at (**e**) early and (**f**) later phase and fungi at (**h**) early and (**i**) later phase of decomposition. Vector fitting of the edaphic and wheat variables of plots subjected to ambient and future climate regimes. Significant variables were plotted in black, MOI straw moisture, temp soil temperature at 3 cm depth, ppt precipitation (mm).

The microbial Shannon diversity increased over time on decomposing straw until the end of the experiment (Fig. 2a, b). Conversely, neither bacterial nor fungal diversity differed significantly between the two climatic regimes, except for specific sampling times. Microbial diversity correlated positively with straw nutrients (P and Ca^2+^) as well as moisture content of wheat straw, and precipitation (bacteria: rho = 0.41–0.75, *p* < 0.001; fungi: rho = 0.50–0.73, *p* < 0.001; Fig 2c), whereas it was negatively correlated with temperature (bacterial: rho = −0.77, *p* < 0.001; fungi: rho = −0.59, *p* < 0.001). A clear gradual succession of bacterial and fungal communities on the wheat straw was detected at the ASV level over time (bacteria: *F*_(time)_ = 11.97, *p* = 0.001; fungi: *F*_(time)_ =  17.66, *p* = 0.001; Fig. 2d, g). Interestingly, the future climate regime significantly altered only the bacterial community (*F*_(climate)_ = 2.49, *p* = 0.015). Microbial community compositions were significantly shaped by different straw nutrients and environmental variables during the early and later phases of decomposition (bacteria: Fig. 2e, f; fungi: Fig. 2h, i; Supplementary Table S5). Additionally, variation in the bacterial community was explained more by temperature (early phase: *R*^2^ = 0.75; later phase: *R*^2^ = 0.95, *p* < 0.001) than by precipitation (early phase: *R*^2^ = 0.59; later phase: *R*^2^ = 0.92, *p* = 0.001). Similarly, temperature (early phase: *R*^2^ = 0.25, *p* = 0.022; later phase: *R*^2^ = 0.85, *p* = 0.001) explained higher variation in the fungal community than precipitation (later phase: *R*^2^ = 0.53, *p* = 0.001) that influenced fungi only in the later phase of decomposition (Supplementary Table S3). Finally, our analysis revealed that microbial Shannon diversity correlated negatively with straw mass (bacterial: rho = −0.76; fungi: rho = −0.70, *p* < 0.001; Fig. 2c). Moreover, straw mass significantly associated with microbial community composition with more impact of bacterial (early phase: *R*^2^ = 0.40, *p* = 0.002; later phase: *R*^2^ = 0.75, *p* = 0.001) than fungal (early phase: *R*^2^ = 0.20, *p* = 0.047; later phase: *R*^2^ = 0.75, *p* = 0.001) communities (Supplementary Table S5).

### Shifts in and driving factors of bacterial taxonomy and functional composition over decomposition time and response to future climate regime

**Fig. 3:**
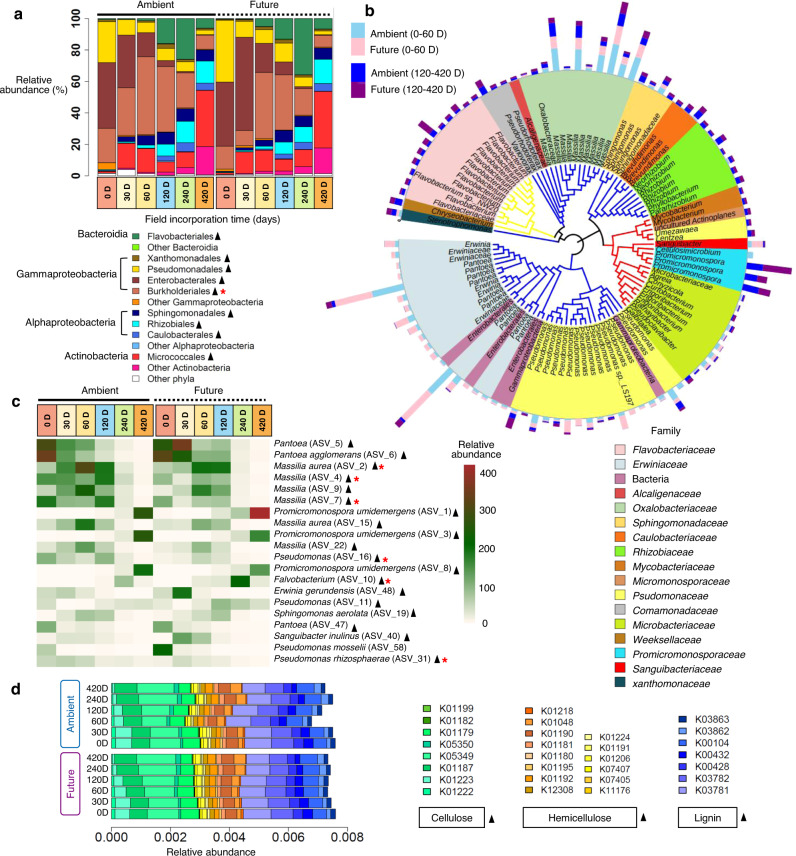
The composition of bacterial communities colonizing wheat straw over 420 days under ambient and future climate regimes. **a** Order level taxonomy (>1% relative abundance). **b** Phylogenetic tree showing the top 100 amplicon sequences variants (ASVs) with highest relative abundance. The taxonomic affiliation at the family level of different Phyla of each ASV is identified by the colors range in the below panel and within the tree. The clade colors showing taxonomy classification at the phylum level; blue: *Proteobacteria*, red: *Actinobacteriota*, yellow: *Bacteroidota*. Microbial abundance at the early (0–60 D) and later (12–420 D) stages of decomposition is indicated in the outer stalked bars. **c** Heat map showing the dynamic of the top 20 ASVs assigned to genus level under both climate regimes. **d** The relative abundance of potential metabolic functional genes of Kyoto Encyclopedia of Genes and Genomes (KEGG) orthologs (KOs) assigned to KEGG pathways involved in wheat straw decomposition. ▲ significant shift of the relative abundance of bacterial taxa or predicted metabolic function over time; * significant effect of climate regime on the relative abundance of bacterial taxa or predicted function.

For the first four successional stages (0–120 D), most bacterial ASVs were assigned to *Gammaproteobacteria* (relative abundance ~96-67% at 0–120 D). The bacterial community changed at 240 D to be dominated by *Bacteroidia* (relative abundance ~31%), followed by *Gammaproteobacteria* (relative abundance ~28%), and *Alphaproteobacteria* (relative abundance ~24%). Finally, at 420 D, *Actinobacteria* (relative abundance ~53%) was the dominant phylum (Fig. 3a). These four predominant phyla were represented by nine highly abundant orders (>1% of all sequences), and all consistently changed during succession in terms of relative abundance. Only the relative abundance of *Burkholderiales* decreased significantly under the future climate regime (*F* = 9.62, *p* = 0.015; Supplementary Table S6). The relative abundances of these orders were associated with soil and straw chemical content variables (e.g., Ca^2+^, P, and moisture content; Supplementary Fig. S5). In contrast to other orders, *Enterobacterales* and *Pseudomonadales* were positively correlated with temperature and negatively correlated with precipitation. We also noticed that the highest ASVs, in relative sequence abundance, were broadly distributed across the phylogenetic tree and showed a strong shift over time (Fig. 3b). Based on the cumulative relative abundances of the top 20 detected ASVs in wheat straw, we found that the bacterial communities were dominated by the genera *Pantoea*, *Massilia*, *Promicromonospora*, *Pseudomonas*, *Flavobacterium*, *Sphingomonas*, and *Sanguibacter* (Fig. 3c). Almost all of these genera exhibited a gradual shift over decomposition time, while some were significantly influenced by the climate regime (Fig. 3c; Supplementary Table S7). Finally, the potential metabolic functional profiles of bacterial communities were predicted based on the 16S rRNA genes of the retrieved bacterial taxa using Tax4Fun according to the KEGG Ortholog groups (KOs). We focused on the predicted genes involved in wheat straw decomposition (cellulose, hemicellulose, and lignin degradation) (Fig. 3d; Supplementary Table S8). Our analysis revealed that the relative abundance of the predicted genes changed over 420 days of field incubation (*p* < 0.001–0.001, Friedman test) but was not influenced by the climate regime (*p* = 0.153–0.717, Mann–Whitney *U* test) (Supplementary Table S9).

### Shifts in and driving factors of fungal taxonomy and functional composition over decomposition time and response to future climate regime

**Fig. 4:**
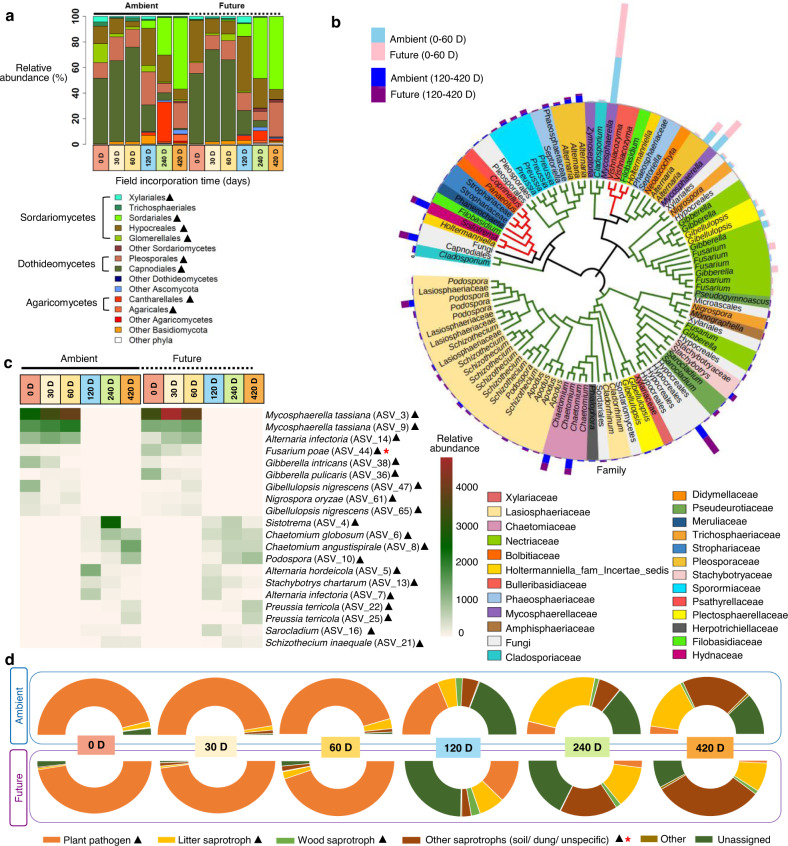
The composition of fungal communities colonizing wheat straw over 420 days under ambient and future climate regimes. **a** Order level taxonomy (>1% relative abundance). **b** Phylogenetic tree showing the top 100 amplicon sequences variants (ASVs) with the highest cumulative relative abundance. The taxonomic affiliation at the family level of different Phyla of each ASV is identified by the colors range in the below panel and within the tree. The clade colors showing taxonomy classification at the phylum level; green: Ascomycota, red: Basidiomycota. Microbial abundance at the early (0D–60D) and later (120D–420D) stages of decomposition are indicated in the outer stalked bars. **c** Heat map showing the dynamic of the top 20 ASVs assigned to genus level under both climate regimes. **d** The relative abundance of predicted ecological fungal traits involved in wheat straw decomposition. ▲ significant shift of the relative abundance of fungal taxa or potential ecological function over time, * significant effect of climate regime on the relative abundance of fungal taxa or predicted ecological function.

The dominant fungal classes during the first three sampling times were Dothideomycetes (relative abundance ~63, 83, 83% at 0 D, 30 D, and 60 D, respectively), followed by Sordariomycetes (relative abundance ~36, 15, 14% at 0 D, 30 D, and 60 D, respectively). This pattern was inversed starting from 120 D sampling time to the end of the experiment. We also detected the increase of Agaricomycetes at 120 D (relative abundance ~3%), 240 D (~21%), and 420 D (~5%) in terms of relative abundance (Fig. 4a). These three predominant classes were represented by nine highly abundant orders (>1% of all sequences), and almost all of them (relative abundance) consistently changed during succession, but were not influenced by climate change (Supplementary Table S10). The first three sampling times were dominated by Capnodiales (relative abundance ~53, 68, 69% at 0 D, 30 D, and 60 D, respectively), replaced by Hypocreales at 120 D (~36%) and 240 D (~22%), and finally by Sordariales at 420 D (~65%). The Basidiomycetous orders, Agaricales and Cantharellales were increased at 240 D (~21%) as they were correlated positively with moisture and precipitation, and negatively with temperature (Supplementary Fig. S6). It was also apparent that dominant straw-inhabiting fungi at the 0–60 D were phylogenetically distinct from those at 120–420 D (Fig. 4b). In addition, dominant fungal genera at 0–60 D were totally replaced by new genera at 120–420 D (Fig. 4c). Except for *Fusarium poae*, none of these genera were influenced by the climate regime (Supplementary Table S11). Investigating fungal ecophysiological functions revealed that the first three sampling times (0–60 D) were dominated by potential plant pathogenic fungi (relative abundance ~90-95%) that declined over time to be replaced by litter, wood and general saprotrophs at 240 D (relative abundance ~62%) and 420 D (relative abundance ~76%) (Fig. 4d; Supplementary Table S12). Interestingly, we found that future climate regime increased the relative abundance of general saprotrophs (soil, dung, unspecific) in decomposing wheat straw. Except for plant pathogen, all functional traits correlated positively to β-glucosidase, N-acetyl-glucosaminidase, acid phosphatase, and peroxidase activity and correlated negatively to straw mass (*r* = −0.63: −0.87, *p* < 0.001) (Supplementary Table S13).

### Impact of future climate and time on assembly processes of straw-inhabiting microorganisms

**Fig. 5:**
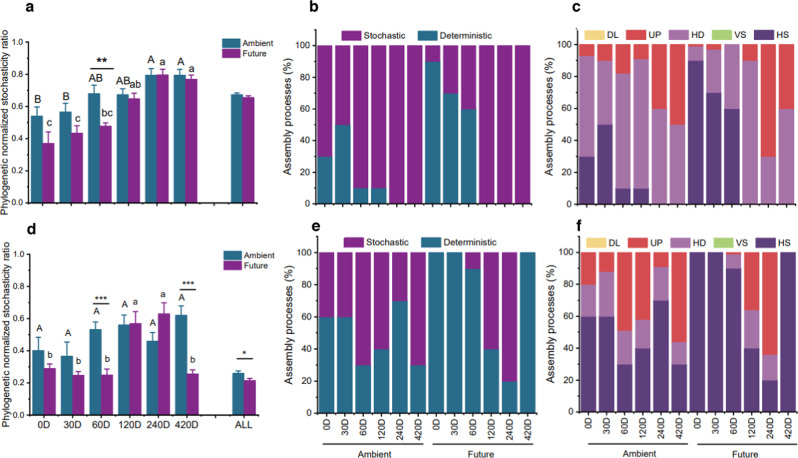
Impact of future climate and time on assembly processes of straw-inhabiting microorganisms. Ecological stochasticity in potential (**a**–**c**) bacterial and (**d**–**f**) fungal community assembly estimated by the phylogenetic normalized stochasticity ratio (pNST) and RC_bray_ index based on Bray–Curtis distance. The value of 0.5 as the boundary point between more deterministic (<0.5) and more stochastic (>0.5) assembly. Differences between ambient and future climates were examined by *t* test (*p < 0.05; **, *p* < 0.01; ***p < 0.001). The relative contributions (%) of the community assembly processes based on pNST (**c**, **d**) and RC_bray_ (**e**, **f**) in shaping microbial communities. Different upper and lower letters indicate the significant differences among decomposition phases under ambient and future climate regimes, respectively. HS homogeneous selection, VS variable selection, HD homogenizing dispersal, UP undominated process, DL dispersal limitation.

The pNST and RC_bray_ were calculated based on the null model analysis to quantify ecological stochasticity. In the early phase of decomposition (0–60 D), the bacterial community was primarily shaped by a deterministic process under the future climate regime as compared with the ambient one (Fig. 5a, b). More notably, after 60 days of field incubation, the pNST value under ambient climate conditions was significantly higher than under future climate conditions (*p* < 0.01; Fig. 5a). In the later phase of decomposition (120–420 D), the relative importance of ecological stochasticity (homogenizing dispersal followed by drift) increased sharply under both climate regimes (Fig. 5c). In contrast to bacteria, the fungal communities were predominantly governed by deterministic processes under both climate regimes; however, under future climate conditions, the relative importance of deterministic processes increased significantly (Fig. 5d, e). In the early phase, the stochasticity of fungal communities was higher under the ambient climate regime than under the future one (Fig. 5e). The drift (undominated process) was responsible primarily for the assembly and turnover of fungal communities under ambient treatment (from 9% to 56%) (Fig. 5f). After 420 days of field incubation, the pNST value in the future climate was strikingly lower than that in the ambient climate (*p* < 0.001, Fig. 5d). Our results indicated that future climate elicited deterministic filtering for fungal phylogenetic composition during the whole decomposition process and for bacteria only during the early phase of straw decomposition.

### Future climate conditions alter molecular ecological networks (MENs) complexity and composition in decomposing wheat straw

**Fig. 6:**
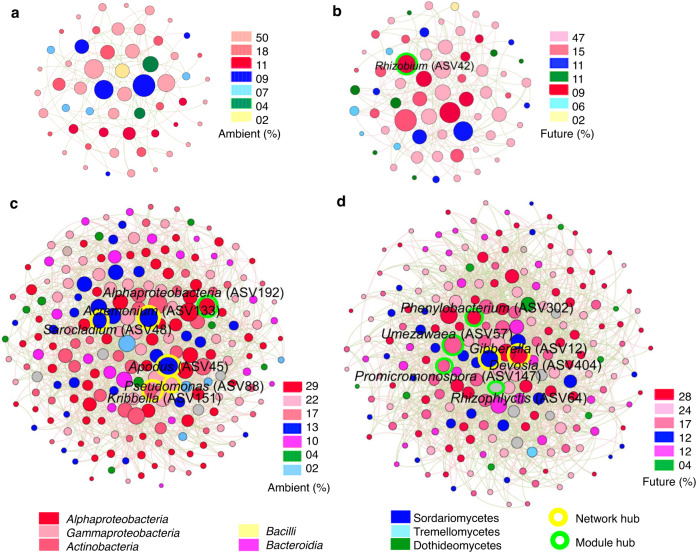
Microbial ecological networks (MENs) under ambient and future climate regimes. Microbial interaction networks at (**a**, **b**) early and (**c**, **d**) later phases of wheat straw decomposition. The size of circles roughly represents relative scores of betweenness centrality. Nodes were colored according to taxonomic group.

We constructed MENs for bacteria and fungi to explore the effect of future climate conditions on the microbial interactions in wheat straw at early (0–60 D) and later (120–420 D) phases of decomposition (Fig. 6). The overall topological properties indicated that future climate significantly affected MENs only at the early phase of decomposition. During the early (0–60 D) phase, future climate network showed a significant increase in average clustering coefficient (0.13), and average connectivity (5.06) as compared with ambient one, indicating that future climate regime triggered the microbial network complexity (Table 1). Unexpectedly, this pattern disappeared at the later (120–420 D) phase of decomposition. In addition, the taxonomic composition of microbial MENs was clearly different between the two climate regimes, especially at the early phase of decomposition (Supplementary Tables S14–S17). For instance, during the early phase (0–60 D), fungi were placed at the central topological positions in ambient climate network, while members of *Actinobacteria* and *Alphaproteobacteria* (*Rhizobium* ASV 24) showed the highest community betweenness and hubs for future climate network (Fig. 6a, b).

**Table 1:** Topological characteristics of microbial ecological networks (MENs) under ambient and future climate regimes and their corresponding random networks.

	Empirical networks								Random networks		
	Total nodes	Total links	Positive/Negative links (%)	Module number	Modularity	Average connectivity	Average clustering coefficient	Average path distance (GD)	Average clustering coefficient	Average path distance (GD)	Modularity
Ambient (0–60 D)	56	105	70/30	4	0.409	3.750	0.055 a	3.034	0.075 ± 0.018	3.031 ± 0.074	0.422 ± 0.015
Future (0–60 D)	66	167	59/41	6	0.367	5.061	0.130 b	2.683	0.114 ± 0.021	2.713 ± 0.046	0.352 ± 0.015
Ambient (120–420 D)	219	1179	56/44	6	0.277	10.767	0.095	2.518	0.080 ± 0.006	2.525 ± 0.006	0.248 ± 0.006
Future (120–420 D)	214	1227	65/35	4	0.248	11.467	0.113	2.454	0.105 ± 0.006	2.464 ± 0.007	0.232 ± 0.005

Significant difference in the average cluster coefficient between ambient and future climate regimes was detected based on *t* test.

Investigating the relationships between the microbial network connectivity, wheat straw chemical contents as well as environmental variables during the early phase MENs revealed that: (1) ambient climate MEN was not correlated to any of tested variables, (2) the connectivity of future climate MEN was significantly influenced by straw N, Mg^2+^, P, pH, and moisture contents (*r*_(m)_ = 0.14–0.18; *p* < 0.05) (Table 2). During the late phase of decomposition the connectivity of microbial MENs significantly responded to various factors under different climate regimes (ambient climate: N, Mg^2+^, temperature and precipitation; future climate: C, K^+^, pH).

**Table 2:** The partial Mantel tests on the association between microbial MENs connectivity versus wheat straw chemistry, environmental factors, enzymes activity and wheat straw under ambient and future climate regimes.

Variables			Wheat straw chemistry								Environmental factors			Decomposition-related function					Ecosystem function
			C	N	CN	K^+^	Ca^2+^	Mg^2+^	P	pH	MOI	Temp	ppt	β_glucosidase	N-acetyl-glucosaminidase	Phosphatase	Phenol oxidase	Peroxidase	Straw mass
Ambient	Early (0–60 D)	*r*_(m)_	0.003	0.134	0.122	0.088	0.080	0.034	0.075	0.077	0.144	0.115	0.120	0.099	0.122	0.102	**0.006**	0.096	0.130
		*p*	0.432	0.073	0.084	0.148	0.155	0.321	0.157	0.191	0.062	0.080	0.092	0.117	0.081	0.108	0.418	0.143	0.070
Future	Early (0–60 D)	*r*_(m)_	0.125	0.142	0.139	0.143	0.132	0.149	0.189	0.172	0.146	0.124	0.084	0.143	0.170	0.135	0.183	0.182	0.140
		*p*	0.066	**0.043**	0.055	0.052	0.061	**0.042**	**0.012**	**0.021**	**0.038**	0.055	0.111	**0.047**	**0.033**	0.061	**0.017**	**0.020**	**0.038**
Ambient	Late (120–420 D)	*r*_(m)_	0.049	0.104	0.060	0.024	0.066	0.081	0.038	0.062	0.072	0.096	0.088	**0.034**	**0.047**	**0.029**	0.066	0.076	0.072
		*p*	0.131	**0.009**	0.083	0.270	0.067	**0.036**	0.173	0.077	0.058	**0.006**	**0.014**	0.168	0.128	0.231	0.078	**0.037**	**0.038**
Future	Late (120–420 D)	*r*_(m)_	0.090	0.073	0.076	0.093	0.063	0.063	0.068	0.081	0.052	0.071	0.057	0.092	0.103	0.143	0.090	0.078	0.066
		*p*	**0.027**	0.060	0.051	**0.034**	0.089	0.083	0.083	**0.048**	0.143	0.070	0.101	**0.033**	**0.022**	**0.003**	**0.020**	0.060	0.089

*MOI* soil moisture, *ppt* precipitation.

Bold values represent statistical significance *p* < 0.05.

To unravel the relationship between networks connectivity on decomposition-related function (production of hydrolytic and oxidative enzymes) and ecosystem function (straw decomposition) under both climate regimes, partial Mantel test was performed (Table 2). During the early phase of decomposition, only future climate MEN significantly correlated to both enzymes activity and wheat straw mass [2]. During the late phase, both climate MENs were correlated with enzymes activity or decomposition process (straw mass).

## Discussion

This study represents the first comprehensive temporal investigation of the straw decomposition process in agricultural ecosystems under ambient versus future climate conditions projected over the next 50–80 years in Central Germany. Future climate settings accelerated straw decay rate during the early phase of decomposition. On the other hand, nutrient dynamics as well as microbial extracellular enzymes activity of wheat straw did not differ between ambient and future climate conditions. Moreover, future climate regime increased the relative importance of deterministic processes on straw-associated microorganisms. In addition, future climate settings enhanced the complexity of microbial networks during the early phase of decomposition. Furthermore, the results indicated positive correlations between decomposition process and microbial diversity, community composition, and specific microbial ecological functions. Our findings advance our understanding of the fundamental ecological processes that take place in agroecosystems and the performance of bacteria and fungi as main but not sole potential decomposer organisms.

### Influence of future climate conditions on wheat straw decomposition rate and nutrient dynamics

Straw mass loss was significantly higher in future climate plots (62%) than in ambient climate plots (56%). In line with our first hypothesis, future climate significantly accelerated decomposition rate (*k*) in the early phase (0–60 D) of decomposition as compared with ambient climate; however, future climate regime had no significant effect on *k* at the later phase (120–420 D) of decomposition. In our experiment, the future climate scenario included altering precipitation patterns coupled toan increase in average daily mean temperature by 0.55 °C and an increase in minimum daily temperatures by 1.14 °C. Moreover, precipitation increases in spring and decreases during autumn [35]. We demonstrated that the effects of soil warming and changed precipitation patterns on mass loss varied with the decomposition phase. Increased soil temperature and precipitation accelerated *k* only during the early phase of straw decomposition. Our results are in agreement with a previous study that recorded a positive effect of precipitation on litter mass loss only at the early stages of decomposition, while the precipitation effect was negligible at later stages [65]. Additionally, we observed C and K^+^ leaching during the early phase (0–60 D) of decomposition, followed by immobilization in the later phase under future climate conditions, while both elements were immobilized in straw during the entire decomposition process under ambient climate. During the early phase, there was a rapid loss of water-soluble compounds and carbohydrates (labile C) from the straw [3], and accordingly, we observed a rapid release of C in our experiment. Moreover, K^+^ is not a structural component of wheat straw or bound to any organic compound; therefore, it is also released rapidly, as reported previously [65, 66]. These results suggest that the release of nutrients is coupled with a mass loss under future climate conditions during the early phase of decomposition, particularly with a significant reduction in the straw C/N ratio recorded after 30 days of field incubation in future climate plots. However, we did not detect any significant effect of future climate on recalcitrant C turnover at late phases.

### Influence of future climate conditions on microbial community composition and function in wheat straw and possible contribution to the decomposition process

Our results revealed that neither bacterial nor fungal alpha diversity was influenced by the climate regime. In contrast, in the future climate scenario, only the bacterial community composition was altered. Additionally, we found that temperature, followed by precipitation, best explained the variations in microbial communities. However, their impact on the bacterial community and diversity was stronger than that of fungi. This finding is in agreement with a previous litter decomposition study that showed the resistance of fungal communities to climate change, while bacterial communities were highly responsive to the change [9]. As microbial communities demonstrated a successional pattern over time, their response to temperature and precipitation differed between the early (0–60 D) and later (120–420 D) phases of decomposition. For instance, bacteria are responsive to environmental variables during both phases of decomposition. In contrast, fungal communities were more resilient to environmental factors in the early phase, while responding to temperature and precipitation during the later phase of decomposition. We conclude that the impact of climate change on straw-inhabiting microorganisms is highly influenced by decomposition phase and the structure of decomposing material.

As expected, we observed a positive correlation between bacterial and fungal diversity and enzyme activity, and accordingly, a negative correlation with straw mass. In addition, straw mass was correlated to the community composition of both bacteria and fungi, with a higher correlation with bacterial community during the early phase (0–60 D). In addition, we detected a correlation between bacterial community composition and β-glucosidase, N-acetyl-glucosaminidase, and peroxidase activity during the early and later phases of decomposition. On the other hand, fungi correlated with phosphatase during the early phase of decomposition. In a previous study; Glassman et al. (2018) reported that bacterial communities have a higher impact on litter decomposition rates than fungi [67]. This could have been the case in our study.

Our data showed distinct successional patterns of microbial communities in the wheat residues. The bacterial community was dominated by *Proteobacteria* in the early phase of decomposition, while *Actinobacteria* (relative abundance ~53%) was the dominant phylum in the later phase. Regarding fungal succession, the fungal community was dominated by Ascomycota, while Basidiomycota members increased in relative abundance at 120 (relative abundance ~3%), 240 (~21%), and 420 D (~5%). Ascomycota have a limited ability to degrade lignin and mainly target cellulose and hemicellulose. Basidiomycota, with their ability to degrade recalcitrant lignin-containing straw residues, appears only later in the decomposition process [68]. This succession is driven by a decrease in easily degradable carbohydrates compared to complex compounds such as lignin [69].

Microbial community dynamics is a substantial driver of community functioning and can be used to better understand ecophysiological responses at the community level over decomposition time. Our results revealed that the early phase of decomposition is dominated by potential plant pathogenic fungi, whereas litter, wood, and general (soil, dung, unspecific) saprotrophs increased in the later phase of decomposition. The future climate regime significantly increased the relative abundance of general saprotrophs. Supporting our second hypothesis, we detected a positive correlation between the decomposition process and all saprotrophic groups.

### Influence of future climate on microbial assembly process in wheat straw and possible association with the decomposition process

Although there have been many studies on microbial assembly patterns [19, 20, 23], our study is the first to determine the assembly of potential decomposer microorganisms in litter microhabitats on a temporal scale. The pNST patterns revealed that stochasticity mostly shapes bacterial community assembly, while both stochasticity and determinism shape fungal community assembly in wheat straw under ambient climate. The future climate regime increased the relative importance of homogeneous selection (deterministic processes) on bacteria only in the early phase of decomposition. Additionally, the future climate regime promoted the dominance of homogeneous selection on the fungal community at all investigated decomposition periods, especially during the early (0–60 D) phase and at 420 D of decomposition. These periods are characterized by increased precipitation and soil warming in the manipulated future climate scenario. We conclude that the combined effect of warming and high precipitation increases environmental filtering on phylogenetic microbial assembly in wheat straw with a greater potential effect on fungi than on bacteria. This result is in line with our third hypothesis; however, we noticed that the impact of future climate on the assembly process in straw was time-dependent. The clear significant impact of the future regime appeared specifically at 60 and 420 D. This could be explained by the multidimensional variety of decomposed material (e.g., size, cavities, phloem, or xylem) that created various micro-niches and available nutrients [70] that have a combined effect with climate on the assembly process. Guo et al. demonstrated that warming significantly decreases the relative importance of stochastic processes by 4.6–17.6% in shaping bacterial and fungal communities [71]. The combined effect of warming and drought was found to enhance homogeneous selection and decrease drift in bacterial community assembly in grassland soil [61]. We also demonstrated that phylogenetically diverse bacterial communities exhibit a greater tendency towards stochastic, as recognized in previous studies [25]. In contrast, a low-diversity fungal community is driven by deterministic processes. This explanation was supported by previous observations [20], where the phylogenetic analyses proved that similar taxa coexist to a greater degree than expected by chance and are governed by similar deterministic factors. From our results, we conclude that climate change and extreme environmental stress elicit stronger deterministic processes in phylogenetically different communities. This could be explained by niche selection, which filters out members that are not able to tolerate certain environmental stresses [26]. This niche selection is clearer in more phylogenetically related communities.

Analyzing microbial community assembly patterns can capture aspects of microbial performance; however, it is challenging to determine how community assembly contributes to ecosystem function [72]. We noticed that future climate conditions increase the potential contribution of deterministic processes in microbial assembly coupled with an increase in *k*, especially in the early phase of decomposition, when determinism controlled both bacteria and fungi associated with significant differences in decomposition rate. Our observation contradicts the concept that stochastic drives a more diverse community, pushing the community toward high functionality [25]. In a unique habitat like decomposing litter, microbial subgroups could be deterministically selected from soil to degrade straw components which undergo temporal change [24]. Therefore, this point requires further investigation.

### Influence of future climate on potential microbial interactions in wheat straw and possible contribution to the decomposition process

Microorganisms exhibit complex ecological interactions, which could be synergetic, mutualistic, or antagonistic within a single domain or among domains [73]. Recent studies have shown that climate change alters microbial interactions in soils, as studied by molecular ecological networks (MENs) [29, 31]. Our results supported the fourth hypothesis and indicated that future climate enhanced the complexity of microbial networks in wheat straw only in the early phase of decomposition. The results demonstrate that microbial MENs in wheat straw are more stable under extreme conditions in the early phase than in the later phase of decomposition. Previous studies have shown contradicted influences of climate change on bacterial networks, and Zhou et al. [29] found that prokaryote networks exhibit lower complexity under warming conditions, while Yuan et al. [31] detected a higher complexity and stability of soil bacterial networks in response to experimental warming. In addition, we found that future climate regimes alter the keystone taxa (network hubs, module hubs) within networks. For instance, *Rhizobium* was detected as a module hub in future climate networks in the early phase of decomposition. It has been widely proven that N-fixing bacteria accumulate N during decomposition, which promotes the succession of other decomposers [74]. Therefore, we concluded that *Rhizobium* plays a complementary role in facilitating the decomposition process. We also found that future climate regimes alter the correlation between network connectivity and environmental factors. Our results demonstrated that the impact of future climate on microbial networks and their influence by environmental factors depends on the decomposition phase.

Microbial interaction networks play a key role in ecosystem functioning [73]. In our study, we found that the correlation of microbial networks connectivity and the straw decomposition process was influenced by climate and the decomposition phase. We observed that during the early phase of decomposition, only future climate MEN were correlated with enzyme activity and straw decomposition. In contrast, our results revealed that later phase bacterial and fungal networks correlate to both functions under ambient and future climate conditions. Taken together, we demonstrated that highly connective and complex networks have a higher potential to be associated with the decomposition process. In line with our fourth hypothesis, this change in microbial interactions could be one reason for the significantly higher *k* detected under future climate conditions in the early phase of decomposition.

## Supplementary information

Supplementary Figures

Supplementary Tables

## Data Availability

Raw sequences were deposited in the Sequence Read Archive (SRA) operated by the National Center for Biotechnology Information (NCBI) under BioProject accession number: PRJNA807133.
