# Supplementary material for: Future climate conditions accelerate wheat straw decomposition alongside altered microbial community composition, assembly patterns, and interaction networks
Source: ISME J. 2022 Nov 9;17(2):238–51. doi: 10.1038/s41396-022-01336-2 (PMC9860053; doi:10.1038/s41396-022-01336-2)
Supplement: Supplementary file 1 — Supplementary Figures [file 41396_2022_1336_MOESM1_ESM.docx]

**Title**

**Future climate conditions accelerate wheat straw decomposition alongside altered microbial community composition, assembly patterns, and interaction networks.**

Sara Fareed Mohamed Wahdan ^1,2,*^ , Li Ji ^1,3,*^, Martin Schädler ^4,5^, Yuting Wu ^6,7^, Chakriya Sansupa ^8^, Benjawan Tanunchai ^1^, François Buscot ^1,4,‡^, Witoon Purahong ^1, ‡^

sarah_wahdan@science.suez.edu.eg (Sara F. M. Wahdan); jlnefu@hotmail.com (Li Ji); [martin.schaedler@ufz.de](mailto:martin.schaedler@ufz.de) (Martin Schädler); [yutingwu@mail.npust.edu.tw](mailto:yutingwu@mail.npust.edu.tw) (Yuting Wu); [chakriya.sansupa@gmail.com](mailto:chakriya.sansupa@gmail.com) (Chakriya Sansupa); [tanunchai.benjawan@ufz.de](mailto:tanunchai.benjawan@ufz.de) (Benjawan Tanunchai); [françois.buscot@ufz.de](mailto:francois.buscot@ufz.de) (François Buscot); [witoon.purahong@ufz.de](mailto:witoon.purahong@ufz.de) (Witoon Purahong)

^1^ Department of Soil Ecology, UFZ-Helmholtz Centre for Environmental Research, Theodor-Lieser-Str. 4, 06120 Halle (Saale), Germany

^2^ Department of Botany & Microbiology, Faculty of Science, Suez Canal University, 41522 Ismailia, Egypt

^3^ School of Forestry, Central South University of Forestry and Technology, 410004, Chagsha, P.R.China

^4^ German Centre for Integrative Biodiversity Research (iDiv) Halle-Jena-Leipzig, Deutscher Platz 5e, 04103 Leipzig, Germany

^5^ Department of Community Ecology, UFZ-Helmholtz Centre for Environmental Research, Theodor-Lieser-Str. 4, 06120 Halle (Saale), Germany

^6^ Department of Forestry, National Pingtung University of Science and Technology, 91201 Pingtung, Taiwan

^7^ Department of Biomedical Science and Environmental Biology, Kaohsiung Medical University, 80708 Kaohsiung, Taiwan, ROC

^8^ Department of Biology, Faculty of Science, Chiang Mai University, 50200 Chiang Mai, Thailand

Correspondence goes to:

Sara Fareed Mohamed Wahdan

[sarah_wahdan@science.suez.edu.eg](mailto:sarah_wahdan@science.suez.edu.eg)

Witoon Purahong

[witoon.purahong@ufz.de](mailto:witoon.purahong@ufz.de)

Theodor-Lieser-Strasse 4 | D-06120 Halle/Saale, Germany

Tel: +49 345 558 5207, Fax: +46 345 558 5449

^‡^ Senior Authors.

*Equal contribution

| **Author** | **ORCID** |
| --- | --- |
| Sara Fareed Mohamed Wahdan | <https://orcid.org/0000-0002-0091-9717> |
| Martin Schädler | <https://orcid.org/0000-0001-9700-0311> |
| Benjawan Tanunchai | <https://orcid.org/0000-0002-6543-3574> |
| François Buscot | <https://orcid.org/0000-0002-2364-0006> |
| Witoon Purahong | <https://orcid.org/0000-0002-4113-6428> |


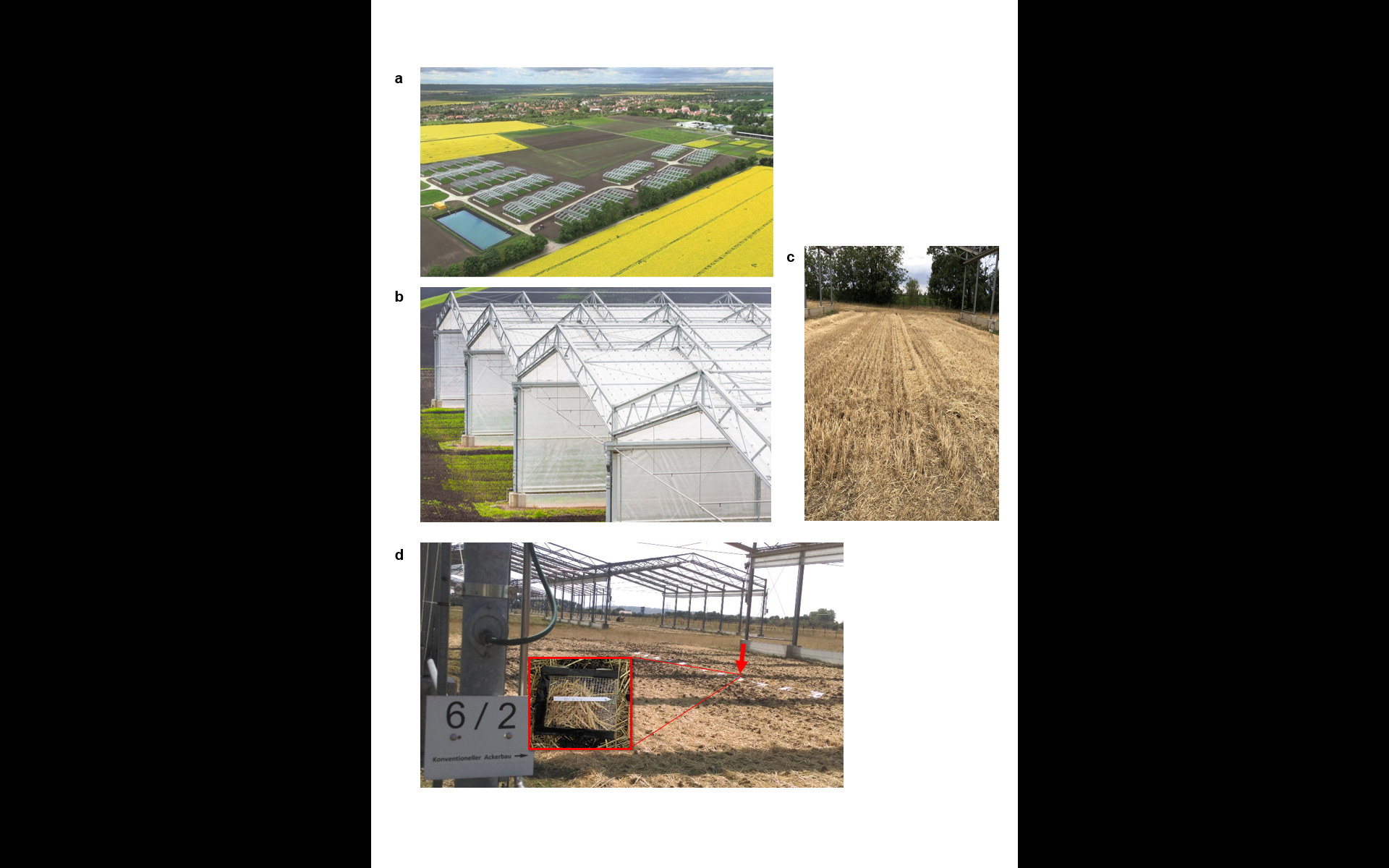


**Supplementary Figure S1**

**a** Aerial view for the Global Change Experimental Facility (GCEF) field research station of the Helmholtz Centre for Environmental Research in Bad Lauchstädt, Saxony-Anhalt, Germany, photo taken by Tricklabor Berlin/Service Drone. **b** Closed shelters and panels of the future climate plots of the GCEF, photo taken by UFZ/ André Künzelmann **c** One of the GCEF field plots after harvesting of winter wheat. **d** Litterbags at the soil surface at the beginning of the experiment.


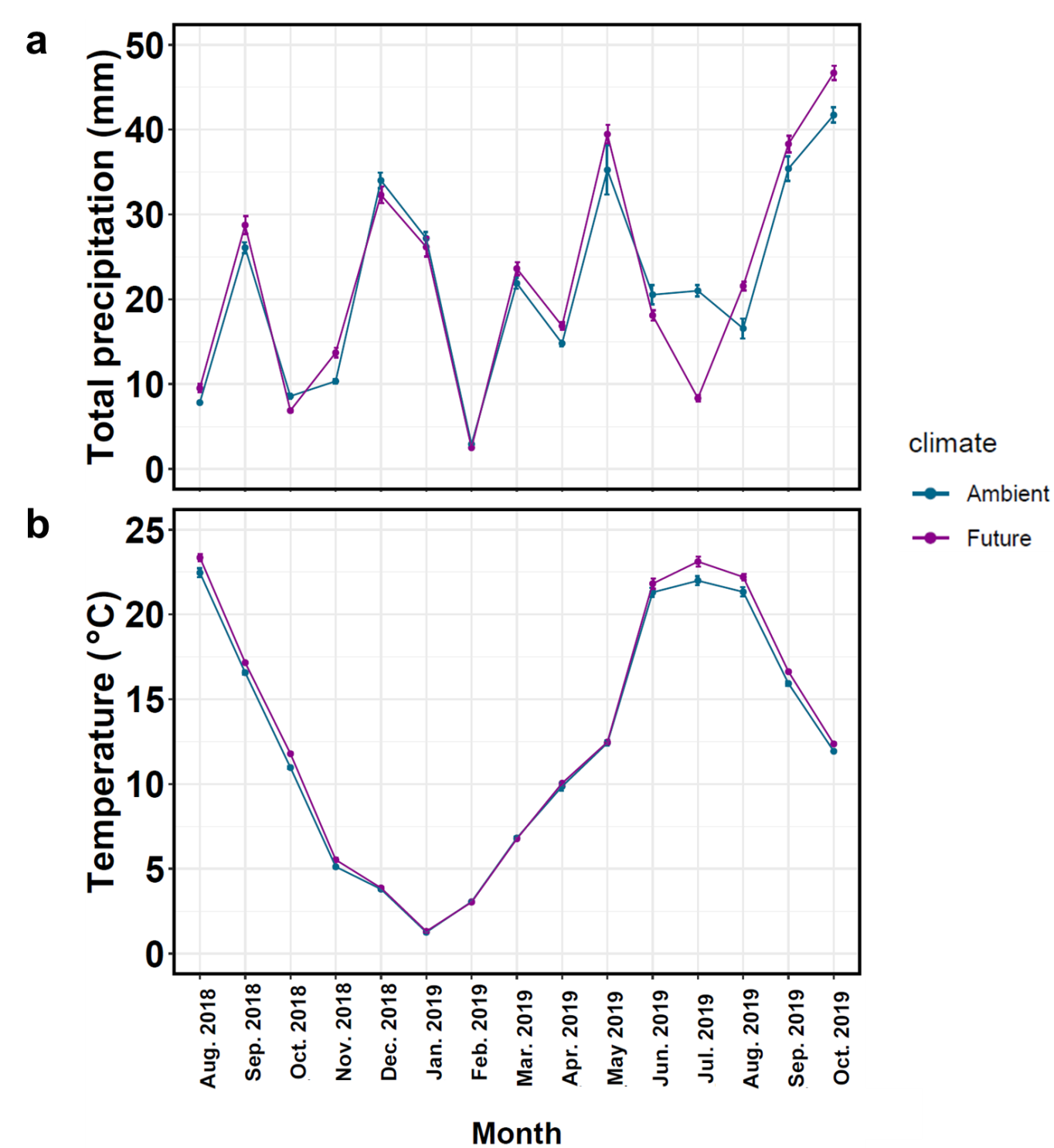


**Supplementary Figure S2**

**a** Total monthly precipitation and **b** Average monthly temperature of the GCEF plots subjected to ambient and future climate conditions.


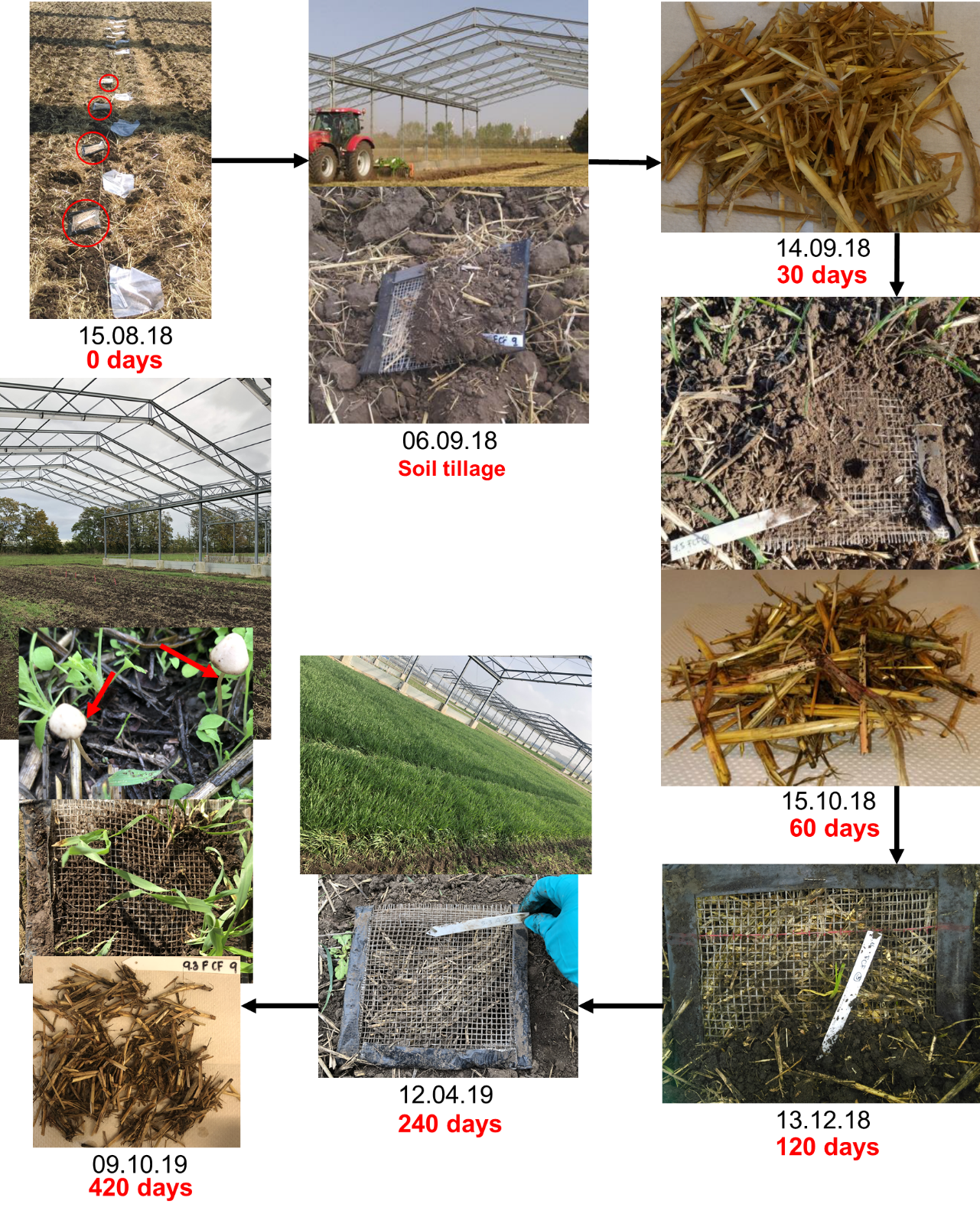


**Supplementary Figure S3**

Experimental layout showing field incubation of litterbags during the subsequent crop and 6 sampling times (0, 30, 60, 120, 240, and 420 days). Litterbags were located at the soil surface at the beginning of the experiment (0 day). After soil tillage, we placed the litterbags below ground to simulate natural conditions for decomposing wheat litter. At 420 D, we noticed a growth of Basidiomycotean fungi in the field plots.


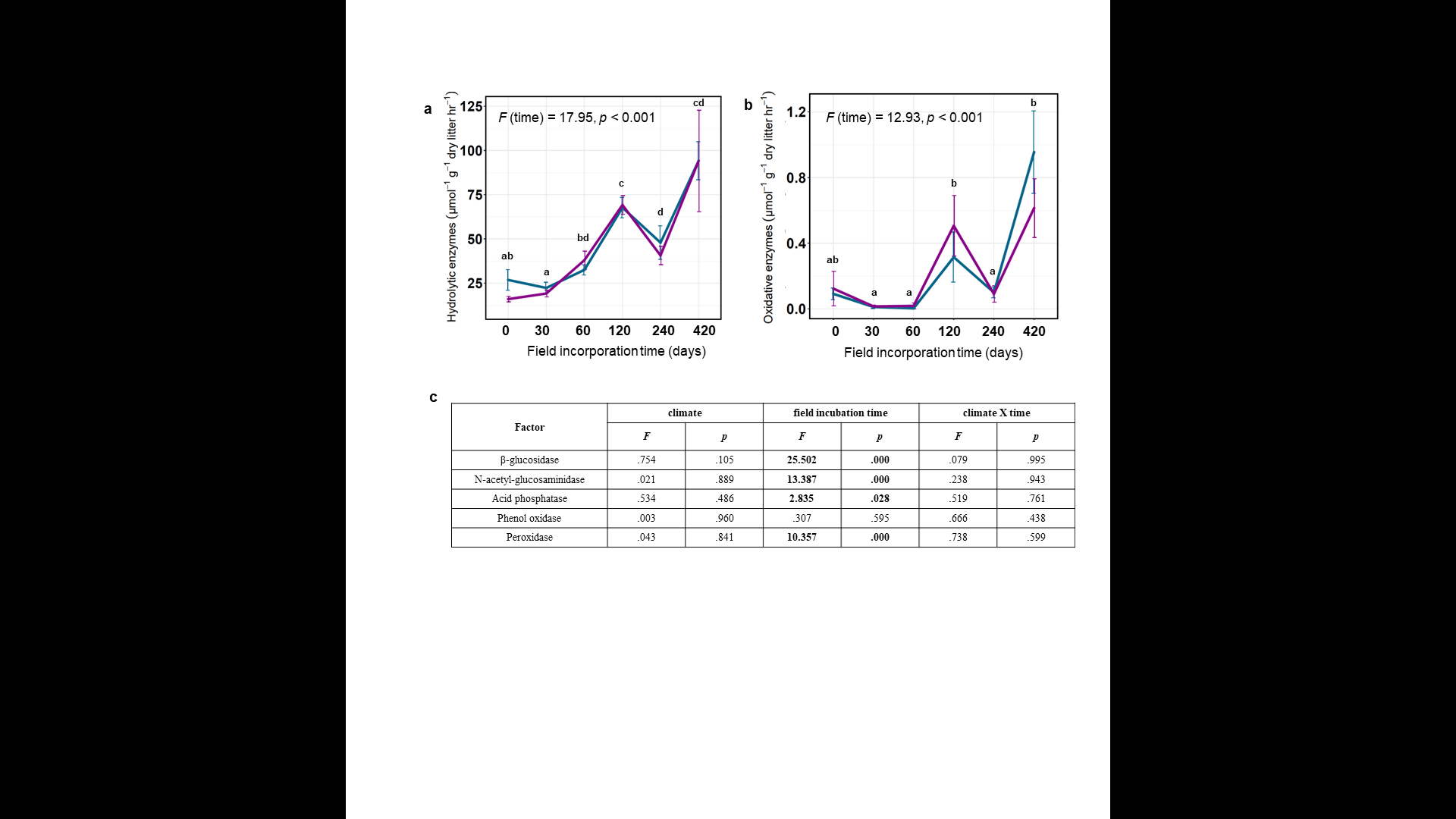


**Supplementary Figure S4**

Dynamics of **a** hydrolytic and **b** oxidative enzyme activities over 420 days in wheat straw. Values in a given graph (means ± SE, n=5) labeled with different letters differ significantly (repeated-measured ANOVA, followed by Bonferroni, *p* < 0.05). **c** Effects of climate regime and time on each of the hydrolytic and oxidative enzyme activities in wheat straw over 420 days of decomposition in field.


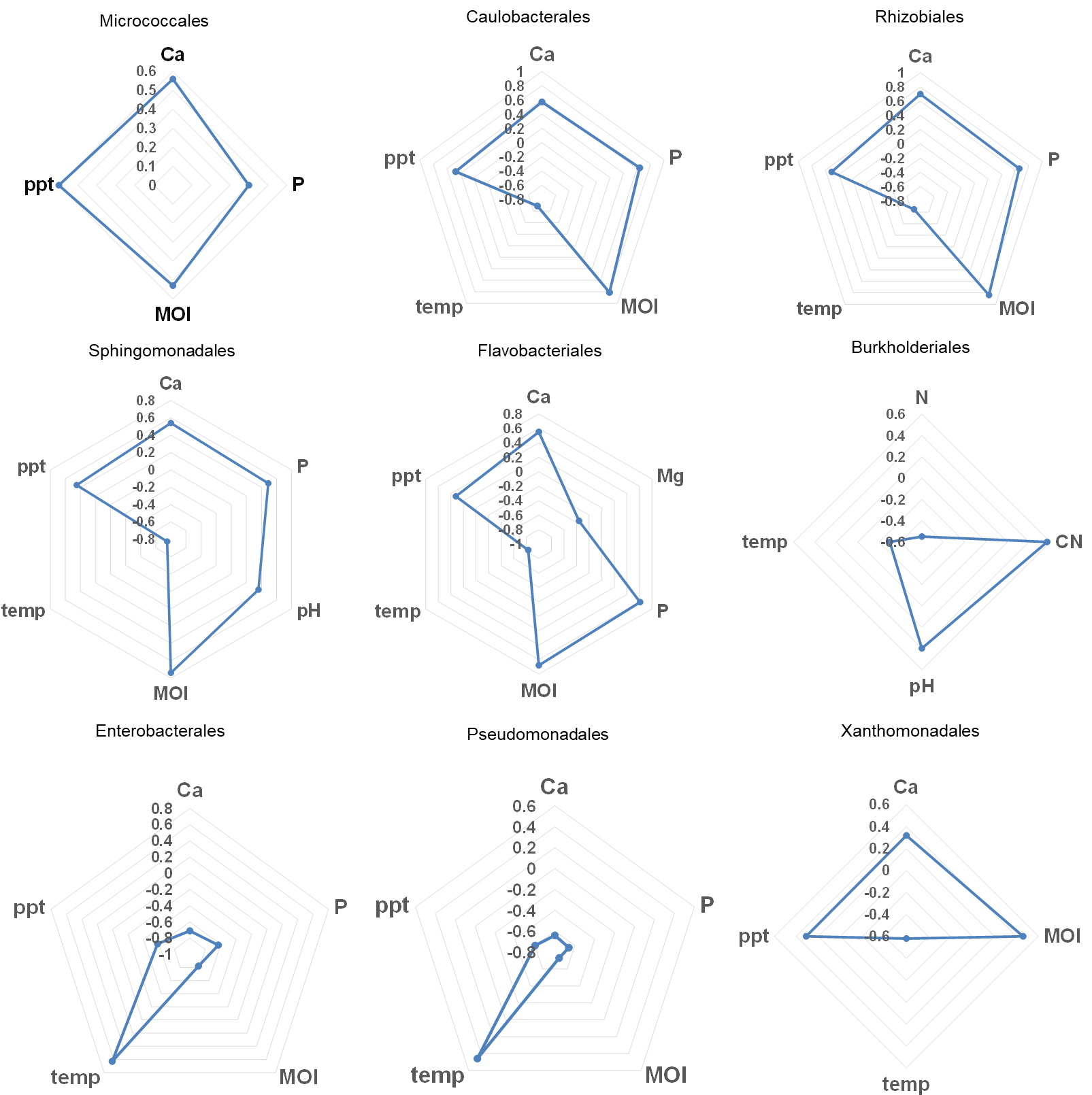


**Supplementary Figure S5**

A radar chart showing the significant positive correlations between the most abundant bacterial orders with soil and straw chemical content factors (*p* < 0.05; Spearman’s rank correlation analysis).

**
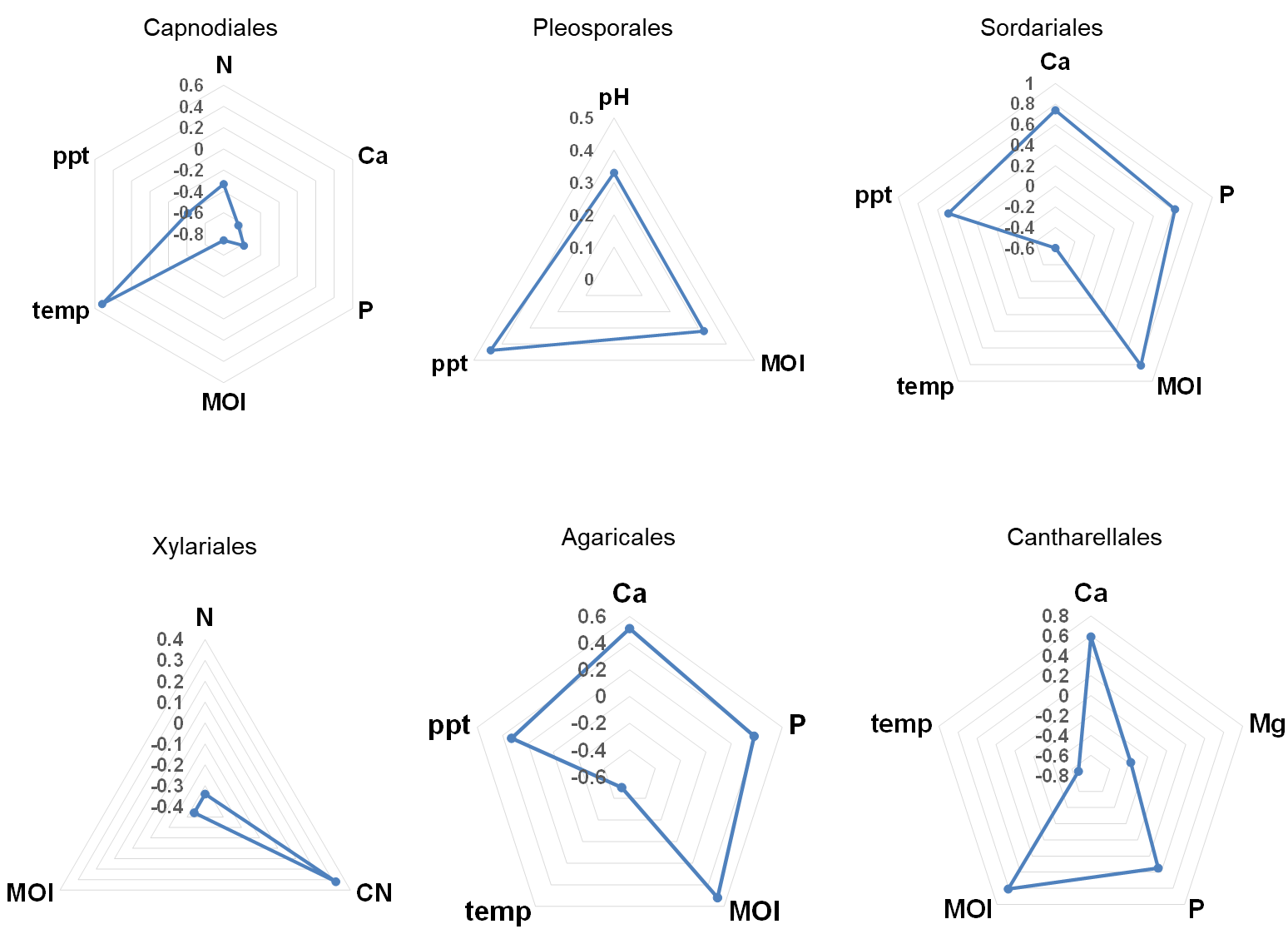
**

**Supplementary Figure S6**

A radar chart showing the significant positive correlations between the most abundant fungal orders with soil and straw chemical content factors (p < 0.05; Spearman’s rank correlation analysis).
