# Supplementary material for: Future climate conditions accelerate wheat straw decomposition alongside altered microbial community composition, assembly patterns, and interaction networks
Source: ISME J. 2022 Nov 9;17(2):238–51. doi: 10.1038/s41396-022-01336-2 (PMC9860053; doi:10.1038/s41396-022-01336-2)
Supplement: Supplementary file 2 — Supplementary Tables [file 41396_2022_1336_MOESM2_ESM.docx]

**Title**

**Future climate conditions accelerate wheat straw decomposition alongside altered microbial community composition, assembly patterns, and interaction networks.**

Sara Fareed Mohamed Wahdan ^1,2,*^ , Li Ji ^1,3,*^, Martin Schädler ^4,5^, Yuting Wu ^6,7^, Chakriya Sansupa ^8^, Benjawan Tanunchai ^1^, François Buscot ^1,4,‡^, Witoon Purahong ^1, ‡^

sarah_wahdan@science.suez.edu.eg (Sara F. M. Wahdan); jlnefu@hotmail.com (Li Ji); [martin.schaedler@ufz.de](mailto:martin.schaedler@ufz.de) (Martin Schädler); [yutingwu@mail.npust.edu.tw](mailto:yutingwu@mail.npust.edu.tw) (Yuting Wu); [chakriya.sansupa@gmail.com](mailto:chakriya.sansupa@gmail.com) (Chakriya Sansupa); [tanunchai.benjawan@ufz.de](mailto:tanunchai.benjawan@ufz.de) (Benjawan Tanunchai); [françois.buscot@ufz.de](mailto:francois.buscot@ufz.de) (François Buscot); [witoon.purahong@ufz.de](mailto:witoon.purahong@ufz.de) (Witoon Purahong)

^1^ Department of Soil Ecology, UFZ-Helmholtz Centre for Environmental Research, Theodor-Lieser-Str. 4, 06120 Halle (Saale), Germany

^2^ Department of Botany & Microbiology, Faculty of Science, Suez Canal University, 41522 Ismailia, Egypt

^3^ School of Forestry, Central South University of Forestry and Technology, 410004, Chagsha, P.R.China

^4^ German Centre for Integrative Biodiversity Research (iDiv) Halle-Jena-Leipzig, Deutscher Platz 5e, 04103 Leipzig, Germany

^5^ Department of Community Ecology, UFZ-Helmholtz Centre for Environmental Research, Theodor-Lieser-Str. 4, 06120 Halle (Saale), Germany

^6^ Department of Forestry, National Pingtung University of Science and Technology, 91201 Pingtung, Taiwan

^7^ Department of Biomedical Science and Environmental Biology, Kaohsiung Medical University, 80708 Kaohsiung, Taiwan, ROC

^8^ Department of Biology, Faculty of Science, Chiang Mai University, 50200 Chiang Mai, Thailand

Correspondence goes to:

Sara Fareed Mohamed Wahdan

[sarah_wahdan@science.suez.edu.eg](mailto:sarah_wahdan@science.suez.edu.eg)

Witoon Purahong

[witoon.purahong@ufz.de](mailto:witoon.purahong@ufz.de)

Theodor-Lieser-Strasse 4 | D-06120 Halle/Saale, Germany

Tel: +49 345 558 5207, Fax: +46 345 558 5449

^‡^ Senior Authors.

*Equal contribution

| **Author** | **ORCID** |
| --- | --- |
| Sara Fareed Mohamed Wahdan | <https://orcid.org/0000-0002-0091-9717> |
| Martin Schädler | <https://orcid.org/0000-0001-9700-0311> |
| Benjawan Tanunchai | <https://orcid.org/0000-0002-6543-3574> |
| François Buscot | <https://orcid.org/0000-0002-2364-0006> |
| Witoon Purahong | <https://orcid.org/0000-0002-4113-6428> |

**Supplementary Table S1**

Chemical treatments for conventional croplands (CF) at the GCEF in 2018 and 2019.

| **Date** | **Category** | **Name** | **Active ingredient** | **ratio** |
| --- | --- | --- | --- | --- |
| 26 Mar 2018 | Fertilizer | KAS | Nitrochalk (27% N) | 60 kg ha^-1^ |
| 09 Apr 2018 | Herbicide | Duanti® | 200 g l^-1^ MCPA,20 g l^-1^ clopyralid;40 g l^-1^ fluroxypyr | 3 l ha^-1^ |
| 09 Apr 2018 | Stem stabilizer | Stabilan 720 ® | 558 g l^-1^ Chlormequat | 2.1 l ha^-1^ |
| 20 Apr 2018 | Fungicide | Capalo® | 200 g l^-1^ Fenpropimorph,62.5 g l^-1^ Epoxiconazol, | 1.6 l ha^-1^ |
| 20 Apr 2018 | Stem stabilizer | Medax Top® | 228.9 g l^-1^ Mepiquat, 42.39 g/l Prohexadion | 1 l ha^-1^ |
| 20 Apr 2018 | Additive | Turbo | 21% N | 1 kg ha^-1^ |
| 18 May 2018 | Fungicide | Adexar® | 62.5 g l^-1^ epoxiconazole, 62.6 g l-1 fluxapyroxad | 2 l ha^-1^ |
| 23 July 2018 | Stem stabilizer | Prodax ® | 66.7g kg^-1^ Trinexapac, 42.4g kg-1 Prohexadio | 0.75 kg ha^-1^ |
| 01 March 2019 | Fertilizer | KAS | Nitrochalk (27% N) | 60 kg ha^-1^ |
| 01 April 2019 | Fertilizer | KAS | Nitrochalk (27% N) | 40 kg ha^-1^ |
| 02 April 2019 | Fungicide | Ariane C® | 100 g/l Fluroxypyr  2.5 g/l Florasulam  80 g/l Clopyralid | 1.5 l ha^-1^ |
| 02 April 2019 | Stem stabilizer | Medax Top® | 228.9g l^-1^ Mepiquat, 42.39g/l Prohexadion | 1.5 l ha^-1^ |
| 02 April 2019 | Additive | Turbo | 21% N | 1.5 kg ha^-1^ |
| 16 April 2019 | Fungicide | Capalo® | 200g l^-1^ Fenpropimorph, 62.5g l^-1^ Epoxiconazol, 75g/l Metrafenone | 1.6 l/ha^-1^ |
| 23 July 2019 | Fertilizer | Superphosphate | Superphosphate | 30 kg P ha^-1^ |
| 23 July 2019 | Fertilizer | 60er Kali | Potassium chloride | 110 kg K ha^-1^ |
| 23 July 2019 | Fertilizer | Kieserite | Hydrated magnesium sulfate (25% MgO, 20% S) | 30 kg ha^-1^ |
| 23 July 2019 | fertilizer | KAS | Nitrochalk (27% N) | 40 kg ha^-1^ |

**Supplementary Table S2**

Physicochemical factors of wheat straw residues under ambient and future climate regimes over 420 days.

| **ID** | **climate** | **TP** | **C (%)** | **N (%)** | **C/N (%)** | **k (ppm)** | **Ca (ppm)** | **Mg (ppm)** | **P (ppm)** | **pH** | **MOI** | **temp (°C)** | **Ppt (mm)** | **Straw dry mass (g)** |
| --- | --- | --- | --- | --- | --- | --- | --- | --- | --- | --- | --- | --- | --- | --- |
| W0T1 | Ambient | 0D | 48.02 | 0.872 | 55.06881 | 0.27 | 0.35 | 0.08 | 16.73827 | 5.85 | 5.9 | 23.9899 | 9.1 | 9.41 |
| W0T3 | Ambient | 0D | 47.45 | 0.875 | 54.22857 | 0.01 | 0.42 | 0.26 | 67.15796 | 5.84 | 5.1 | 26.17458 | 9 | 9.49 |
| W0T5 | Ambient | 0D | 50.71 | 0.843 | 60.15421 | 0.13 | 0.39 | 0.03 | 39.37905 | 5.93 | 5.2 | 24.24505 | 8.7 | 9.48 |
| W0T8 | Ambient | 0D | 45.88 | 0.671 | 68.37556 | 0.2 | 0.51 | 0.16 | 66.85572 | 5.86 | 4.3 | 24.45112 | 9.2 | 9.57 |
| W0T10 | Ambient | 0D | 50.43 | 0.999 | 50.48048 | 0.05 | 0.51 | 0.43 | 73.46535 | 5.8 | 6 | 24.29228 | 10 | 9.4 |
| W0T2 | Future | 0D | 49.63 | 0.728 | 68.17308 | 0.48 | 0.46 | 0.11 | 44.21728 | 5.81 | 5.8 | 25.6052 | 8.9 | 9.42 |
| W0T4 | Future | 0D | 50.63 | 0.841 | 60.20214 | 0.04 | 0.41 | 0.19 | 60.81625 | 5.54 | 6 | 26.02678 | 7.2 | 9.4 |
| W0T6 | Future | 0D | 48.52 | 0.698 | 69.51289 | 0.76 | 0.54 | 0.22 | 37.43408 | 5.58 | 5.8 | 25.94966 | 6.5 | 9.42 |
| W0T7 | Future | 0D | 50.74 | 0.851 | 59.62397 | 0.23 | 0.4 | 0.1 | 55.9604 | 5.66 | 6.1 | 25.26158 | 7.5 | 9.39 |
| W0T9 | Future | 0D | 51.23 | 0.786 | 65.17812 | 1.99 | 0.52 | 0.17 | 132.2953 | 5.87 | 6.5 | 25.84412 | 7.1 | 9.35 |
| W1T1 | Ambient | 30D | 50.98 | 0.745 | 68.42953 | 0.48 | 0.53 | 0.16 | 41.22375 | 6.33 | 12.60163 | 19.23919 | 9.3 | 9.063211 |
| W1T3 | Ambient | 30D | 51.98 | 0.813 | 63.93604 | 0.51 | 0.63 | 0.29 | 40.03125 | 6.97 | 12.14575 | 20.23242 | 9.5 | 8.899636 |
| W1T5 | Ambient | 30D | 49.02 | 0.824 | 59.49029 | 0.23 | 0.42 | 0.47 | 43.11097 | 6.64 | 12.42604 | 20.01071 | 9.4 | 8.748639 |
| W1T8 | Ambient | 30D | 50.74 | 0.771 | 65.81064 | 0.23 | 0.58 | 0.16 | 45.49125 | 6.13 | 12.35217 | 19.85231 | 8.3 | 8.992668 |
| W1T10 | Ambient | 30D | 48.95 | 0.729 | 67.14678 | 0.57 | 0.61 | 0.33 | 45.48263 | 6.48 | 11.95815 | 19.87101 | 8.3 | 8.760164 |
| W1T2 | Future | 30D | 49.69 | 0.884 | 56.21041 | 0.04 | 0.67 | 0.21 | 50.93392 | 6.23 | 12.82051 | 21.08128 | 8.7 | 6.616923 |
| W1T4 | Future | 30D | 49.6 | 0.741 | 66.93657 | 0.05 | 0.61 | 0.18 | 31.23449 | 6.36 | 11.08214 | 20.49574 | 8.1 | 9.096297 |
| W1T6 | Future | 30D | 48.33 | 0.829 | 58.29916 | 1.29 | 0.55 | 0.17 | 111.7141 | 6.73 | 12.71077 | 20.37094 | 8.2 | 9.191556 |
| W1T7 | Future | 30D | 47.98 | 0.761 | 63.04862 | 0.08 | 0.56 | 0.27 | 80.29136 | 6.41 | 12.08633 | 20.29239 | 7.8 | 8.791367 |
| W1T9 | Future | 30D | 47.29 | 0.861 | 54.92451 | 0.78 | 0.73 | 0.52 | 46.79012 | 6.22 | 12.22076 | 20.85108 | 7 | 8.979816 |
| W2T1 | Ambient | 60D | 50.63 | 0.708 | 71.5113 | 0.09 | 0.62 | 0.11 | 143.1762 | 6.42 | 29.36428 | 13.62151 | 23 | 8.462159 |
| W2T3 | Ambient | 60D | 51.69 | 0.716 | 72.19274 | 0.26 | 0.54 | 0.36 | 123.3545 | 6.26 | 32.83582 | 14.02878 | 23.3 | 8.301493 |
| W2T5 | Ambient | 60D | 49.83 | 0.65 | 76.66154 | 0.29 | 0.68 | 0.1 | 87.80923 | 6.5 | 39.47624 | 13.94101 | 22.8 | 7.662308 |
| W2T8 | Ambient | 60D | 50.11 | 0.709 | 70.67701 | 0.16 | 0.81 | 0.24 | 119.5606 | 6.53 | 29.35268 | 13.59095 | 21.2 | 7.969018 |
| W2T10 | Ambient | 60D | 49.76 | 0.638 | 77.99373 | 0.31 | 0.4 | 0.23 | 119.8573 | 6.57 | 32.93085 | 13.6657 | 21.9 | 7.659297 |
| W2T2 | Future | 60D | 48.57 | 0.607 | 80.01647 | 0.02 | 0.58 | 0.01 | 151.5136 | 5.96 | 31.67702 | 14.37471 | 25.1 | 7.255901 |
| W2T4 | Future | 60D | 51.05 | 0.684 | 74.6345 | 0.04 | 0.49 | 0.13 | 63.07531 | 6.51 | 35.38111 | 14.69384 | 28.1 | 7.612105 |
| W2T6 | Future | 60D | 50.89 | 0.769 | 66.17685 | 0.16 | 0.88 | 0.17 | 177.7228 | 6.79 | 45.27778 | 14.48479 | 24.4 | 7.486 |
| W2T7 | Future | 60D | 45.8 | 0.663 | 69.07994 | 0.12 | 0.61 | 0.03 | 135.4198 | 6.46 | 39.29336 | 14.67135 | 23.7 | 7.22409 |
| W2T9 | Future | 60D | 50.04 | 0.699 | 71.58798 | 0.1 | 0.62 | 0.22 | 159.8519 | 6.79 | 44.30642 | 14.67243 | 24.1 | 7.056377 |
| W3T1 | Ambient | 120D | 53.73 | 0.604 | 88.95695 | 0.01 | 0.9 | 0.03 | 142.6808 | 6.42 | 70.16275 | 3.502899 | 19.7 | 6.743219 |
| W3T3 | Ambient | 120D | 49.38 | 0.632 | 78.13291 | 0.34 | 0.55 | 0.11 | 154.3781 | 6.44 | 72.90179 | 3.66975 | 18.9 | 7.554982 |
| W3T5 | Ambient | 120D | 46.76 | 0.701 | 66.70471 | 0.64 | 0.82 | 0.2 | 38.52244 | 7.07 | 82.9006 | 3.77578 | 20.2 | 7.703278 |
| W3T8 | Ambient | 120D | 46.34 | 0.735 | 63.04762 | 0.47 | 0.89 | 0.08 | 83.2625 | 7.04 | 81.9531 | 3.629528 | 19.8 | 6.789243 |
| W3T10 | Ambient | 120D | 50.4 | 0.707 | 71.28713 | 0.08 | 0.75 | 0.16 | 99.77792 | 6.82 | 78.70944 | 3.967289 | 18.4 | 7.130208 |
| W3T2 | Future | 120D | 50.59 | 0.603 | 83.89718 | 0.43 | 0.68 | 0.04 | 143.536 | 6.42 | 69.60212 | 3.874722 | 18.6 | 7.325889 |
| W3T4 | Future | 120D | 50.08 | 0.708 | 70.73446 | 0.09 | 0.8 | 0.02 | 165.1247 | 6.35 | 59.86547 | 4.011639 | 22.1 | 5.361973 |
| W3T6 | Future | 120D | 47.85 | 0.592 | 80.8277 | 0.58 | 0.93 | 0.1 | 68.09577 | 6.53 | 79.71979 | 4.448647 | 21.1 | 6.799954 |
| W3T7 | Future | 120D | 52.71 | 0.772 | 68.2772 | 0.54 | 0.76 | 0.11 | 187.5746 | 6.65 | 79.442 | 3.730694 | 21.7 | 6.558003 |
| W3T9 | Future | 120D | 48.81 | 0.639 | 76.38498 | 0.11 | 0.63 | 0.08 | 63.54602 | 6.8 | 71.16771 | 3.930199 | 19.6 | 7.259971 |
| W4T1 | Ambient | 240D | 49.33 | 0.862 | 57.22738 | 0.54 | 0.84 | 0.09 | 90.43657 | 5.92 | 50.69519 | 8.150986 | 8.9 | 6.133519 |
| W4T3 | Ambient | 240D | 49.41 | 0.784 | 63.02296 | 0.09 | 0.98 | 0.15 | 150.611 | 6.36 | 57.7798 | 7.589458 | 8.2 | 5.779945 |
| W4T5 | Ambient | 240D | 49.9 | 0.717 | 69.59554 | 0.37 | 0.59 | 0.19 | 147.4321 | 5.89 | 48.90511 | 7.484639 | 9.5 | 6.177372 |
| W4T8 | Ambient | 240D | 51.75 | 0.722 | 71.6759 | 0.61 | 0.63 | 0.1 | 171.6708 | 6.18 | 67.52381 | 7.647444 | 9.3 | 4.572648 |
| W4T10 | Ambient | 240D | 49.74 | 0.78 | 63.76923 | 0.35 | 0.72 | 0.19 | 197.2783 | 6.49 | 68.37979 | 7.942154 | 7.8 | 4.743031 |
| W4T2 | Future | 240D | 49.16 | 0.664 | 74.03614 | 0.03 | 0.95 | 0.11 | 81.94431 | 6.05 | 53.8533 | 7.606042 | 12.7 | 6.068292 |
| W4T4 | Future | 240D | 49.36 | 0.689 | 71.64006 | 0.79 | 0.36 | 0.24 | 194.1337 | 6.35 | 71.01045 | 7.731264 | 12.6 | 5.125352 |
| W4T6 | Future | 240D | 51.54 | 0.587 | 87.80239 | 0.37 | 0.83 | 0.03 | 178.2836 | 5.98 | 62.64428 | 7.774875 | 12.1 | 4.676936 |
| W4T7 | Future | 240D | 51.77 | 0.945 | 54.78307 | 0.55 | 1.15 | 0.1 | 193.7032 | 6.34 | 61.55462 | 7.743107 | 15.2 | 4.940231 |
| W4T9 | Future | 240D | 52.02 | 0.84 | 61.92857 | 0.28 | 0.67 | 0.13 | 159.1584 | 6.56 | 50.99541 | 7.84681 | 12.8 | 4.640735 |
| W5T1 | Ambient | 420D | 55.43 | 1.171 | 47.33561 | 0.43 | 0.84 | 0.19 | 207.3134 | 6.57 | 84.52891 | 13.63111 | 67.1 | 3.519673 |
| W5T3 | Ambient | 420D | 49.49 | 1.08 | 45.82407 | 0.03 | 1.09 | 0.85 | 183.5448 | 6.05 | 89.13223 | 13.50907 | 59.6 | 4.59924 |
| W5T5 | Ambient | 420D | 49.77 | 1.01 | 49.27723 | 0.46 | 1.21 | 0.27 | 172.0274 | 6.22 | 82.02133 | 13.57882 | 65.3 | 4.224987 |
| W5T8 | Ambient | 420D | 49.23 | 0.996 | 49.42771 | 0.13 | 0.93 | 0.24 | 166.3682 | 6.23 | 84.2463 | 13.74922 | 67.6 | 4.626861 |
| W5T10 | Ambient | 420D | 50.15 | 1.184 | 42.35642 | 0.39 | 1.07 | 0.16 | 180.1617 | 6.3 | 80.8136 | 13.77007 | 59.1 | 3.87949 |
| W5T2 | Future | 420D | 53.19 | 0.936 | 56.82692 | 0.1 | 1.04 | 0.18 | 100.9676 | 6.65 | 79.2849 | 14.42692 | 66.9 | 4.592537 |
| W5T4 | Future | 420D | 50.97 | 0.909 | 56.07261 | 0.76 | 0.77 | 0.04 | 170.9453 | 5.65 | 84.23237 | 14.15692 | 65.2 | 2.778257 |
| W5T6 | Future | 420D | 49.4 | 1.085 | 45.52995 | 0.63 | 0.94 | 0.29 | 117.7122 | 5.73 | 81.74666 | 14.15015 | 63.7 | 2.880378 |
| W5T7 | Future | 420D | 51.55 | 1.195 | 43.13808 | 0.12 | 1.55 | 0.21 | 159.8759 | 6.57 | 86.5512 | 13.96925 | 61.1 | 3.421375 |
| W5T9 | Future | 420D | 50.27 | 1.016 | 49.47835 | 0.3 | 1.01 | 0.11 | 223.201 | 6.47 | 82.19396 | 14.17995 | 64.3 | 4.145246 |

**Supplementary Table S3**

Dynamics of hydrolytic and oxidative enzyme activities over 420 days in wheat straw under ambient and future climate regimes. Enzyme activity was expressed as nmol activity h^−1^ g dry straw^−1^.

| **ID** | **climate** | **TP** | **glucosidase** | ***N*-acetyl-glucosaminidase** | **Phosphatase** | **Phenol oxidase** | **Peroxidase** |
| --- | --- | --- | --- | --- | --- | --- | --- |
| W0T1 | Ambient | 0D | 7464.866 | 3598.763 | 12043.81 | 116.7641 | 0 |
| W0T3 | Ambient | 0D | 10501.76 | 6147.841 | 9182.561 | 0 | 16.07778 |
| W0T5 | Ambient | 0D | 5215.729 | 3331.634 | 7357.356 | 0.333761 | 1.174348 |
| W0T8 | Ambient | 0D | 21252.93 | 8390.786 | 19482.51 | 182.1366 | 0 |
| W0T10 | Ambient | 0D | 7945.517 | 4128.609 | 7799.962 | 21.18102 | 119.4814 |
| W0T2 | Future | 0D | 5832.883 | 1760.963 | 6590.994 | 9.454618 | 42.93143 |
| W0T4 | Future | 0D | 7003.622 | 2811.027 | 8564.727 | 10.48454 | 0 |
| W0T6 | Future | 0D | 5896.023 | 2142.418 | 6008.676 | 0 | 0 |
| W0T7 | Future | 0D | 4490.008 | 1915 | 5988.336 | 12.28926 | 0 |
| W0T9 | Future | 0D | 9731.211 | 3350.478 | 7651.222 | 542.0705 | 0 |
| W1T1 | Ambient | 30D | 6918.868 | 1763.703 | 3901.78 | 48.95418 | 0 |
| W1T3 | Ambient | 30D | 11068.78 | 4031.033 | 7153.443 | 0 | 0 |
| W1T5 | Ambient | 30D | 12578.71 | 4509.512 | 8127.084 | 7.975401 | 0 |
| W1T8 | Ambient | 30D | 13039.36 | 4840.458 | 8083.945 | 0 | 0 |
| W1T10 | Ambient | 30D | 11738.17 | 5010.459 | 8500.962 | 0 | 0 |
| W1T2 | Future | 30D | 7352.203 | 1532.13 | 3197.524 | 0 | 0 |
| W1T4 | Future | 30D | 10712.32 | 2737.309 | 6482.198 | 2.991699 | 0 |
| W1T6 | Future | 30D | 12025.24 | 3185.799 | 8249.672 | 0 | 0 |
| W1T7 | Future | 30D | 9576.481 | 3449.083 | 6589.769 | 41.49134 | 0 |
| W1T9 | Future | 30D | 10844.98 | 3255.436 | 6339.122 | 30.14487 | 0 |
| W2T1 | Ambient | 60D | 13008.4 | 7109.379 | 7757.082 | 14.36176 | 0 |
| W2T3 | Ambient | 60D | 13871.11 | 9305.382 | 8572.533 | 0 | 0 |
| W2T5 | Ambient | 60D | 18814.36 | 13304.88 | 9136.587 | 0 | 0 |
| W2T8 | Ambient | 60D | 13321.73 | 5744.403 | 6093.251 | 0 | 0 |
| W2T10 | Ambient | 60D | 14663 | 11870.27 | 9799.38 | 0 | 0 |
| W2T2 | Future | 60D | 12929.24 | 6552.446 | 7248.87 | 0 | 0 |
| W2T4 | Future | 60D | 16536.27 | 8666.137 | 8604.467 | 0 | 0 |
| W2T6 | Future | 60D | 18893.87 | 14269.21 | 11545.4 | 0 | 0 |
| W2T7 | Future | 60D | 12661.96 | 10660.07 | 6710.521 | 33.67888 | 55.91053 |
| W2T9 | Future | 60D | 22697.62 | 17869.83 | 13900.87 | 0 | 0 |
| W3T1 | Ambient | 120D | 24272.41 | 19293.72 | 8406.79 | 29.5745 | 317.543 |
| W3T3 | Ambient | 120D | 35954.19 | 20822.41 | 16129.05 | 36.28295 | 132.8068 |
| W3T5 | Ambient | 120D | 41440.44 | 16583.82 | 11166.39 | 0 | 883.6661 |
| W3T8 | Ambient | 120D | 52694.59 | 21085.23 | 11724.85 | 174.972 | 0 |
| W3T10 | Ambient | 120D | 36211.75 | 15520.43 | 7064.648 | 0 | 0 |
| W3T2 | Future | 120D | 30460.79 | 13768.41 | 10826.07 | 0 | 207.791 |
| W3T4 | Future | 120D | 36076.8 | 20305.11 | 12805.14 | 30.27903 | 76.03334 |
| W3T6 | Future | 120D | 41187.22 | 18907.52 | 19426.51 | 0 | 573.9701 |
| W3T7 | Future | 120D | 37564.49 | 20600.82 | 9549.69 | 6.555392 | 1153.406 |
| W3T9 | Future | 120D | 35094.68 | 27943.72 | 11418.29 | 35.11688 | 449.3485 |
| W4T1 | Ambient | 240D | 12720.94 | 6524.686 | 7938.814 | 0 | 182.1101 |
| W4T3 | Ambient | 240D | 22997.48 | 14255.83 | 8799.685 | 0 | 160.4312 |
| W4T5 | Ambient | 240D | 16312.19 | 9059.231 | 4107.03 | 0 | 0 |
| W4T8 | Ambient | 240D | 31349.69 | 14743.87 | 32463.52 | 0 | 86.13282 |
| W4T10 | Ambient | 240D | 26806.69 | 20238.15 | 11020.57 | 0 | 70.97172 |
| W4T2 | Future | 240D | 14761.85 | 6245.643 | 9425.605 | 0 | 0 |
| W4T4 | Future | 240D | 29797.24 | 12155.92 | 15339.63 | 284.7875 | 0 |
| W4T6 | Future | 240D | 13722.11 | 8793.039 | 8557.332 | 0 | 69.29787 |
| W4T7 | Future | 240D | 24119.07 | 11226.02 | 12521.39 | 58.76844 | 0 |
| W4T9 | Future | 240D | 19326.98 | 11387.37 | 5650.456 | 39.09869 | 0.980466 |
| W5T1 | Ambient | 420D | 72854.24 | 34783.15 | 17130.91 | 77.41252 | 323.2844 |
| W5T3 | Ambient | 420D | 55149.09 | 15021.17 | 11711.22 | 0 | 1732.833 |
| W5T5 | Ambient | 420D | 33668.3 | 10147.29 | 17351.89 | 73.9902 | 1254.081 |
| W5T8 | Ambient | 420D | 59734.59 | 33452.61 | 12517.77 | 530.2326 | 0 |
| W5T10 | Ambient | 420D | 53323.4 | 27681.72 | 16415.11 | 154.5895 | 628.4359 |
| W5T2 | Future | 420D | 24297.5 | 6282.088 | 5166.326 | 52.04538 | 1012.622 |
| W5T4 | Future | 420D | 40542.55 | 17865.18 | 10435.16 | 40.79851 | 473.4809 |
| W5T6 | Future | 420D | 49869.46 | 14335.17 | 13240.03 | 0 | 330.2294 |
| W5T7 | Future | 420D | 111575.9 | 62222.1 | 30222.75 | 188.1017 | 806.8172 |
| W5T9 | Future | 420D | 49242.01 | 26114.26 | 8917.976 | 0 | 163.5461 |

**Supplementary Table S4**

Impact of climate regime, decomposition time, and their interaction on release rate of wheat straw nutrients

| **Factor** | **climate** | | **field incubation time** | | **climate X time** | |
| --- | --- | --- | --- | --- | --- | --- |
|  | ***F*** | ***p*** | ***F*** | ***p*** | ***F*** | ***p*** |
| C/N ratio (%) | 0.947 | 0.359 | **21.751** | **0.000** | 1.146 | 0.352 |
| C (%) | 2.335 | 0.165 | 1.099 | 0.374 | 0.986 | 0.429 |
| N (%) | 0.725 | 0.419 | **39.308** | **0.000** | 0.423 | 0.791 |
| P (mg/g) | 0.408 | 0.541 | **5.812** | **0.001** | 1.453 | 0.240 |
| K (mg/g) | 1.240 | 0.298 | 0.384 | 0.818 | 1.580 | 0.203 |
| Mg (mg/g) | 0.857 | 0.382 | 2.856 | 0.126 | 0.324 | 0.601 |
| Ca (mg/g) | 0.0084 | 0.780 | **9.726** | **0.000** | 0.116 | 0.976 |

**Supplementary Table S5**

Factors contributing to variation in bacterial and fungal communities at early and later phase of straw decomposition. PERMANOVA was applied for the correlation between microbial community compositions (Bray–Curtis dissimilarity distance) and each variable.

| **Variable** | **Bacteria - Early phase** | | **Bacteria - Later phase** | | **Fungi - Early phase** | | **Fungi - Later phase** | |
| --- | --- | --- | --- | --- | --- | --- | --- | --- |
|  | ***R*^2^** | ***p*** | ***R*^2^** | ***p*** | ***R*^2^** | ***p*** | ***R*^2^** | ***p*** |
| **β-glucosidase** | **0.26** | **0.015** | **0.49** | **0.001** | 0.005 | 0.935 | 0.08 | 0.312 |
| **N-acetyl-glucosaminidase** | **0.38** | **0.001** | **0.26** | **0.011** | 0.01 | 0.841 | 0.03 | 0.625 |
| **Acid phosphatase** | 0.0006 | 0.987 | 0.06 | 0.422 | **0.25** | **0.018** | 0.13 | 0.127 |
| **Phenol oxidase** | 0.10 | 0.186 | 0.11 | 0.199 | 0.11 | 0.178 | 0.10 | 0.236 |
| **Peroxidase** | 0.006 | 0.934 | **0.28** | **0.016** | 0.03 | 0.688 | 0.10 | 0.211 |
| **C (%)** | 0.018 | 0.779 | 0.11 | 0.173 | 0.18 | 0.066 | 0.11 | 0.193 |
| **N (%)** | **0.300** | **0.006** | **0.79** | **0.001** | 0.02 | 0.693 | **0.66** | **0.001** |
| **C/N ratio** | **0.35** | **0.003** | **0.70** | **0.001** | 0.04 | 0.541 | **0.57** | **0.001** |
| **K (mg/g)** | 0.09 | 0.248 | 0.008 | 0.878 | 0.001 | 0.987 | 0.0002 | 1.000 |
| **Ca (mg/g)** | 0.18 | 0.061 | **0.44** | **0.001** | 0.16 | 0.094 | **0.34** | **0.001** |
| **Mg (mg/g)** | 0.03 | 0.662 | **0.20** | **0.021** | 0.04 | 0.507 | 0.15 | 0.078 |
| **P (mg/g)** | **0.20** | **0.048** | **0.22** | **0.031** | 0.08 | 0.309 | **0.25** | **0.017** |
| **pH** | **0.50** | **0.001** | **0.43** | **0.002** | **0.28** | **0.013** | **0.29** | **0.010** |
| **MOI** | **0.65** | **0.001** | **0.69** | **0.001** | 0.16 | 0.095 | 0.06 | 0.411 |
| **temp (°C)** | **0.75** | **0.001** | **0.95** | **0.001** | **0.25** | **0.022** | **0.85** | **0.001** |
| **ppt (mm)** | **0.59** | **0.001** | **0.92** | **0.001** | 0.13 | 0.165 | **0.53** | **0.001** |
| **Straw mass (g)** | **0.40** | **0.002** | **0.75** | **0.001** | **0.20** | **0.047** | **0.75** | **0.001** |

**Supplementary Table S6**

Impact of decomposition time and climate regime on the most abundant (relative sequences abundance > 1% of all bacterial sequences) bacterial orders on wheat straw.

| **Order** | **Time** | | **Climate** | | **Statistical test** |
| --- | --- | --- | --- | --- | --- |
|  | ***F* / χ2** | ***p*** | ***F* / *Z*** | ***p*** |  |
| **Micrococcales** | 38.582 | <0.001 | -0.258 | 0.795 | Friedman Test, Mann-Whitney U test |
| **Caulobacterales** | 40.809 | <0.001 | -0.177 | 0.858 | Friedman Test, Mann-Whitney U test |
| **Rhizobiales** | 44.456 | <0.001 | 0 | 1 | Friedman Test, Mann-Whitney U test |
| **Sphingomonadales** | 30.743 | <0.001 | -0.879 | 0.379 | Friedman Test, Mann-Whitney U test |
| **Flavobacteriales** | 45.057 | <0.001 | -0.392 | 0.694 | Friedman Test, Mann-Whitney U test |
| **Burkholderiales** | 19.872 | <0.001 | 9.625 | 0.015 | Repeated measures ANOVA |
| **Enterobacterales** | 46.000 | <0.001 | -0.709 | 0.477 | Friedman Test, Mann-Whitney U test |
| **Pseudomonadales** | 37.543 | <0.001 | -0.354 | 0.722 | Friedman Test, Mann-Whitney U test |
| **Xanthomonadales** | 0.491 | 0.002 | -0.687 | 0.491 | Friedman Test, Mann-Whitney U test |

**Supplementary Table S7**

Impact of decomposition time and climate regime on the top 20 most abundant (relative sequences abundance) bacterial genera colonizing wheat straw

| Order | Time | | Climate | | Statistical test |
| --- | --- | --- | --- | --- | --- |
|  | *F* / χ2 | *p* | *F* / *Z* | *p* |  |
| *Pantoea* (ASV_5) | 10.119 | < 0.001 | 0.668 | 0.437 | Repeated measures ANOVA |
| *Pantoea agglomerans* (ASV_6) | 36.891 | < 0.001 | -0.828 | 0.407 | Friedman test, Mann-Whitney U test |
| *Massilia aurea* (ASV_2) | 26.062 | < 0.001 | 9.723 | 0.014 | Repeated measures ANOVA |
| *Massilia* (ASV_4) | 32.391 | < 0.001 | -2.457 | 0.014 | Friedman test, Mann-Whitney U test |
| *Massilia* (ASV_9) | 42.896 | < 0.001 | -1.201 | 0.229 | Friedman test, Mann-Whitney U test |
| *Massilia* (ASV_7) | 37.029 | < 0.001 | -2.159 | 0.030 | Friedman test, Mann-Whitney U test |
| *Promicromonospora umidemergens* (ASV_1) | 45.586 | < 0.001 | -0.474 | 0.635 | Friedman test, Mann-Whitney U test |
| *Massilia aurea* (ASV_15) | 14.798 | < 0.001 | 3.555 | 0.096 | Repeated measures ANOVA |
| *Promicromonospora umidemergens* (ASV_3) | 48.480 | < 0.001 | -0.284 | 0.776 | Friedman test, Mann-Whitney U test |
| *Massili* (ASV_22) | 39.554 | < 0.001 | -1.705 | 0.088 | Friedman test, Mann-Whitney U test |
| *Pseudomonas* (ASV_16) | 25.963 | < 0.001 | -2.686 | 0.007 | Friedman test, Mann-Whitney U test |
| *Promicromonospora umidemergens* (ASV_8) | 46.882 | < 0.001 | -0.289 | 0.772 | Friedman test, Mann-Whitney U test |
| *Flavobacterium* (ASV_10) | 41.688 | < 0.001 | -2.616 | 0.008 | Friedman test, Mann-Whitney U test |
| *Erwinia gerundensis* (ASV_48) | 29.021 | < 0.001 | -0.702 | 0.482 | Friedman test, Mann-Whitney U test |
| *Pseudomonas* (ASV_11) | 25.073 | < 0.001 | -1.082 | 0.279 | Friedman test, Mann-Whitney U test |
| *Sphingomonas aerolata* (ASV_19) | 41.034 | < 0.001 | -0.126 | 0.899 | Friedman test, Mann-Whitney U test |
| *Pantoea* (ASV_47) | 20.586 | 0.001 | -0.575 | 0.564 | Friedman test, Mann-Whitney U test |
| *Sanguibacter inulinus* (ASV_40) | 37.281 | < 0.001 | -0.714 | 0.475 | Friedman test, Mann-Whitney U test |
| *Pseudomonas mosselii* (ASV_58) | 7.759 | 0.170 | -0.832 | 0.405 | Friedman test, Mann-Whitney U test |
| *Pseudomonas rhizosphaerae* (ASV_31) | 6.098 | < 0.001 | 7.351 | 0.027 | Repeated measures ANOVA |

**Supplementary Table S8**

The relative abundance of potential bacterial genes involved in wheat straw decomposition. A= ambient climate, F= future climate. The functional profiles of bacterial communities were predicted based on the 16S rRNA genes of retrieved bacterial taxa using Tax4Fun according to the KEGG Ortholog groups (KOs).

|  | **KO** | **0A** | **30A** | **60A** | **120A** | **240A** | **420A** | **0F** | **30F** | **60F** | **120F** | **240F** | **420F** |
| --- | --- | --- | --- | --- | --- | --- | --- | --- | --- | --- | --- | --- | --- |
| **Cellulose** | K01222; 6-phospho-beta-glucosidase [EC:3.2.1.86] | 0.000135 | 0.000125 | 8.25E-05 | 7.25E-05 | 3.94E-05 | 3.05E-05 | 0.000116 | 0.000126 | 9.86E-05 | 7.65E-05 | 4.12E-05 | 3.02E-05 |
|  | K01223; 6-phospho-beta-glucosidase [EC:3.2.1.86] | 0.000529 | 0.000543 | 0.000309 | 0.000282 | 0.000125 | 7.09E-05 | 0.000481 | 0.000494 | 0.000378 | 0.000297 | 0.000128 | 6.51E-05 |
|  | K01187; alpha-glucosidase [EC:3.2.1.20] | 0.000598 | 0.000611 | 0.000606 | 0.000632 | 0.000725 | 0.000741 | 0.000582 | 0.000601 | 0.000614 | 0.000641 | 0.000716 | 0.000735 |
|  | K05349; beta-glucosidase [EC:3.2.1.21] | 0.001017 | 0.000943 | 0.000825 | 0.000933 | 0.00128 | 0.001218 | 0.001043 | 0.00098 | 0.000986 | 0.00107 | 0.001253 | 0.00129 |
|  | K05350; beta-glucosidase [EC:3.2.1.21] | 6.9E-05 | 9.53E-05 | 0.000102 | 0.000124 | 0.000143 | 0.000163 | 6.45E-05 | 7.74E-05 | 9.07E-05 | 0.000114 | 0.000142 | 0.000167 |
|  | K01179; endoglucanase [EC:3.2.1.4] | 0.000569 | 0.000543 | 0.000487 | 0.000492 | 0.000438 | 0.000372 | 0.0006 | 0.000551 | 0.00055 | 0.000504 | 0.000434 | 0.00037 |
|  | K01182; oligo-1,6-glucosidase [EC:3.2.1.10] | 1.81E-05 | 4.08E-05 | 1.39E-05 | 1.32E-05 | 1.28E-05 | 1.23E-05 | 1.65E-05 | 2E-05 | 1.69E-05 | 1.93E-05 | 1.37E-05 | 1.29E-05 |
|  | K01199; glucan endo-1,3-beta-D-glucosidase [EC:3.2.1.39] | 1.1E-05 | 1.24E-05 | 1.38E-05 | 1.37E-05 | 2.4E-05 | 2.48E-05 | 1.2E-05 | 1.16E-05 | 1.25E-05 | 1.61E-05 | 2.36E-05 | 2.46E-05 |
| **Hemicellulose** | K11176; IMP cyclohydrolase [EC:3.5.4.10] | 1.99E-07 | 1.69E-07 | 1.28E-07 | 1.37E-07 | 6.42E-08 | 3.6E-08 | 2.22E-07 | 1.84E-07 | 1.45E-07 | 1.33E-07 | 6.15E-08 | 3.21E-08 |
|  | K07405; alpha-amylase [EC:3.2.1.1] | 1.5E-06 | 1.44E-06 | 1.18E-06 | 1.19E-06 | 7.42E-07 | 5.29E-07 | 1.81E-06 | 1.41E-06 | 1.25E-06 | 1.21E-06 | 7.31E-07 | 4.71E-07 |
|  | K07407; alpha-galactosidase [EC:3.2.1.22] | 8.28E-05 | 8.88E-05 | 6.78E-05 | 8.1E-05 | 7.81E-05 | 7.39E-05 | 7.05E-05 | 8.09E-05 | 7.29E-05 | 7.1E-05 | 7.46E-05 | 7.29E-05 |
|  | K01206; alpha-L-fucosidase [EC:3.2.1.51] | 0.000145 | 0.000135 | 0.00011 | 0.000134 | 0.000169 | 0.000145 | 0.000162 | 0.000137 | 0.000151 | 0.000152 | 0.000167 | 0.000159 |
|  | K01191; alpha-mannosidase [EC:3.2.1.24] | 1.92E-05 | 9.09E-05 | 3.51E-05 | 5.77E-05 | 3.67E-05 | 4.49E-05 | 1.37E-05 | 2.69E-05 | 3.35E-05 | 4.76E-05 | 3.95E-05 | 4.37E-05 |
|  | K01224; arabinogalactan endo-1,4-beta-galactosidase [EC:3.2.1.89] | 8.43E-05 | 6.79E-05 | 5.13E-05 | 5.68E-05 | 5.61E-05 | 4.58E-05 | 8.15E-05 | 7.84E-05 | 6.91E-05 | 6.19E-05 | 5.5E-05 | 5.03E-05 |
|  | K12308; beta-galactosidase [EC:3.2.1.23] | 0.000157 | 0.000158 | 0.000144 | 0.000159 | 0.00016 | 0.000149 | 0.000147 | 0.000154 | 0.000164 | 0.000152 | 0.000156 | 0.000153 |
|  | K01192; beta-mannosidase [EC:3.2.1.25] | 0.000147 | 0.000161 | 0.000181 | 0.000185 | 0.000242 | 0.000252 | 0.000162 | 0.000152 | 0.000194 | 0.000205 | 0.000246 | 0.00026 |
|  | K01195; beta-glucuronidase [EC:3.2.1.31] | 7.7E-05 | 6.47E-05 | 4.87E-05 | 4.64E-05 | 3.49E-05 | 3.18E-05 | 7.06E-05 | 7.14E-05 | 5.65E-05 | 4.84E-05 | 3.41E-05 | 3.24E-05 |
|  | K01180; endo-1,3(4)-beta-glucanase [EC:3.2.1.6] | 0 | 1.67E-08 | 1.49E-09 | 8.58E-10 | 0 | 0 | 0 | 1.72E-09 | 2.15E-09 | 5.17E-09 | 1.15E-09 | 0 |
|  | K01181; endo-1,4-beta-xylanase [EC:3.2.1.8] | 0.000122 | 0.000114 | 9.95E-05 | 0.000117 | 0.000157 | 0.000155 | 0.000133 | 0.000119 | 0.000117 | 0.000135 | 0.000156 | 0.000168 |
|  | K01190; beta-galactosidase [EC:3.2.1.23] | 0.0004 | 0.000363 | 0.000302 | 0.000317 | 0.000394 | 0.000389 | 0.000382 | 0.000381 | 0.00035 | 0.000358 | 0.000392 | 0.00041 |
|  | K01048; lysophospholipase [EC:3.1.1.5] | 0.000143 | 0.000182 | 0.000242 | 0.00023 | 0.000318 | 0.000371 | 0.000133 | 0.000163 | 0.000203 | 0.000241 | 0.000333 | 0.000384 |
|  | K01218; mannan endo-1,4-beta-mannosidase [EC:3.2.1.78] | 6.92E-05 | 6.16E-05 | 4.94E-05 | 5.14E-05 | 3.67E-05 | 2.65E-05 | 7.63E-05 | 6.55E-05 | 5.65E-05 | 5.17E-05 | 3.62E-05 | 2.52E-05 |
| **Lignin** | K03781; catalase [EC:1.11.1.6] | 0.000883 | 0.000875 | 0.000828 | 0.00083 | 0.000789 | 0.000753 | 0.000915 | 0.000875 | 0.00086 | 0.000846 | 0.000786 | 0.00075 |
|  | K03782; catalase-peroxidase [EC:1.11.1.21] | 0.000734 | 0.000669 | 0.000644 | 0.000671 | 0.000627 | 0.000604 | 0.000731 | 0.000714 | 0.000676 | 0.000655 | 0.000618 | 0.000593 |
|  | K00428; cytochrome c peroxidase [EC:1.11.1.5] | 0.000333 | 0.000298 | 0.000289 | 0.000325 | 0.000279 | 0.000268 | 0.000318 | 0.000324 | 0.000304 | 0.000292 | 0.000269 | 0.000256 |
|  | K00432; glutathione peroxidase [EC:1.11.1.9] | 0.000313 | 0.000289 | 0.000245 | 0.000258 | 0.000202 | 0.000164 | 0.000326 | 0.0003 | 0.000277 | 0.000254 | 0.000195 | 0.000163 |
|  | K00104; glycolate oxidase [EC:1.1.3.15] | 0.000449 | 0.000479 | 0.000527 | 0.000534 | 0.00055 | 0.000581 | 0.000456 | 0.000465 | 0.000496 | 0.00052 | 0.000553 | 0.000577 |
|  | K03862; vanillate monooxygenase [EC:1.14.13.82] | 0.000127 | 0.000146 | 0.000169 | 0.000177 | 0.00024 | 0.000232 | 0.000136 | 0.000135 | 0.000159 | 0.000177 | 0.000231 | 0.00022 |
|  | K03863; vanillate monooxygenase [EC:1.14.13.82] | 0.000152 | 0.000143 | 0.000135 | 0.000151 | 0.00014 | 0.000126 | 0.000161 | 0.000149 | 0.000146 | 0.000146 | 0.000132 | 0.000125 |

**Supplementary Table S9**

Impact of decomposition time and climate regime on the relative abundance of potential genes involved in wheat straw decomposition. The functional profiles of bacterial communities were predicted based on the 16S rRNA genes of retrieved bacterial taxa using Tax4Fun according to the KEGG Ortholog groups (KOs).

| Potential gene category | Time | | Climate | | test |
| --- | --- | --- | --- | --- | --- |
|  | χ2 | *p* | *Z* | *p* |  |
| Cellulose | 20.857 | 0.001 | -1.427 | 0.1537 | Friedman Test, Mann-Whitney U test |
| Hemicellulose | 31.943 | <0.001 | -1.146 | 0.251 | Friedman Test, Mann-Whitney U test |
| Lignin | 33.029 | <0.001 | -0.362 | 0.717 | Friedman Test, Mann-Whitney U test |

| **Order** | **Time** | | **Climate** | | **Statistical test** |
| --- | --- | --- | --- | --- | --- |
|  | ***F* / χ2** | ***p*** | ***F* / *Z*** | ***p*** |  |
| **Capnodiales** | 45.543 | <0.001 | -0.066 | 0.947 | Friedman Test, Mann-Whitney U test |
| **Pleosporales** | 20.457 | 0.001 | -0.428 | 0.668 | Friedman Test, Mann-Whitney U test |
| **Glomerellales** | 11.600 | 0.041 | -1.405 | 0.160 | Friedman Test, Mann-Whitney U test |
| **Hypocreales** | 6.081 | <0.001 | 3.655 | 0.092 | Repeated measures ANOVA |
| **Sordariales** | 47.152 | <0.001 | -1.145 | 0.252 | Friedman Test, Mann-Whitney U test |
| **Trichosphaeriales** | 2.442 | 0.785 | -0.453 | 0.650 | Friedman Test, Mann-Whitney U test |
| **Xylariales** | 23.746 | <0.001 | -0.244 | 0.806 | Friedman Test, Mann-Whitney U test |
| **Agaricales** | 20.263 | 0.001 | -0.259 | 0.795 | Friedman Test, Mann-Whitney U test |
| **Cantharellales** | 32.601 | <0.001 | -0.041 | 0.967 | Friedman Test, Mann-Whitney U test |

**Supplementary Table S10**

Impact of decomposition time and climate regime on the most abundant (relative sequence abundance > 1% of all fungal sequences) fungal orders on wheat straw.

**Supplementary Table S11**

Impact of decomposition time and climate regime on the top 20 most abundant (relative sequence abundance) fungal genera colonizing wheat straw.

| **Order** | **Time** | | **Climate** | | **Statistical test** |
| --- | --- | --- | --- | --- | --- |
|  | ***F* / χ2** | ***p*** | ***F* / *Z*** | ***p*** |  |
| *Mycosphaerella tassiana* (ASV_3) | 45.355 | < 0.001 | -1.535 | 0.124 | Friedman test, Mann-Whitney U test |
| *Mycosphaerella tassiana* (ASV_9) | 19.532 | < 0.001 | 3.827 | 0.086 | Repeated measures ANOVA |
| *Alternaria infectoria* (ASV_14) | 40.872 | < 0.001 | -1.182 | 0.237 | Repeated measures ANOVA |
| *Sistotrema* (ASV_4) | 24.282 | < 0.001 | 0 | 1 | Friedman test, Mann-Whitney U test |
| *Chaetomium globosum* (ASV_6) | 8.266 | 0.005 | 0.041 | 0.845 | Repeated measures ANOVA |
| *Chaetomium angustispirale* (ASV_8) | 11.095 | 0.008 | 0.869 | 0.379 | Repeated measures ANOVA |
| *Podospora* (ASV_10) | 21.093 | 0.001 | 1.145 | 0.316 | Repeated measures ANOVA |
| *Alternaria hordeicola* (ASV_5) | 19.532 | < 0.001 | 3.827 | 0.086 | Repeated measures ANOVA |
| *Fusarium poae* (ASV_44) | 25.273 | < 0.001 | -2.219 | 0.026 | Friedman test, Mann-Whitney U test |
| *Gibberella intricans* (ASV_38) | 16.743 | 0.005 | -0.275 | 0.783 | Friedman test, Mann-Whitney U test |
| *Gibberella pulicaris* (ASV_36) | 20.802 | 0.001 | -0.798 | 0.424 | Friedman test, Mann-Whitney U test |
| *Gibellulopsis nigrescens* (ASV_47) | 24.580 | < 0.001 | -0.192 | 0.847 | Friedman test, Mann-Whitney U test |
| *Stachybotrys chartarum* (ASV_13) | 44.362 | < 0.001 | -0.829 | 0.406 | Friedman test, Mann-Whitney U test |
| *Alternaria infectoria* (ASV_7) | 42.652 | < 0.001 | -1.177 | 0.239 | Friedman test, Mann-Whitney U test |
| *Preussia terricola* (ASV_22) | 38.659 | < 0.001 | 3.786 | 0.088 | Repeated measures ANOVA |
| *Preussia terricola* (ASV_25) | 31.623 | < 0.001 | -0.096 | 0.923 | Friedman test, Mann-Whitney U test |
| *Nigrospora oryzae* (ASV_61) | 24.762 | < 0.001 | -0.255 | 0.798 | Friedman test, Mann-Whitney U test |
| *Gibellulopsis nigrescens* (ASV_65) | 23.788 | < 0.001 | -0.170 | 0.864 | Friedman test, Mann-Whitney U test |
| *Sarocladium*  (ASV_16) | 37.029 | < 0.001 | -1.538 | 0.124 | Friedman test, Mann-Whitney U test |
| *Schizothecium inaequale* (ASV_21) | 38.889 | < 0.001 | -0.021 | 0.983 | Friedman test, Mann-Whitney U test |

**Supplementary Table S12**

Impact of decomposition time and climate regime on the relative abundance of potential potential fungal traits involved in wheat straw decomposition.

| Fungal trait | Time | | Climate | | test |
| --- | --- | --- | --- | --- | --- |
|  | *F* / χ2 | *p* | *F* / *Z* | *p* |  |
| Plant pathogens | 44.914 | <0.001 | -0.391 | 0.695 | Friedman Test, Mann-Whitney U test |
| Litter saprotrophs | 38.836 | <0.001 | -0.717 | 0.472 | Friedman Test, Mann-Whitney U test |
| Wood Saprotrophs | 40.828 | <0.001 | -0.938 | 0.348 | Friedman Test, Mann-Whitney U test |
| Other saprotrophs (soil /dung / unspecific) | 70.160 | <0.001 | 7.196 | 0.028 | Repeated measures ANOVA |

**Supplementary Table S13**

Spearman’s rank correlation between fungal ecological functions and enzymes activity as well as wheat straw mass.

| Function | Straw mass | | β_glucosidase | | N-acetyl-glucosaminidase | | Phosphatase | | Phenol oxidase | | Peroxidase | |
| --- | --- | --- | --- | --- | --- | --- | --- | --- | --- | --- | --- | --- |
|  | *r* | *p* | *r* | *p* | *r* | *p* | *r* | *p* | *r* | *p* | *r* | *p* |
| General saprotrophs | -0.87129 | 1.4E-19 | 0.74167 | 1.21E-11 | 0.62416 | 9.9E-08 | 0.37567 | 0.003098 | 0.09024 | 0.4929 | 0.52597 | 1.59E-05 |
| Litter saprotroph | -0.79579 | 3.01E-14 | 0.63565 | 4.85E-08 | 0.58188 | 1.08E-06 | 0.36815 | 0.003803 | -0.08698 | 0.50874 | 0.51485 | 2.57E-05 |
| Plant pathogen | 0.86615 | 4.05E-19 | -0.76338 | 1.32E-12 | -0.65818 | 1.1E-08 | -0.46657 | 0.000172 | -0.11776 | 0.3702 | -0.62567 | 9.03E-08 |
| Wood saprotroph | -0.63814 | 4.14E-08 | 0.66205 | 8.39E-09 | 0.63979 | 3.73E-08 | 0.29903 | 0.020292 | 0.045826 | 0.72808 | 0.5135 | 2.72E-05 |

**Supplementary Table S14**

Taxonomic composition of microbial interaction network during early phase (0–60 D) of wheat straw decomposition under ambient climate regime

| ID | No. module | Zi | Pi | Taxonomy | | | |
| --- | --- | --- | --- | --- | --- | --- | --- |
| ASVB_5 | 1 | -0.961 | 0 | Gammaproteobacteria | Enterobacterales | Erwiniaceae | Pantoea |
| ASVB_6 | 1 | -0.961 | 0 | Gammaproteobacteria | Enterobacterales | Erwiniaceae | Pantoea |
| ASVB_9 | 0 | -0.814 | 0 | Gammaproteobacteria | Burkholderiales | Oxalobacteraceae | Massilia |
| ASVB_4 | 3 | -1.268 | 0 | Gammaproteobacteria | Burkholderiales | Oxalobacteraceae | Massilia |
| ASVB_16 | 2 | -1.461 | 0 | Gammaproteobacteria | Pseudomonadales | Pseudomonadaceae | Pseudomonas |
| ASVB_31 | 1 | -0.961 | 0 | Gammaproteobacteria | Pseudomonadales | Pseudomonadaceae | Pseudomonas |
| ASVB_47 | 0 | -0.181 | 0 | Gammaproteobacteria | Enterobacterales | Erwiniaceae | Pantoea |
| ASVB_48 | 2 | 0.141 | 0.375 | Gammaproteobacteria | Enterobacterales | Erwiniaceae | Pantoea |
| ASVB_19 | 0 | -0.814 | 0 | Alphaproteobacteria | Sphingomonadales | Sphingomonadaceae | Sphingomonas |
| ASVB_50 | 1 | -0.961 | 0 | Gammaproteobacteria | Pseudomonadales | Pseudomonadaceae | Pseudomonas |
| ASVB_66 | 0 | -0.814 | 0 | Actinobacteria | Micrococcales | Microbacteriaceae | Rathayibacter |
| ASVB_64 | 0 | -0.814 | 0 | Actinobacteria | Micrococcales | Microbacteriaceae | Frigoribacterium |
| ASVB_174 | 0 | 0.452 | 0.56 | Gammaproteobacteria | Gammaproteobacteria | Gammaproteobacteria | Gammaproteobacteria |
| ASVB_26 | 1 | 2.335 | 0.617 | Gammaproteobacteria | Burkholderiales | Oxalobacteraceae | Massilia |
| ASVB_112 | 2 | 0.942 | 0 | Actinobacteria | Micrococcales | Microbacteriaceae | Frigoribacterium |
| ASVB_93 | 2 | 0.942 | 0.571 | Gammaproteobacteria | Pseudomonadales | Pseudomonadaceae | Pseudomonas |
| ASVB_149 | 0 | -0.814 | 0 | Gammaproteobacteria | Pseudomonadales | Pseudomonadaceae | Pseudomonas |
| ASVB_84 | 2 | 0.942 | 0 | Gammaproteobacteria | Enterobacterales | Erwiniaceae | Pantoea |
| ASVB_220 | 1 | 0.686 | 0.56 | Gammaproteobacteria | Enterobacterales | Erwiniaceae | Pantoea |
| ASVB_195 | 0 | 0.452 | 0 | Gammaproteobacteria | Enterobacterales | Erwiniaceae | Pantoea |
| ASVB_18 | 2 | -0.659 | 0.444 | Gammaproteobacteria | Xanthomonadales | Xanthomonadaceae | Stenotrophomonas |
| ASVB_205 | 3 | -1.268 | 0 | Gammaproteobacteria | Enterobacterales | Enterobacterales | Enterobacterales |
| ASVB_53 | 1 | -0.137 | 0.625 | Actinobacteria | Micrococcales | Microbacteriaceae | Curtobacterium |
| ASVB_49 | 2 | 0.141 | 0.375 | Alphaproteobacteria | Sphingomonadales | Sphingomonadaceae | Sphingomonas |
| ASVB_144 | 3 | 1.087 | 0.5 | Actinobacteria | Micrococcales | Microbacteriaceae | Frigoribacterium |
| ASVB_157 | 2 | -0.659 | 0 | Gammaproteobacteria | Enterobacterales | Erwiniaceae | Pantoea |
| ASVB_88 | 1 | -0.961 | 0.5 | Gammaproteobacteria | Pseudomonadales | Pseudomonadaceae | Pseudomonas |
| ASVB_37 | 0 | 1.72 | 0.66 | Gammaproteobacteria | Pseudomonadales | Pseudomonadaceae | Pseudomonas |
| ASVB_240 | 3 | -1.268 | 0 | Gammaproteobacteria | Pseudomonadales | Pseudomonadaceae | Pseudomonas |
| ASVB_42 | 2 | 0.942 | 0.444 | Alphaproteobacteria | Rhizobiales | Rhizobiaceae | Rhizobium |
| ASVB_81 | 1 | 0.686 | 0 | Actinobacteria | Micrococcales | Microbacteriaceae | Microbacteriaceae |
| ASVB_391 | 0 | -0.814 | 0 | Gammaproteobacteria | Pseudomonadales | Pseudomonadaceae | Pseudomonas |
| ASVB_372 | 2 | -0.659 | 0.444 | Gammaproteobacteria | Enterobacterales | Erwiniaceae | Erwiniaceae |
| ASVB_323 | 3 | 1.087 | 0.571 | Gammaproteobacteria | Pseudomonadales | Pseudomonadaceae | Pseudomonas |
| ASVB_20 | 3 | -0.483 | 0.444 | Alphaproteobacteria | Sphingomonadales | Sphingomonadaceae | Sphingomonas |
| ASVB_94 | 0 | -0.814 | 0 | Actinobacteria | Micrococcales | Microbacteriaceae | Pseudoclavibacter |
| ASVB_248 | 3 | -0.483 | 0.444 | Actinobacteria | Corynebacteriales | Nocardiaceae | Rhodococcus |
| ASVB_13 | 3 | 1.087 | 0 | Alphaproteobacteria | Caulobacterales | Caulobacteraceae | Brevundimonas |
| ASVB_431 | 3 | 0.302 | 0.611 | Gammaproteobacteria | Enterobacterales | Erwiniaceae | Pantoea |
| ASVB_172 | 3 | 0.302 | 0 | Alphaproteobacteria | Sphingomonadales | Sphingomonadaceae | Sphingomonas |
| ASVB_60 | 2 | 0.942 | 0.444 | Gammaproteobacteria | Pseudomonadales | Pseudomonadaceae | Pseudomonas |
| ASVB_419 | 1 | 0.686 | 0.693 | Bacilli | Paenibacillales | Paenibacillaceae | Paenibacillus |
| ASVB_646 | 1 | 0.686 | 0.375 | Gammaproteobacteria | Enterobacterales | Erwiniaceae | Pantoea |
| ASVB_820 | 2 | -0.659 | 0 | Actinobacteria | Micrococcales | Microbacteriaceae | Rathayibacter |
| ASVB_991 | 2 | 0.942 | 0.612 | Actinobacteria | Micrococcales | Microbacteriaceae | Microbacteriaceae |
| ASVF_47 | 3 | 1.872 | 0.66 | Sordariomycetes | Glomerellales | Plectosphaerellaceae | Gibellulopsis |
| ASVF_61 | 2 | 1.744 | 0.448 | Sordariomycetes | Trichosphaeriales | Trichosphaeriaceae | Nigrospora |
| ASVF_68 | 2 | -1.461 | 0 | Sordariomycetes | Xylariales | Xylariales | Xylariales |
| ASVF_65 | 2 | -1.461 | 0.5 | Sordariomycetes | Glomerellales | Plectosphaerellaceae | Gibellulopsis |
| ASVF_44 | 0 | 2.353 | 0.666 | Sordariomycetes | Hypocreales | Nectriaceae | Fusarium |
| ASVF_96 | 0 | 0.452 | 0.56 | Dothideomycetes | Pleosporales | Pleosporaceae | Alternaria |
| ASVF_118 | 0 | 0.452 | 0.687 | Dothideomycetes | Capnodiales | Mycosphaerellaceae | Zymoseptoria |
| ASVF_179 | 3 | -0.483 | 0.444 | Tremellomycetes | Filobasidiales | Filobasidiaceae | Filobasidium |
| ASVF_180 | 2 | -0.659 | 0.444 | Tremellomycetes | Tremellales | Bulleribasidiaceae | Vishniacozyma |
| ASVF_183 | 1 | -0.137 | 0.444 | Tremellomycetes | Tremellales | Bulleribasidiaceae | Vishniacozyma |
| ASVF_203 | 3 | -0.483 | 0.444 | Tremellomycetes | Filobasidiales | Filobasidiaceae | Filobasidium |

**Supplementary Table S15**

Taxonomic composition of microbial interaction network during early phase (0–60 D) of wheat straw decomposition under future climate regime

| ID | No. module | Zi | Pi | Taxonomy | | | |
| --- | --- | --- | --- | --- | --- | --- | --- |
| ASVB_5 | 5 | -1.801 | 0 | Gammaproteobacteria | Enterobacterales | Erwiniaceae | Pantoea |
| ASVB_6 | 3 | -0.861 | 0.666 | Gammaproteobacteria | Enterobacterales | Erwiniaceae | Pantoea |
| ASVB_2 | 4 | -1.078 | 0.5 | Gammaproteobacteria | Burkholderiales | Oxalobacteraceae | Massilia |
| ASVB_48 | 0 | 0.118 | 0.693 | Gammaproteobacteria | Enterobacterales | Erwiniaceae | Pantoea |
| ASVB_9 | 3 | -0.861 | 0.5 | Gammaproteobacteria | Burkholderiales | Oxalobacteraceae | Massilia |
| ASVB_43 | 0 | 0.118 | 0.693 | Gammaproteobacteria | Pseudomonadales | Pseudomonadaceae | Pseudomonas |
| ASVB_4 | 5 | -1.165 | 0.444 | Gammaproteobacteria | Burkholderiales | Oxalobacteraceae | Massilia |
| ASVB_7 | 2 | -1.08 | 0.5 | Gammaproteobacteria | Burkholderiales | Oxalobacteraceae | Massilia |
| ASVB_40 | 5 | -0.529 | 0.375 | Actinobacteria | Micrococcales | Sanguibacteraceae | Sanguibacter |
| ASVB_15 | 3 | -0.861 | 0.5 | Gammaproteobacteria | Burkholderiales | Oxalobacteraceae | Massilia |
| ASVB_47 | 1 | 0.384 | 0.56 | Gammaproteobacteria | Enterobacterales | Erwiniaceae | Pantoea |
| ASVB_22 | 4 | -0.196 | 0.625 | Gammaproteobacteria | Burkholderiales | Oxalobacteraceae | Massilia |
| ASVB_105 | 0 | 0.118 | 0 | Gammaproteobacteria | Enterobacterales | Erwiniaceae | Pantoea |
| ASVB_50 | 0 | -0.593 | 0.72 | Gammaproteobacteria | Pseudomonadales | Pseudomonadaceae | Pseudomonas |
| ASVB_165 | 1 | -0.256 | 0.625 | Gammaproteobacteria | Pseudomonadales | Pseudomonadaceae | Pseudomonas |
| ASVB_31 | 3 | -0.861 | 0 | Gammaproteobacteria | Pseudomonadales | Pseudomonadaceae | Pseudomonas |
| ASVB_16 | 1 | -0.256 | 0 | Gammaproteobacteria | Pseudomonadales | Pseudomonadaceae | Pseudomonas |
| ASVB_157 | 4 | 0.686 | 0 | Gammaproteobacteria | Enterobacterales | Erwiniaceae | Pantoea |
| ASVB_153 | 0 | 2.254 | 0.644 | Gammaproteobacteria | Enterobacterales | Erwiniaceae | Pantoea |
| ASVB_64 | 5 | 0.105 | 0.656 | Actinobacteria | Micrococcales | Microbacteriaceae | Frigoribacterium |
| ASVB_112 | 4 | -0.196 | 0.5 | Actinobacteria | Micrococcales | Microbacteriaceae | Frigoribacterium |
| ASVB_66 | 4 | -1.078 | 0 | Actinobacteria | Micrococcales | Microbacteriaceae | Rathayibacter |
| ASVB_18 | 4 | 2.451 | 0.641 | Gammaproteobacteria | Xanthomonadales | Xanthomonadaceae | Stenotrophomonas |
| ASVB_11 | 1 | 0.384 | 0.734 | Gammaproteobacteria | Pseudomonadales | Pseudomonadaceae | Pseudomonas |
| ASVB_149 | 2 | 0.925 | 0.612 | Gammaproteobacteria | Pseudomonadales | Pseudomonadaceae | Pseudomonas |
| ASVB_240 | 2 | -0.411 | 0.722 | Gammaproteobacteria | Pseudomonadales | Pseudomonadaceae | Pseudomonas |
| ASVB_220 | 5 | -0.529 | 0 | Gammaproteobacteria | Enterobacterales | Erwiniaceae | Pantoea |
| ASVB_228 | 3 | 0.984 | 0.5 | Gammaproteobacteria | Gammaproteobacteria | Gammaproteobacteria | Gammaproteobacteria |
| ASVB_88 | 1 | 1.024 | 0.666 | Gammaproteobacteria | Pseudomonadales | Pseudomonadaceae | Pseudomonas |
| ASVB_49 | 2 | 2.263 | 0.6 | Alphaproteobacteria | Sphingomonadales | Sphingomonadaceae | Sphingomonas |
| ASVB_144 | 3 | 2.215 | 0.737 | Actinobacteria | Micrococcales | Microbacteriaceae | Frigoribacterium |
| ASVB_60 | 5 | 0.741 | 0.68 | Gammaproteobacteria | Pseudomonadales | Pseudomonadaceae | Pseudomonas |
| ASVB_53 | 3 | 0.369 | 0.56 | Actinobacteria | Micrococcales | Microbacteriaceae | Curtobacterium |
| ASVB_37 | 4 | -0.196 | 0.72 | Gammaproteobacteria | Pseudomonadales | Pseudomonadaceae | Pseudomonas |
| ASVB_81 | 2 | 0.257 | 0.666 | Actinobacteria | Micrococcales | Microbacteriaceae | Microbacteriaceae |
| ASVB_118 | 0 | 0.118 | 0.79 | Actinobacteria | Micrococcales | Microbacteriaceae | Agreia |
| ASVB_42 | 5 | 1.377 | 0.666 | Alphaproteobacteria | Rhizobiales | Rhizobiaceae | Rhizobium |
| ASVB_13 | 2 | -0.411 | 0.625 | Alphaproteobacteria | Caulobacterales | Caulobacteraceae | Brevundimonas |
| ASVB_372 | 5 | -0.529 | 0.56 | Gammaproteobacteria | Enterobacterales | Erwiniaceae | Erwiniaceae |
| ASVB_248 | 1 | -0.896 | 0.5 | Actinobacteria | Corynebacteriales | Nocardiaceae | Rhodococcus |
| ASVB_20 | 2 | 0.257 | 0.56 | Alphaproteobacteria | Sphingomonadales | Sphingomonadaceae | Sphingomonas |
| ASVB_323 | 2 | -0.411 | 0.444 | Gammaproteobacteria | Pseudomonadales | Pseudomonadaceae | Pseudomonas |
| ASVB_94 | 4 | -0.196 | 0.625 | Actinobacteria | Micrococcales | Microbacteriaceae | Pseudoclavibacter |
| ASVB_516 | 0 | -0.593 | 0.625 | Gammaproteobacteria | Enterobacterales | Erwiniaceae | Erwiniaceae |
| ASVB_55 | 5 | 1.377 | 0.444 | Alphaproteobacteria | Rhizobiales | Rhizobiaceae | Rhizobium |
| ASVB_382 | 5 | -0.529 | 0.375 | Bacilli | Exiguobacterales | Exiguobacteraceae | Exiguobacterium |
| ASVB_172 | 2 | 1.594 | 0.775 | Alphaproteobacteria | Sphingomonadales | Sphingomonadaceae | Sphingomonas |
| ASVB_1192 | 5 | 1.377 | 0.58 | Gammaproteobacteria | Burkholderiales | Oxalobacteraceae | Massilia |
| ASVF_3 | 3 | -0.861 | 0 | Dothideomycetes | Cladosporiaceae | Capnodiales | Mycosphaerella |
| ASVF_9 | 2 | -1.08 | 0 | Dothideomycetes | Cladosporiaceae | Capnodiales | Mycosphaerella |
| ASVF_14 | 2 | -1.08 | 0 | Dothideomycetes | Pleosporales | Pleosporaceae | Alternaria |
| ASVF_44 | 1 | -0.896 | 0.666 | Sordariomycetes | Hypocreales | Nectriaceae | Fusarium |
| ASVF_36 | 1 | 2.304 | 0.674 | Sordariomycetes | Hypocreales | Nectriaceae | Gibberella |
| ASVF_65 | 1 | -0.896 | 0.5 | Sordariomycetes | Glomerellales | Plectosphaerellaceae | Gibellulopsis |
| ASVF_68 | 1 | -0.896 | 0.5 | Sordariomycetes | Xylariales | Xylariales | Xylariales |
| ASVF_96 | 0 | -1.305 | 0 | Dothideomycetes | Pleosporales | Pleosporaceae | Alternaria |
| ASVF_47 | 0 | -0.593 | 0.444 | Sordariomycetes | Glomerellales | Plectosphaerellaceae | Gibellulopsis |
| ASVF_61 | 4 | -0.196 | 0.72 | Sordariomycetes | Trichosphaeriales | Trichosphaeriaceae | Nigrospora |
| ASVF_139 | 0 | 0.118 | 0.48 | Dothideomycetes | Pleosporales | Didymellaceae | Neoascochyta |
| ASVF_169 | 5 | 0.105 | 0.691 | Sordariomycetes | Hypocreales | Hypocreales | Hypocreales |
| ASVF_118 | 0 | 1.542 | 0.277 | Dothideomycetes | Capnodiales | Mycosphaerellaceae | Zymoseptoria |
| ASVF_183 | 3 | 0.369 | 0 | Tremellomycetes | Tremellales | Bulleribasidiaceae | Vishniacozyma |
| ASVF_180 | 0 | -1.305 | 0 | Tremellomycetes | Tremellales | Bulleribasidiaceae | Vishniacozyma |
| ASVF_179 | 2 | -0.411 | 0.444 | Tremellomycetes | Filobasidiales | Filobasidiaceae | Filobasidium |
| ASVF_203 | 3 | 0.369 | 0.56 | Tremellomycetes | Filobasidiales | Filobasidiaceae | Filobasidium |
| ASVF_256 | 2 | -0.411 | 0.444 | Dothideomycetes | Pleosporales | Pleosporaceae | Stemphylium |

**Supplementary Table S16**

Taxonomic composition of microbial interaction network during the later phase (120–420 D) of wheat straw decomposition under ambient climate regime

| ID | No. module | Zi | Pi | Taxonomy | | | |
| --- | --- | --- | --- | --- | --- | --- | --- |
| ASVB_1 | 4 | -1.006 | 0 | Actinobacteria | Micrococcales | Promicromonosporaceae | Promicromonospora |
| ASVB_3 | 3 | -0.273 | 0.277 | Actinobacteria | Micrococcales | Promicromonosporaceae | Promicromonospora |
| ASVB_4 | 4 | -0.75 | 0.32 | Gammaproteobacteria | Burkholderiales | Oxalobacteraceae | Massilia |
| ASVB_8 | 3 | -0.8 | 0.56 | Actinobacteria | Micrococcales | Promicromonosporaceae | Promicromonospora |
| ASVB_2 | 0 | -0.665 | 0.75 | Gammaproteobacteria | Burkholderiales | Oxalobacteraceae | Massilia |
| ASVB_7 | 5 | -1.02 | 0.5 | Gammaproteobacteria | Burkholderiales | Oxalobacteraceae | Massilia |
| ASVB_14 | 0 | -0.665 | 0.75 | Gammaproteobacteria | Burkholderiales | Comamonadaceae | Variovorax |
| ASVB_13 | 3 | -0.8 | 0.375 | Alphaproteobacteria | Caulobacterales | Caulobacteraceae | Brevundimonas |
| ASVB_12 | 4 | -0.75 | 0.571 | Bacteroidia | Flavobacteriales | Weeksellaceae | Chryseobacterium |
| ASVB_9 | 0 | -0.147 | 0.74 | Gammaproteobacteria | Burkholderiales | Oxalobacteraceae | Massilia |
| ASVB_16 | 3 | -0.8 | 0.375 | Gammaproteobacteria | Pseudomonadales | Pseudomonadaceae | Pseudomonas |
| ASVB_17 | 3 | -1.326 | 0.5 | Alphaproteobacteria | Rhizobiales | Rhizobiaceae | Rhizobium |
| ASVB_20 | 3 | -0.8 | 0.56 | Alphaproteobacteria | Sphingomonadales | Sphingomonadaceae | Sphingomonas |
| ASVB_10 | 5 | -0.581 | 0 | Bacteroidia | Flavobacteriales | Flavobacteriaceae | Flavobacterium |
| ASVB_11 | 2 | -1.876 | 0.666 | Gammaproteobacteria | Pseudomonadales | Pseudomonadaceae | Pseudomonas |
| ASVB_18 | 1 | -0.979 | 0.666 | Gammaproteobacteria | Xanthomonadales | Xanthomonadaceae | Stenotrophomonas |
| ASVB_15 | 5 | 1.174 | 0.702 | Gammaproteobacteria | Burkholderiales | Oxalobacteraceae | Massilia |
| ASVB_24 | 1 | -0.979 | 0 | Alphaproteobacteria | Caulobacterales | Caulobacteraceae | Brevundimonas |
| ASVB_23 | 1 | -0.979 | 0.777 | Alphaproteobacteria | Rhizobiales | Rhizobiaceae | Rhizobium |
| ASVB_19 | 3 | -1.063 | 0.722 | Alphaproteobacteria | Sphingomonadales | Sphingomonadaceae | Sphingomonas |
| ASVB_26 | 4 | -0.75 | 0.593 | Gammaproteobacteria | Burkholderiales | Oxalobacteraceae | Massilia |
| ASVB_29 | 3 | -1.063 | 0.625 | Alphaproteobacteria | Rhizobiales | Rhizobiaceae | Rhizobium |
| ASVB_30 | 3 | -0.273 | 0.408 | Actinobacteria | Pseudonocardiales | Pseudonocardiaceae | Lechevalieria |
| ASVB_22 | 3 | -0.273 | 0.531 | Gammaproteobacteria | Burkholderiales | Oxalobacteraceae | Massilia |
| ASVB_5 | 2 | -1.482 | 0.64 | Gammaproteobacteria | Enterobacterales | Erwiniaceae | Pantoea |
| ASVB_34 | 1 | 0.178 | 0.777 | Bacteroidia | Flavobacteriales | Flavobacteriaceae | Flavobacteriaceae |
| ASVB_27 | 2 | -0.695 | 0.656 | Bacteroidia | Flavobacteriales | Flavobacteriaceae | Flavobacterium |
| ASVB_32 | 4 | -1.263 | 0.625 | Actinobacteria | Micrococcales | Microbacteriaceae | Galbitalea |
| ASVB_39 | 5 | -1.458 | 0.75 | Alphaproteobacteria | Sphingomonadales | Sphingomonadaceae | Sphingomonadaceae |
| ASVB_36 | 3 | -0.8 | 0.693 | Gammaproteobacteria | Burkholderiales | Oxalobacteraceae | Oxalobacteraceae |
| ASVB_35 | 3 | -1.063 | 0.625 | Actinobacteria | Micromonosporales | Micromonosporaceae | Actinoplanes |
| ASVB_42 | 3 | -1.063 | 0.444 | Alphaproteobacteria | Rhizobiales | Rhizobiaceae | Rhizobium |
| ASVB_33 | 4 | -0.236 | 0.493 | Bacteroidia | Flavobacteriales | Flavobacteriaceae | Flavobacterium |
| ASVB_28 | 3 | 0.253 | 0.734 | Bacteroidia | Flavobacteriales | Flavobacteriaceae | Flavobacterium |
| ASVB_31 | 4 | -1.006 | 0.56 | Gammaproteobacteria | Pseudomonadales | Pseudomonadaceae | Pseudomonas |
| ASVB_21 | 1 | 0.178 | 0.781 | Bacteroidia | Flavobacteriales | Flavobacteriaceae | Flavobacterium |
| ASVB_6 | 4 | -1.263 | 0 | Gammaproteobacteria | Enterobacterales | Erwiniaceae | Pantoea |
| ASVB_41 | 3 | -0.536 | 0.5 | Alphaproteobacteria | Rhizobiales | Rhizobiaceae | Phyllobacterium |
| ASVB_25 | 4 | -0.75 | 0.5 | Bacteroidia | Flavobacteriales | Flavobacteriaceae | Flavobacterium |
| ASVB_57 | 3 | 2.097 | 0.512 | Actinobacteria | Pseudonocardiales | Pseudonocardiaceae | Umezawaea |
| ASVB_55 | 2 | -1.089 | 0.693 | Alphaproteobacteria | Rhizobiales | Rhizobiaceae | Rhizobium |
| ASVB_54 | 2 | 1.272 | 0.632 | Actinobacteria | Corynebacteriales | Mycobacteriaceae | Mycobacterium |
| ASVB_79 | 4 | 0.532 | 0.725 | Actinobacteria | Corynebacteriales | Mycobacteriaceae | Mycobacterium |
| ASVB_37 | 3 | -1.326 | 0.5 | Gammaproteobacteria | Pseudomonadales | Pseudomonadaceae | Pseudomonas |
| ASVB_44 | 0 | -0.665 | 0.75 | Alphaproteobacteria | Caulobacterales | Caulobacteraceae | Brevundimonas |
| ASVB_49 | 0 | 0.369 | 0.776 | Alphaproteobacteria | Sphingomonadales | Sphingomonadaceae | Sphingomonas |
| ASVB_45 | 2 | -1.089 | 0.375 | Bacteroidia | Flavobacteriales | Flavobacteriaceae | Flavobacterium |
| ASVB_53 | 0 | -0.665 | 0.666 | Actinobacteria | Micrococcales | Microbacteriaceae | Curtobacterium |
| ASVB_56 | 4 | -1.006 | 0.693 | Gammaproteobacteria | Burkholderiales | Comamonadaceae | Pseudorhodoferax |
| ASVB_61 | 0 | -0.147 | 0.72 | Gammaproteobacteria | Burkholderiales | Alcaligenaceae | Alcaligenaceae |
| ASVB_68 | 3 | 2.36 | 0.536 | Bacteroidia | Flavobacteriales | Flavobacteriaceae | Flavobacterium |
| ASVB_90 | 2 | -0.695 | 0.656 | Bacteroidia | Flavobacteriales | Flavobacteriaceae | Flavobacterium |
| ASVB_74 | 3 | -0.009 | 0.406 | Alphaproteobacteria | Rhizobiales | Rhizobiaceae | Rhizobium |
| ASVB_91 | 3 | -0.536 | 0.743 | Gammaproteobacteria | Burkholderiales | Oxalobacteraceae | Massilia |
| ASVB_62 | 4 | -1.263 | 0.72 | Bacteroidia | Flavobacteriales | Flavobacteriaceae | Flavobacterium |
| ASVB_114 | 2 | -0.302 | 0.694 | Bacteroidia | Flavobacteriales | Flavobacteriaceae | Flavobacterium |
| ASVB_46 | 3 | -0.536 | 0.571 | Bacteroidia | Flavobacteriales | Flavobacteriaceae | Flavobacterium |
| ASVB_67 | 3 | -0.009 | 0.75 | Gammaproteobacteria | Burkholderiales | Oxalobacteraceae | Duganella |
| ASVB_77 | 5 | -1.02 | 0.625 | Actinobacteria | Micrococcales | Microbacteriaceae | Herbiconiux |
| ASVB_69 | 5 | -1.02 | 0.5 | Alphaproteobacteria | Rhizobiales | Rhizobiaceae | Rhizobium |
| ASVB_98 | 1 | -0.979 | 0.666 | Actinobacteria | Micromonosporales | Micromonosporaceae | Catenuloplanes |
| ASVB_60 | 4 | -0.75 | 0.76 | Gammaproteobacteria | Pseudomonadales | Pseudomonadaceae | Pseudomonas |
| ASVB_72 | 5 | -0.142 | 0.66 | Bacteroidia | Flavobacteriales | Flavobacteriaceae | Flavobacterium |
| ASVB_80 | 3 | -0.009 | 0.56 | Gammaproteobacteria | Burkholderiales | Comamonadaceae | Comamonadaceae |
| ASVB_94 | 4 | 0.019 | 0.218 | Actinobacteria | Micrococcales | Microbacteriaceae | Pseudoclavibacter |
| ASVB_123 | 0 | 0.369 | 0.666 | Gammaproteobacteria | Burkholderiales | Methylophilaceae | Methylotenera |
| ASVB_81 | 4 | 0.789 | 0.588 | Actinobacteria | Micrococcales | Microbacteriaceae | Microbacteriaceae |
| ASVB_63 | 4 | -0.75 | 0.656 | Gammaproteobacteria | Burkholderiales | Comamonadaceae | Pseudorhodoferax |
| ASVB_65 | 3 | 1.043 | 0.648 | Actinobacteria | Micromonosporales | Micromonosporaceae | Actinoplanes |
| ASVB_82 | 3 | 1.043 | 0.581 | Actinobacteria | Streptomycetales | Streptomycetaceae | Streptomyces |
| ASVB_40 | 5 | -1.02 | 0.444 | Actinobacteria | Micrococcales | Sanguibacteraceae | Sanguibacter |
| ASVB_76 | 5 | -0.581 | 0.56 | Actinobacteria | Micrococcales | Microbacteriaceae | Salinibacterium |
| ASVB_87 | 3 | -0.536 | 0.593 | Alphaproteobacteria | Rhizobiales | Beijerinckiaceae | Bosea |
| ASVB_116 | 5 | -1.02 | 0.781 | Gammaproteobacteria | Burkholderiales | Alcaligenaceae | Verticiella |
| ASVB_59 | 4 | 1.816 | 0.633 | Actinobacteria | Micrococcales | Microbacteriaceae | Conyzicola |
| ASVB_70 | 3 | -0.8 | 0.718 | Alphaproteobacteria | Rhizobiales | Rhizobiaceae | Rhizobium |
| ASVB_117 | 0 | -0.147 | 0.64 | Bacteroidia | Flavobacteriales | Flavobacteriaceae | Flavobacterium |
| ASVB_111 | 2 | -0.695 | 0.625 | Alphaproteobacteria | Sphingomonadales | Sphingomonadaceae | Sphingobium |
| ASVB_109 | 2 | -0.695 | 0.5 | Alphaproteobacteria | Sphingomonadales | Sphingomonadaceae | Sphingomonas |
| ASVB_96 | 3 | -0.009 | 0.768 | Bacteroidia | Flavobacteriales | Weeksellaceae | Chryseobacterium |
| ASVB_75 | 0 | -0.665 | 0.5 | Actinobacteria | Micrococcales | Microbacteriaceae | Microbacterium |
| ASVB_64 | 4 | -0.75 | 0.656 | Actinobacteria | Micrococcales | Microbacteriaceae | Frigoribacterium |
| ASVB_101 | 0 | -0.665 | 0.72 | Alphaproteobacteria | Sphingomonadales | Sphingomonadaceae | Sphingomonas |
| ASVB_73 | 5 | 1.174 | 0.726 | Gammaproteobacteria | Burkholderiales | Oxalobacteraceae | Massilia |
| ASVB_142 | 5 | -1.458 | 0 | Bacteroidia | Flavobacteriales | Flavobacteriaceae | Flavobacterium |
| ASVB_110 | 3 | -0.8 | 0.56 | Alphaproteobacteria | Sphingomonadales | Sphingomonadaceae | Sphingomonas |
| ASVB_95 | 3 | -0.009 | 0.666 | Bacteroidia | Flavobacteriales | Flavobacteriaceae | Flavobacterium |
| ASVB_50 | 5 | 0.735 | 0.698 | Gammaproteobacteria | Pseudomonadales | Pseudomonadaceae | Pseudomonas |
| ASVB_88 | 5 | 2.49 | 0.707 | Gammaproteobacteria | Pseudomonadales | Pseudomonadaceae | Pseudomonas |
| ASVB_83 | 4 | 0.019 | 0.46 | Gammaproteobacteria | Burkholderiales | Comamonadaceae | Variovorax |
| ASVB_151 | 0 | 3.995 | 0.791 | Actinobacteria | Propionibacteriales | Nocardioidaceae | Kribbella |
| ASVB_97 | 5 | 0.296 | 0.68 | Gammaproteobacteria | Burkholderiales | Comamonadaceae | Acidovorax |
| ASVB_113 | 2 | -0.302 | 0.793 | Alphaproteobacteria | Rhizobiales | Rhizobiaceae | Rhizobium |
| ASVB_66 | 3 | 0.253 | 0.74 | Actinobacteria | Micrococcales | Microbacteriaceae | Rathayibacter |
| ASVB_122 | 2 | 0.091 | 0.674 | Alphaproteobacteria | Rhizobiales | Rhizobiaceae | Rhizobium |
| ASVB_143 | 3 | 1.307 | 0.626 | Alphaproteobacteria | Rhizobiales | Devosiaceae | Devosia |
| ASVB_177 | 4 | 0.276 | 0.735 | Gammaproteobacteria | Burkholderiales | Oxalobacteraceae | Massilia |
| ASVB_118 | 5 | 0.296 | 0.722 | Actinobacteria | Micrococcales | Microbacteriaceae | Agreia |
| ASVB_106 | 3 | 1.57 | 0.656 | Alphaproteobacteria | Sphingomonadales | Sphingomonadaceae | Sphingomonas |
| ASVB_145 | 2 | 0.091 | 0.652 | Gammaproteobacteria | Xanthomonadales | Xanthomonadaceae | Luteimonas |
| ASVB_141 | 4 | -0.75 | 0.66 | Alphaproteobacteria | Caulobacterales | Caulobacteraceae | Brevundimonas |
| ASVB_135 | 4 | 0.276 | 0.34 | Alphaproteobacteria | Rhizobiales | Xanthobacteraceae | Rhodopseudomonas |
| ASVB_148 | 4 | 1.046 | 0.62 | Actinobacteria | Micrococcales | Microbacteriaceae | Microbacteriaceae |
| ASVB_71 | 5 | -0.581 | 0.765 | Alphaproteobacteria | Rhizobiales | Rhizobiaceae | Pseudorhizobium |
| ASVB_132 | 4 | 1.046 | 0.532 | Alphaproteobacteria | Rhizobiales | Xanthobacteraceae | Ancylobacter |
| ASVB_146 | 2 | 0.485 | 0.684 | Gammaproteobacteria | Pseudomonadales | Pseudomonadaceae | Pseudomonas |
| ASVB_103 | 4 | -1.006 | 0.612 | Alphaproteobacteria | Rhizobiales | Rhizobiaceae | Shinella |
| ASVB_134 | 5 | 0.296 | 0.408 | Alphaproteobacteria | Rhizobiales | Devosiaceae | Devosia |
| ASVB_159 | 2 | -0.695 | 0.656 | Actinobacteria | Corynebacteriales | Mycobacteriaceae | Mycobacterium |
| ASVB_173 | 2 | -0.302 | 0.66 | Gammaproteobacteria | Burkholderiales | Oxalobacteraceae | Massilia |
| ASVB_139 | 4 | -1.263 | 0 | Actinobacteria | Propionibacteriales | Nocardioidaceae | Nocardioides |
| ASVB_154 | 3 | -0.009 | 0.721 | Alphaproteobacteria | Caulobacterales | Caulobacteraceae | Brevundimonas |
| ASVB_100 | 2 | 0.091 | 0.686 | Bacteroidia | Flavobacteriales | Weeksellaceae | Chryseobacterium |
| ASVB_124 | 0 | 0.369 | 0.716 | Actinobacteria | Corynebacteriales | Mycobacteriaceae | Mycobacterium |
| ASVB_108 | 0 | -0.147 | 0.666 | Gammaproteobacteria | Burkholderiales | Oxalobacteraceae | Massilia |
| ASVB_89 | 2 | 0.485 | 0.627 | Gammaproteobacteria | Burkholderiales | Oxalobacteraceae | Herminiimonas |
| ASVB_93 | 4 | 1.302 | 0.7 | Gammaproteobacteria | Pseudomonadales | Pseudomonadaceae | Pseudomonas |
| ASVB_176 | 4 | 0.019 | 0.683 | Actinobacteria | Propionibacteriales | Nocardioidaceae | Aeromicrobium |
| ASVB_86 | 4 | -0.75 | 0.444 | Gammaproteobacteria | Pseudomonadales | Pseudomonadaceae | Pseudomonas |
| ASVB_130 | 4 | -0.493 | 0.71 | Gammaproteobacteria | Burkholderiales | Oxalobacteraceae | Massilia |
| ASVB_92 | 2 | 1.272 | 0.609 | Gammaproteobacteria | Burkholderiales | Comamonadaceae | Polaromonas |
| ASVB_178 | 4 | -0.236 | 0.718 | Alphaproteobacteria | Caulobacterales | Caulobacteraceae | Caulobacter |
| ASVB_254 | 4 | -0.236 | 0.518 | Gammaproteobacteria | Burkholderiales | Alcaligenaceae | Pigmentiphaga |
| ASVB_137 | 2 | -0.695 | 0.757 | Alphaproteobacteria | Rhizobiales | Xanthobacteraceae | Rhodopseudomonas |
| ASVB_194 | 3 | -0.273 | 0.617 | Alphaproteobacteria | Rhizobiales | Beijerinckiaceae | Methylobacterium-Methylorubrum |
| ASVB_166 | 3 | 2.36 | 0.365 | Alphaproteobacteria | Rhizobiales | Rhizobiaceae | Rhizobium |
| ASVB_224 | 5 | -0.581 | 0.611 | Alphaproteobacteria | Rhizobiales | Rhizobiaceae | Rhizobium |
| ASVB_115 | 4 | -0.493 | 0.617 | Alphaproteobacteria | Rhizobiales | Devosiaceae | Devosia |
| ASVB_201 | 0 | -0.147 | 0.775 | Alphaproteobacteria | Rhizobiales | Devosiaceae | Devosia |
| ASVB_226 | 4 | 0.532 | 0.69 | Alphaproteobacteria | Sphingomonadales | Sphingomonadaceae | Sphingomonas |
| ASVB_246 | 4 | 0.276 | 0.631 | Actinobacteria | Micrococcales | Microbacteriaceae | Microbacterium |
| ASVB_126 | 3 | 2.097 | 0.646 | Actinobacteria | Streptomycetales | Streptomycetaceae | Streptomyces |
| ASVB_193 | 2 | -1.089 | 0.375 | Alphaproteobacteria | Rhizobiales | Rhizobiaceae | Rhizobium |
| ASVB_291 | 3 | 0.516 | 0.602 | Alphaproteobacteria | Rhizobiales | Rhizobiaceae | Aureimonas |
| ASVB_198 | 5 | -0.142 | 0.71 | Alphaproteobacteria | Rhizobiales | Devosiaceae | Devosia |
| ASVB_192 | 4 | 3.099 | 0.354 | Alphaproteobacteria | Caulobacterales | Caulobacteraceae | uncultured |
| ASVB_236 | 4 | 0.276 | 0.698 | Gammaproteobacteria | Xanthomonadales | Xanthomonadaceae | Stenotrophomonas |
| ASVB_209 | 3 | 0.516 | 0.631 | Alphaproteobacteria | Sphingomonadales | Sphingomonadaceae | Novosphingobium |
| ASVB_187 | 2 | -1.089 | 0.48 | Alphaproteobacteria | Rhizobiales | Rhizobiaceae | Rhizobium |
| ASVB_200 | 4 | 1.559 | 0.708 | Actinobacteria | Micrococcales | Cellulomonadaceae | Cellulomonas |
| ASVB_275 | 3 | -0.009 | 0.244 | Alphaproteobacteria | Rhizobiales | Beijerinckiaceae | Methylobacterium-Methylorubrum |
| ASVB_127 | 2 | -0.302 | 0.448 | Alphaproteobacteria | Rhizobiales | Rhizobiaceae | Rhizobium |
| ASVB_288 | 0 | -0.147 | 0.5 | Alphaproteobacteria | Acetobacterales | Acetobacteraceae | Roseomonas |
| ASVB_189 | 2 | 0.485 | 0.784 | Gammaproteobacteria | Burkholderiales | Oxalobacteraceae | Massilia |
| ASVB_185 | 4 | -0.493 | 0.65 | Alphaproteobacteria | Rhizobiales | Devosiaceae | Devosia |
| ASVB_232 | 0 | -0.147 | 0.79 | Alphaproteobacteria | Caulobacterales | Caulobacteraceae | Caulobacter |
| ASVB_262 | 5 | -0.142 | 0.32 | Gammaproteobacteria | Burkholderiales | Methylophilaceae | Methylotenera |
| ASVB_208 | 2 | 0.878 | 0.64 | Gammaproteobacteria | Burkholderiales | Comamonadaceae | Comamonadaceae |
| ASVB_324 | 5 | 0.296 | 0.79 | Gammaproteobacteria | Oceanospirillales | Pseudohongiellaceae | BIyi10 |
| ASVB_369 | 1 | 2.494 | 0.763 | Alphaproteobacteria | Sphingomonadales | Sphingomonadaceae | Novosphingobium |
| ASVB_302 | 4 | 0.532 | 0.5 | Alphaproteobacteria | Caulobacterales | Caulobacteraceae | Phenylobacterium |
| ASVB_211 | 5 | -0.581 | 0.693 | Actinobacteria | Propionibacteriales | Nocardioidaceae | Nocardioides |
| ASVB_221 | 2 | 0.485 | 0.345 | Alphaproteobacteria | Sphingomonadales | Sphingomonadaceae | Sphingomonas |
| ASVB_355 | 5 | -0.581 | 0.693 | Alphaproteobacteria | Rhizobiales | Beijerinckiaceae | Methylobacterium-Methylorubrum |
| ASVB_317 | 1 | 0.178 | 0.722 | Actinobacteria | Micrococcales | Microbacteriaceae | Agromyces |
| ASVB_338 | 3 | 0.516 | 0.513 | Gammaproteobacteria | Burkholderiales | Alcaligenaceae | Verticiella |
| ASVB_247 | 5 | 0.296 | 0.62 | Alphaproteobacteria | Rhizobiales | Rhizobiaceae | Rhizobium |
| ASVB_172 | 1 | 0.178 | 0.781 | Alphaproteobacteria | Sphingomonadales | Sphingomonadaceae | Sphingomonas |
| ASVB_269 | 5 | -0.581 | 0.781 | Gammaproteobacteria | Oceanospirillales | Pseudohongiellaceae | BIyi10 |
| ASVB_287 | 5 | -1.02 | 0.722 | Alphaproteobacteria | Sphingomonadales | Sphingomonadaceae | Sphingomonas |
| ASVB_218 | 5 | -0.142 | 0.571 | Actinobacteria | Micrococcales | Cellulomonadaceae | Cellulomonas |
| ASVB_304 | 4 | 2.329 | 0.594 | Alphaproteobacteria | Caulobacterales | Caulobacteraceae | Brevundimonas |
| ASVB_352 | 5 | 0.296 | 0.698 | Gammaproteobacteria | Burkholderiales | Alcaligenaceae | Pigmentiphaga |
| ASVB_163 | 2 | -0.302 | 0.745 | Bacteroidia | Flavobacteriales | Flavobacteriaceae | Flavobacterium |
| ASVB_364 | 3 | 0.78 | 0.617 | Alphaproteobacteria | Rhizobiales | Devosiaceae | Devosia |
| ASVB_336 | 2 | 0.878 | 0.579 | Alphaproteobacteria | Caulobacterales | Caulobacteraceae | Brevundimonas |
| ASVB_484 | 5 | 2.49 | 0.524 | Alphaproteobacteria | Rhizobiales | Devosiaceae | Devosia |
| ASVB_244 | 4 | 1.046 | 0.357 | Abditibacteria | Abditibacteriales | Abditibacteriaceae | Abditibacterium |
| ASVB_284 | 5 | 1.613 | 0.739 | Actinobacteria | Corynebacteriales | Mycobacteriaceae | Mycobacterium |
| ASVB_495 | 3 | -0.8 | 0.716 | Actinobacteria | Micrococcales | Microbacteriaceae | Microbacterium |
| ASVB_403 | 5 | 0.296 | 0.677 | Actinobacteria | Micrococcales | Microbacteriaceae | Labedella |
| ASVB_416 | 4 | 1.559 | 0.32 | Alphaproteobacteria | Rhizobiales | Xanthobacteraceae | Pseudolabrys |
| ASVB_573 | 2 | 0.878 | 0.739 | Alphaproteobacteria | Sphingomonadales | Sphingomonadaceae | Sphingomonas |
| ASVF_4 | 2 | 0.878 | 0.579 | Agaricomycetes | Cantharellales | Cantharellales_fam_Incertae_sedis | Sistotrema |
| ASVF_2 | 3 | -0.536 | 0.5 | Sordariomycetes | Hypocreales | Hypocreales | Hypocreales |
| ASVF_1 | 4 | -0.493 | 0 | Dothideomycetes | Capnodiales | Capnodiales | Capnodiales |
| ASVF_8 | 3 | -0.8 | 0.375 | Sordariomycetes | Sordariales | Chaetomiaceae | Chaetomium |
| ASVF_6 | 3 | -0.8 | 0.375 | Sordariomycetes | Sordariales | Chaetomiaceae | Chaetomium |
| ASVF_5 | 4 | -0.75 | 0.571 | Dothideomycetes | Pleosporales | Pleosporaceae | Alternaria |
| ASVF_10 | 3 | -0.8 | 0 | Sordariomycetes | Sordariales | Lasiosphaeriaceae | Podospora |
| ASVF_7 | 2 | -1.089 | 0.56 | Dothideomycetes | Pleosporales | Pleosporaceae | Alternaria |
| ASVF_13 | 4 | -1.006 | 0.48 | Sordariomycetes | Hypocreales | Stachybotryaceae | Stachybotrys |
| ASVF_21 | 2 | -0.302 | 0.789 | Sordariomycetes | Sordariales | Lasiosphaeriaceae | Schizothecium |
| ASVF_18 | 2 | 0.091 | 0.518 | Dothideomycetes | Capnodiales | Cladosporiaceae | Cladosporium |
| ASVF_22 | 3 | -0.8 | 0.666 | Dothideomycetes | Pleosporales | Sporormiaceae | Preussia |
| ASVF_29 | 2 | 0.091 | 0.406 | Sordariomycetes | Glomerellales | Plectosphaerellaceae | Gibellulopsis |
| ASVF_19 | 2 | 0.091 | 0.493 | Dothideomycetes | Pleosporales | Pleosporaceae | Alternaria |
| ASVF_37 | 5 | -1.02 | 0.625 | Sordariomycetes | Glomerellales | Plectosphaerellaceae | Gibellulopsis |
| ASVF_35 | 4 | -0.236 | 0.615 | Sordariomycetes | Sordariales | Lasiosphaeriaceae | Lasiosphaeriaceae |
| ASVF_12 | 3 | 0.78 | 0.659 | Sordariomycetes | Hypocreales | Nectriaceae | Gibberella |
| ASVF_45 | 3 | 2.624 | 0.66 | Sordariomycetes | Sordariales | Lasiosphaeriaceae | Apodus |
| ASVF_24 | 1 | 0.178 | 0.444 | Sordariomycetes | Trichosphaeriales | Trichosphaeriaceae | Nigrospora |
| ASVF_23 | 1 | 1.336 | 0.716 | Tremellomycetes | Holtermanniales | Holtermanniales_fam_Incertae_  sedis | Holtermanniella |
| ASVF_48 | 2 | 2.846 | 0.635 | Sordariomycetes | Hypocreales | Hypocreales_fam_Incertae_sedis | Sarocladium |
| ASVF_30 | 3 | -0.273 | 0.698 | Sordariomycetes | Sordariales | Lasiosphaeriaceae | Podospora |
| ASVF_16 | 4 | 0.019 | 0.663 | Sordariomycetes | Hypocreales | Hypocreales_fam_Incertae_sedis | Sarocladium |
| ASVF_54 | 5 | 0.296 | 0.744 | Sordariomycetes | Sordariales | Lasiosphaeriaceae | Podospora |
| ASVF_33 | 2 | 0.878 | 0.77 | Sordariomycetes | Hypocreales | Hypocreales | Hypocreales |
| ASVF_31 | 0 | -0.665 | 0.722 | Dothideomycetes | Pleosporales | Sporormiaceae | Preussia |
| ASVF_39 | 0 | -0.665 | 0.775 | Sordariomycetes | Microascales | Microascales | Microascales |
| ASVF_15 | 2 | 0.091 | 0.595 | Sordariomycetes | Xylariales | Xylariales | Xylariales |
| ASVF_57 | 5 | -0.142 | 0.7 | Sordariomycetes | Sordariales | Lasiosphaeriaceae | Cladorrhinum |
| ASVF_43 | 4 | 1.559 | 0.601 | Sordariomycetes | Sordariales | Lasiosphaeriaceae | Lasiosphaeriaceae |
| ASVF_74 | 4 | 0.019 | 0.65 | Sordariomycetes | Hypocreales | Hypocreales | Hypocreales |
| ASVF_46 | 4 | 1.046 | 0.685 | Sordariomycetes | Sordariales | Sordariales | Sordariales |
| ASVF_80 | 4 | -0.493 | 0.71 | Sordariomycetes | Hypocreales | Hypocreales | Hypocreales |
| ASVF_26 | 1 | -0.979 | 0.72 | Tremellomycetes | Filobasidiales | Filobasidiaceae | Filobasidium |
| ASVF_59 | 2 | 0.485 | 0.561 | Leotiomycetes | Thelebolales | Pseudeurotiaceae | Pseudogymnoascus |
| ASVF_28 | 5 | 1.613 | 0.768 | Tremellomycetes | Filobasidiales | Filobasidiaceae | Filobasidium |
| ASVF_127 | 0 | 0.887 | 0.763 | Eurotiomycetes | Chaetothyriales | Herpotrichiellaceae | Exophiala |
| ASVF_11 | 3 | 0.253 | 0.545 | Sordariomycetes | Hypocreales | Nectriaceae | Gibberella |
| ASVF_70 | 0 | 0.369 | 0.765 | Tremellomycetes | Holtermanniales | Holtermanniales_fam_Incertae_  sedis | Holtermanniella |
| ASVF_73 | 5 | 1.174 | 0.673 | Tremellomycetes | Tremellales | Bulleribasidiaceae | Vishniacozyma |
| ASVF_110 | 2 | -0.695 | 0.571 | Dothideomycetes | Pleosporales | Phaeosphaeriaceae | Phaeosphaeria |
| ASVF_102 | 2 | -0.695 | 0.656 | Sordariomycetes | Sordariales | Lasiosphaeriaceae | Cladorrhinum |
| ASVF_64 | 2 | 0.091 | 0.663 | Rhizophlyctidomycetes | Rhizophlyctidales | Rhizophlyctidaceae | Rhizophlyctis |
| ASVF_163 | 1 | 0.178 | 0.791 | Dothideomycetes | Pleosporales | Didymosphaeriaceae | Didymosphaeriaceae |
| ASVF_122 | 3 | 0.253 | 0.48 | Sordariomycetes | Sordariales | Sordariales | Sordariales |
| ASVF_133 | 2 | 3.239 | 0.676 | Sordariomycetes | Hypocreales | Hypocreales_fam_Incertae_sedis | Acremonium |

**Supplementary Table S17**

Taxonomic composition of microbial interaction network during the later phase (120–420 D) of wheat straw decomposition under future climate regime

| ID | No. module | Zi | Pi | Taxonomy | | | |
| --- | --- | --- | --- | --- | --- | --- | --- |
| ASVB_1 | 2 | -0.428 | 0.5 | Actinobacteria | Micrococcales | Promicromonosporaceae | Promicromonospora |
| ASVB_10 | 1 | -1.007 | 0.64 | Bacteroidia | Flavobacteriales | Flavobacteriaceae | Flavobacterium |
| ASVB_3 | 2 | -0.737 | 0 | Actinobacteria | Micrococcales | Promicromonosporaceae | Promicromonospora |
| ASVB_2 | 3 | -0.289 | 0.406 | Gammaproteobacteria | Burkholderiales | Oxalobacteraceae | Massilia |
| ASVB_11 | 3 | -0.289 | 0.244 | Gammaproteobacteria | Pseudomonadales | Pseudomonadaceae | Pseudomonas |
| ASVB_8 | 3 | -0.289 | 0.493 | Actinobacteria | Micrococcales | Promicromonosporaceae | Promicromonospora |
| ASVB_9 | 3 | -0.476 | 0.277 | Gammaproteobacteria | Burkholderiales | Oxalobacteraceae | Massilia |
| ASVB_14 | 0 | -1.134 | 0.666 | Gammaproteobacteria | Burkholderiales | Comamonadaceae | Variovorax |
| ASVB_6 | 3 | -0.848 | 0.74 | Gammaproteobacteria | Enterobacterales | Erwiniaceae | Pantoea |
| ASVB_21 | 1 | -0.76 | 0.56 | Bacteroidia | Flavobacteriales | Flavobacteriaceae | Flavobacterium |
| ASVB_5 | 2 | -1.355 | 0.666 | Gammaproteobacteria | Enterobacterales | Erwiniaceae | Pantoea |
| ASVB_13 | 1 | -1.007 | 0.72 | Alphaproteobacteria | Caulobacterales | Caulobacteraceae | Brevundimonas |
| ASVB_4 | 2 | 0.189 | 0.56 | Gammaproteobacteria | Burkholderiales | Oxalobacteraceae | Massilia |
| ASVB_25 | 2 | -0.737 | 0.56 | Bacteroidia | Flavobacteriales | Flavobacteriaceae | Flavobacterium |
| ASVB_18 | 3 | -0.848 | 0.375 | Gammaproteobacteria | Xanthomonadales | Xanthomonadaceae | Stenotrophomonas |
| ASVB_7 | 1 | -0.264 | 0.277 | Gammaproteobacteria | Burkholderiales | Oxalobacteraceae | Massilia |
| ASVB_12 | 0 | -0.387 | 0.727 | Bacteroidia | Flavobacteriales | Weeksellaceae | Chryseobacterium |
| ASVB_19 | 3 | 0.269 | 0.641 | Alphaproteobacteria | Sphingomonadales | Sphingomonadaceae | Sphingomonas |
| ASVB_17 | 2 | -1.046 | 0 | Alphaproteobacteria | Rhizobiales | Rhizobiaceae | Rhizobium |
| ASVB_23 | 3 | -0.848 | 0.48 | Alphaproteobacteria | Rhizobiales | Rhizobiaceae | Rhizobium |
| ASVB_24 | 2 | -0.428 | 0.444 | Alphaproteobacteria | Caulobacterales | Caulobacteraceae | Brevundimonas |
| ASVB_20 | 0 | -0.761 | 0 | Alphaproteobacteria | Sphingomonadales | Sphingomonadaceae | Sphingomonas |
| ASVB_27 | 1 | -0.264 | 0.448 | Bacteroidia | Flavobacteriales | Flavobacteriaceae | Flavobacterium |
| ASVB_28 | 1 | -0.76 | 0.74 | Bacteroidia | Flavobacteriales | Flavobacteriaceae | Flavobacterium |
| ASVB_30 | 2 | -0.428 | 0.32 | Actinobacteria | Pseudonocardiales | Pseudonocardiaceae | Lechevalieria |
| ASVB_32 | 2 | -1.046 | 0.444 | Actinobacteria | Micrococcales | Microbacteriaceae | Galbitalea |
| ASVB_46 | 0 | -0.761 | 0.72 | Bacteroidia | Flavobacteriales | Flavobacteriaceae | Flavobacterium |
| ASVB_37 | 3 | -1.035 | 0.444 | Gammaproteobacteria | Pseudomonadales | Pseudomonadaceae | Pseudomonas |
| ASVB_41 | 1 | -1.255 | 0.5 | Alphaproteobacteria | Rhizobiales | Rhizobiaceae | Phyllobacterium |
| ASVB_35 | 2 | -0.737 | 0.611 | Actinobacteria | Micromonosporales | Micromonosporaceae | Actinoplanes |
| ASVB_39 | 1 | -1.255 | 0 | Alphaproteobacteria | Sphingomonadales | Sphingomonadaceae | Sphingomonadaceae |
| ASVB_33 | 1 | -0.76 | 0.375 | Bacteroidia | Flavobacteriales | Flavobacteriaceae | Flavobacterium |
| ASVB_44 | 3 | -0.848 | 0 | Alphaproteobacteria | Caulobacterales | Caulobacteraceae | Brevundimonas |
| ASVB_56 | 1 | -1.255 | 0.5 | Gammaproteobacteria | Burkholderiales | Comamonadaceae | Pseudorhodoferax |
| ASVB_52 | 1 | -0.017 | 0.54 | Bacteroidia | Flavobacteriales | Flavobacteriaceae | Flavobacterium |
| ASVB_45 | 1 | -0.264 | 0.448 | Bacteroidia | Flavobacteriales | Flavobacteriaceae | Flavobacterium |
| ASVB_29 | 3 | -1.035 | 0.444 | Alphaproteobacteria | Rhizobiales | Rhizobiaceae | Rhizobium |
| ASVB_36 | 3 | -0.289 | 0.406 | Gammaproteobacteria | Burkholderiales | Oxalobacteraceae | Oxalobacteraceae |
| ASVB_57 | 3 | 3.623 | 0.415 | Actinobacteria | Pseudonocardiales | Pseudonocardiaceae | Umezawaea |
| ASVB_16 | 1 | 0.23 | 0.528 | Gammaproteobacteria | Pseudomonadales | Pseudomonadaceae | Pseudomonas |
| ASVB_54 | 2 | -0.737 | 0.56 | Actinobacteria | Corynebacteriales | Mycobacteriaceae | Mycobacterium |
| ASVB_26 | 3 | 0.082 | 0.623 | Gammaproteobacteria | Burkholderiales | Oxalobacteraceae | Massilia |
| ASVB_63 | 2 | -1.046 | 0 | Gammaproteobacteria | Burkholderiales | Comamonadaceae | Pseudorhodoferax |
| ASVB_71 | 0 | -1.134 | 0.666 | Alphaproteobacteria | Rhizobiales | Rhizobiaceae | Pseudorhizobium |
| ASVB_38 | 0 | 0.359 | 0.71 | Bacteroidia | Flavobacteriales | Flavobacteriaceae | Flavobacterium |
| ASVB_42 | 1 | -0.264 | 0.617 | Alphaproteobacteria | Rhizobiales | Rhizobiaceae | Rhizobium |
| ASVB_34 | 0 | 0.359 | 0.448 | Bacteroidia | Flavobacteriales | Flavobacteriaceae | Flavobacteriaceae |
| ASVB_70 | 2 | -0.737 | 0.375 | Alphaproteobacteria | Rhizobiales | Rhizobiaceae | Rhizobium |
| ASVB_89 | 0 | -0.387 | 0.666 | Gammaproteobacteria | Burkholderiales | Oxalobacteraceae | Herminiimonas |
| ASVB_121 | 1 | 0.23 | 0.603 | Alphaproteobacteria | Rhizobiales | Rhizobiaceae | Phyllobacterium |
| ASVB_86 | 3 | -0.662 | 0.571 | Gammaproteobacteria | Pseudomonadales | Pseudomonadaceae | Pseudomonas |
| ASVB_107 | 1 | -0.76 | 0.693 | Bacteroidia | Flavobacteriales | Weeksellaceae | Chryseobacterium |
| ASVB_53 | 0 | -0.014 | 0.32 | Actinobacteria | Micrococcales | Microbacteriaceae | Curtobacterium |
| ASVB_61 | 3 | -0.662 | 0.666 | Gammaproteobacteria | Burkholderiales | Alcaligenaceae | Alcaligenaceae |
| ASVB_65 | 1 | 0.725 | 0.69 | Actinobacteria | Micromonosporales | Micromonosporaceae | Actinoplanes |
| ASVB_97 | 2 | -0.428 | 0.444 | Gammaproteobacteria | Burkholderiales | Comamonadaceae | Acidovorax |
| ASVB_115 | 0 | -1.134 | 0.64 | Alphaproteobacteria | Rhizobiales | Devosiaceae | Devosia |
| ASVB_79 | 1 | -0.264 | 0.708 | Actinobacteria | Corynebacteriales | Mycobacteriaceae | Mycobacterium |
| ASVB_55 | 3 | -0.662 | 0.694 | Alphaproteobacteria | Rhizobiales | Rhizobiaceae | Rhizobium |
| ASVB_83 | 3 | -0.662 | 0.571 | Gammaproteobacteria | Burkholderiales | Comamonadaceae | Variovorax |
| ASVB_31 | 2 | -0.737 | 0.48 | Gammaproteobacteria | Pseudomonadales | Pseudomonadaceae | Pseudomonas |
| ASVB_68 | 3 | 0.269 | 0.675 | Bacteroidia | Flavobacteriales | Flavobacteriaceae | Flavobacterium |
| ASVB_69 | 0 | -0.761 | 0.444 | Alphaproteobacteria | Rhizobiales | Rhizobiaceae | Rhizobium |
| ASVB_72 | 1 | -0.264 | 0.567 | Bacteroidia | Flavobacteriales | Flavobacteriaceae | Flavobacterium |
| ASVB_80 | 2 | 0.189 | 0.58 | Gammaproteobacteria | Burkholderiales | Comamonadaceae | Comamonadaceae |
| ASVB_60 | 0 | -0.761 | 0.611 | Gammaproteobacteria | Pseudomonadales | Pseudomonadaceae | Pseudomonas |
| ASVB_62 | 2 | 0.497 | 0.648 | Bacteroidia | Flavobacteriales | Flavobacteriaceae | Flavobacterium |
| ASVB_67 | 3 | 0.828 | 0.493 | Gammaproteobacteria | Burkholderiales | Oxalobacteraceae | Duganella |
| ASVB_82 | 1 | 1.964 | 0.65 | Actinobacteria | Streptomycetales | Streptomycetaceae | Streptomyces |
| ASVB_77 | 1 | -1.007 | 0.625 | Actinobacteria | Micrococcales | Microbacteriaceae | Herbiconiux |
| ASVB_127 | 0 | -1.134 | 0.5 | Alphaproteobacteria | Rhizobiales | Rhizobiaceae | Rhizobium |
| ASVB_50 | 2 | -1.046 | 0.64 | Gammaproteobacteria | Pseudomonadales | Pseudomonadaceae | Pseudomonas |
| ASVB_92 | 3 | -0.848 | 0.653 | Gammaproteobacteria | Burkholderiales | Comamonadaceae | Polaromonas |
| ASVB_103 | 1 | -0.512 | 0.32 | Alphaproteobacteria | Rhizobiales | Rhizobiaceae | Shinella |
| ASVB_75 | 0 | -1.134 | 0.666 | Actinobacteria | Micrococcales | Microbacteriaceae | Microbacterium |
| ASVB_136 | 3 | 1.946 | 0.607 | Bacteroidia | Flavobacteriales | Flavobacteriaceae | Flavobacterium |
| ASVB_51 | 0 | 1.106 | 0.671 | Bacteroidia | Flavobacteriales | Flavobacteriaceae | Flavobacterium |
| ASVB_106 | 1 | -0.76 | 0.687 | Alphaproteobacteria | Sphingomonadales | Sphingomonadaceae | Sphingomonas |
| ASVB_74 | 0 | -0.761 | 0.625 | Alphaproteobacteria | Rhizobiales | Rhizobiaceae | Rhizobium |
| ASVB_113 | 1 | -0.512 | 0.628 | Alphaproteobacteria | Rhizobiales | Rhizobiaceae | Rhizobium |
| ASVB_76 | 2 | -1.355 | 0 | Actinobacteria | Micrococcales | Microbacteriaceae | Salinibacterium |
| ASVB_87 | 0 | -0.387 | 0.74 | Alphaproteobacteria | Rhizobiales | Beijerinckiaceae | Bosea |
| ASVB_163 | 2 | 0.497 | 0.545 | Bacteroidia | Flavobacteriales | Flavobacteriaceae | Flavobacterium |
| ASVB_124 | 0 | -0.014 | 0.571 | Actinobacteria | Corynebacteriales | Mycobacteriaceae | Mycobacterium |
| ASVB_111 | 0 | -0.387 | 0.693 | Alphaproteobacteria | Sphingomonadales | Sphingomonadaceae | Sphingobium |
| ASVB_108 | 3 | -0.289 | 0.652 | Gammaproteobacteria | Burkholderiales | Oxalobacteraceae | Massilia |
| ASVB_101 | 2 | -0.428 | 0.727 | Alphaproteobacteria | Sphingomonadales | Sphingomonadaceae | Sphingomonas |
| ASVB_137 | 2 | -0.428 | 0.736 | Alphaproteobacteria | Rhizobiales | Xanthobacteraceae | Rhodopseudomonas |
| ASVB_132 | 1 | 0.973 | 0.642 | Alphaproteobacteria | Rhizobiales | Xanthobacteraceae | Ancylobacter |
| ASVB_102 | 0 | 1.479 | 0.715 | Gammaproteobacteria | Pseudomonadales | Pseudomonadaceae | Pseudomonas |
| ASVB_140 | 2 | -0.737 | 0.653 | Bacteroidia | Flavobacteriales | Flavobacteriaceae | Flavobacterium |
| ASVB_88 | 2 | 1.115 | 0.729 | Gammaproteobacteria | Pseudomonadales | Pseudomonadaceae | Pseudomonas |
| ASVB_139 | 1 | -0.76 | 0.375 | Actinobacteria | Propionibacteriales | Nocardioidaceae | Nocardioides |
| ASVB_40 | 1 | -0.264 | 0.677 | Actinobacteria | Micrococcales | Sanguibacteraceae | Sanguibacter |
| ASVB_110 | 3 | -0.848 | 0.666 | Alphaproteobacteria | Sphingomonadales | Sphingomonadaceae | Sphingomonas |
| ASVB_147 | 1 | 3.202 | 0.302 | Actinobacteria | Micrococcales | Promicromonosporaceae | Promicromonospora |
| ASVB_154 | 1 | -0.76 | 0.375 | Alphaproteobacteria | Caulobacterales | Caulobacteraceae | Brevundimonas |
| ASVB_143 | 1 | 0.725 | 0.461 | Alphaproteobacteria | Rhizobiales | Devosiaceae | Devosia |
| ASVB_141 | 1 | 0.23 | 0.639 | Alphaproteobacteria | Caulobacterales | Caulobacteraceae | Brevundimonas |
| ASVB_98 | 3 | 0.455 | 0.699 | Actinobacteria | Micromonosporales | Micromonosporaceae | Catenuloplanes |
| ASVB_120 | 0 | -0.014 | 0.5 | Gammaproteobacteria | Burkholderiales | Comamonadaceae | Acidovorax |
| ASVB_109 | 0 | -0.014 | 0.7 | Alphaproteobacteria | Sphingomonadales | Sphingomonadaceae | Sphingomonas |
| ASVB_117 | 0 | -1.134 | 0.625 | Bacteroidia | Flavobacteriales | Flavobacteriaceae | Flavobacterium |
| ASVB_135 | 3 | -0.848 | 0.611 | Alphaproteobacteria | Rhizobiales | Xanthobacteraceae | Rhodopseudomonas |
| ASVB_166 | 1 | -0.017 | 0.674 | Alphaproteobacteria | Rhizobiales | Rhizobiaceae | Rhizobium |
| ASVB_263 | 0 | 0.732 | 0.683 | Gammaproteobacteria | Burkholderiales | Methylophilaceae | Methylophilus |
| ASVB_191 | 1 | -0.76 | 0.716 | Actinobacteria | Micrococcales | Microbacteriaceae | Galbitalea |
| ASVB_126 | 1 | 0.23 | 0.671 | Actinobacteria | Streptomycetales | Streptomycetaceae | Streptomyces |
| ASVB_148 | 1 | 0.23 | 0.675 | Actinobacteria | Micrococcales | Microbacteriaceae | Microbacteriaceae |
| ASVB_130 | 3 | -0.662 | 0.656 | Gammaproteobacteria | Burkholderiales | Oxalobacteraceae | Massilia |
| ASVB_116 | 2 | 0.189 | 0.611 | Gammaproteobacteria | Burkholderiales | Alcaligenaceae | Verticiella |
| ASVB_123 | 3 | 1.2 | 0.563 | Gammaproteobacteria | Burkholderiales | Methylophilaceae | Methylotenera |
| ASVB_194 | 1 | -0.76 | 0.56 | Alphaproteobacteria | Rhizobiales | Beijerinckiaceae | Methylobacterium-Methylorubrum |
| ASVB_134 | 2 | -0.119 | 0.704 | Alphaproteobacteria | Rhizobiales | Devosiaceae | Devosia |
| ASVB_186 | 3 | -1.035 | 0.444 | Alphaproteobacteria | Caulobacterales | Caulobacteraceae | Brevundimonas |
| ASVB_159 | 0 | -0.014 | 0.691 | Actinobacteria | Corynebacteriales | Mycobacteriaceae | Mycobacterium |
| ASVB_219 | 1 | 0.23 | 0.698 | Bacteroidia | Flavobacteriales | Flavobacteriaceae | Flavobacterium |
| ASVB_188 | 0 | -0.014 | 0.71 | Alphaproteobacteria | Sphingomonadales | Sphingomonadaceae | Altererythrobacter |
| ASVB_211 | 2 | 0.189 | 0.406 | Actinobacteria | Propionibacteriales | Nocardioidaceae | Nocardioides |
| ASVB_199 | 3 | -0.103 | 0.556 | Alphaproteobacteria | Rhizobiales | Devosiaceae | Devosia |
| ASVB_231 | 2 | 0.497 | 0.671 | Actinobacteria | Micrococcales | Microbacteriaceae | Lysinimonas |
| ASVB_232 | 0 | -0.761 | 0 | Alphaproteobacteria | Caulobacterales | Caulobacteraceae | Caulobacter |
| ASVB_253 | 0 | -0.761 | 0.625 | Gammaproteobacteria | Burkholderiales | Methylophilaceae | Methylotenera |
| ASVB_212 | 2 | 1.733 | 0.654 | Gammaproteobacteria | Burkholderiales | Comamonadaceae | Rhizobacter |
| ASVB_156 | 2 | 0.806 | 0.571 | Bacteroidia | Flavobacteriales | Flavobacteriaceae | Flavobacterium |
| ASVB_185 | 0 | -0.014 | 0.7 | Alphaproteobacteria | Rhizobiales | Devosiaceae | Devosia |
| ASVB_192 | 1 | 0.23 | 0.734 | Alphaproteobacteria | Caulobacterales | Caulobacteraceae | uncultured |
| ASVB_251 | 0 | -0.387 | 0.612 | Gammaproteobacteria | Burkholderiales | Alcaligenaceae | Achromobacter |
| ASVB_221 | 3 | 0.082 | 0.513 | Alphaproteobacteria | Sphingomonadales | Sphingomonadaceae | Sphingomonas |
| ASVB_138 | 0 | 1.479 | 0.733 | Gammaproteobacteria | Xanthomonadales | Xanthomonadaceae | Stenotrophomonas |
| ASVB_218 | 3 | 0.641 | 0.476 | Actinobacteria | Micrococcales | Cellulomonadaceae | Cellulomonas |
| ASVB_244 | 0 | 0.359 | 0.704 | Abditibacteria | Abditibacteriales | Abditibacteriaceae | Abditibacterium |
| ASVB_277 | 0 | 0.732 | 0.625 | Gammaproteobacteria | Burkholderiales | Oxalobacteraceae | Oxalicibacterium |
| ASVB_207 | 2 | 0.189 | 0.683 | Gammaproteobacteria | Burkholderiales | Oxalobacteraceae | Massilia |
| ASVB_267 | 2 | 0.497 | 0.603 | Gammaproteobacteria | Burkholderiales | Comamonadaceae | Hydrogenophaga |
| ASVB_215 | 2 | 0.189 | 0.628 | Gammaproteobacteria | Burkholderiales | Oxalobacteraceae | Massilia |
| ASVB_196 | 1 | 1.468 | 0.642 | Actinobacteria | Corynebacteriales | Mycobacteriaceae | Mycobacterium |
| ASVB_237 | 3 | -1.035 | 0.666 | Gammaproteobacteria | Xanthomonadales | Xanthomonadaceae | Pseudoxanthomonas |
| ASVB_316 | 0 | 2.226 | 0.711 | Alphaproteobacteria | Rhizobiales | Rhizobiaceae | Rhizobium |
| ASVB_118 | 1 | -0.017 | 0.711 | Actinobacteria | Micrococcales | Microbacteriaceae | Agreia |
| ASVB_47 | 2 | 1.115 | 0.53 | Gammaproteobacteria | Enterobacterales | Erwiniaceae | Pantoea |
| ASVB_172 | 0 | 1.106 | 0.631 | Alphaproteobacteria | Sphingomonadales | Sphingomonadaceae | Sphingomonas |
| ASVB_178 | 3 | 1.014 | 0.597 | Alphaproteobacteria | Caulobacterales | Caulobacteraceae | Caulobacter |
| ASVB_187 | 3 | -0.476 | 0.628 | Alphaproteobacteria | Rhizobiales | Rhizobiaceae | Rhizobium |
| ASVB_225 | 3 | 1.387 | 0.6 | Bacteroidia | Flavobacteriales | Flavobacteriaceae | Flavobacterium |
| ASVB_293 | 0 | -1.134 | 0.72 | Gammaproteobacteria | Pseudomonadales | Pseudomonadaceae | Pseudomonas |
| ASVB_269 | 2 | -0.428 | 0.708 | Gammaproteobacteria | Oceanospirillales | Pseudohongiellaceae | BIyi10 |
| ASVB_200 | 1 | -0.512 | 0.656 | Actinobacteria | Micrococcales | Cellulomonadaceae | Cellulomonas |
| ASVB_282 | 2 | 1.424 | 0.707 | Alphaproteobacteria | Rhizobiales | Devosiaceae | Devosia |
| ASVB_304 | 2 | 0.189 | 0.693 | Alphaproteobacteria | Caulobacterales | Caulobacteraceae | Brevundimonas |
| ASVB_295 | 2 | 2.042 | 0.615 | Alphaproteobacteria | Sphingomonadales | Sphingomonadaceae | Sphingomonas |
| ASVB_319 | 2 | 0.497 | 0.663 | Alphaproteobacteria | Rhizobiales | Devosiaceae | Devosia |
| ASVB_176 | 0 | 0.732 | 0.734 | Actinobacteria | Propionibacteriales | Nocardioidaceae | Aeromicrobium |
| ASVB_183 | 1 | -0.264 | 0.612 | Bacteroidia | Flavobacteriales | Flavobacteriaceae | Flavobacterium |
| ASVB_198 | 2 | -1.046 | 0.444 | Alphaproteobacteria | Rhizobiales | Devosiaceae | Devosia |
| ASVB_255 | 0 | -0.761 | 0.444 | Gammaproteobacteria | Cellvibrionales | Cellvibrionaceae | Cellvibrio |
| ASVB_256 | 0 | 0.359 | 0.677 | Actinobacteria | Propionibacteriales | Nocardioidaceae | Nocardioides |
| ASVB_209 | 0 | -0.014 | 0.656 | Alphaproteobacteria | Sphingomonadales | Sphingomonadaceae | Novosphingobium |
| ASVB_317 | 1 | -0.017 | 0.711 | Actinobacteria | Micrococcales | Microbacteriaceae | Agromyces |
| ASVB_284 | 1 | -0.017 | 0.518 | Actinobacteria | Corynebacteriales | Mycobacteriaceae | Mycobacterium |
| ASVB_393 | 3 | 1.759 | 0.652 | Alphaproteobacteria | Sphingomonadales | Sphingomonadaceae | Altererythrobacter |
| ASVB_262 | 1 | -0.017 | 0.704 | Gammaproteobacteria | Burkholderiales | Methylophilaceae | Methylotenera |
| ASVB_239 | 1 | 0.725 | 0.314 | Gammaproteobacteria | Burkholderiales | Methylophilaceae | Methylotenera |
| ASVB_173 | 3 | 1.946 | 0.561 | Gammaproteobacteria | Burkholderiales | Oxalobacteraceae | Massilia |
| ASVB_324 | 3 | 0.269 | 0.67 | Gammaproteobacteria | Oceanospirillales | Pseudohongiellaceae | BIyi10 |
| ASVB_416 | 3 | -0.662 | 0.625 | Alphaproteobacteria | Rhizobiales | Xanthobacteraceae | Pseudolabrys |
| ASVB_342 | 3 | 0.641 | 0.639 | Bdellovibrionia | Bacteriovoracales | Bacteriovoracaceae | Peredibacter |
| ASVB_306 | 3 | -0.103 | 0.556 | Alphaproteobacteria | Sphingomonadales | Sphingomonadaceae | Sphingomonas |
| ASVB_404 | 0 | 2.6 | 0.742 | Alphaproteobacteria | Rhizobiales | Devosiaceae | Devosia |
| ASVB_351 | 1 | 0.478 | 0.513 | Alphaproteobacteria | Rhizobiales | Rhizobiaceae | Rhizobiaceae |
| ASVB_517 | 2 | 0.497 | 0.741 | Actinobacteria | Micromonosporales | Micromonosporaceae | Actinoplanes |
| ASVB_457 | 3 | 1.014 | 0.726 | Alphaproteobacteria | Sphingomonadales | Sphingomonadaceae | Sphingomonas |
| ASVB_302 | 1 | 3.202 | 0.551 | Alphaproteobacteria | Caulobacterales | Caulobacteraceae | Phenylobacterium |
| ASVB_495 | 3 | 1.946 | 0.488 | Actinobacteria | Micrococcales | Microbacteriaceae | Microbacterium |
| ASVF_2 | 3 | -0.848 | 0.375 | Sordariomycetes | Hypocreales | Hypocreales | Hypocreales |
| ASVF_1 | 1 | -1.007 | 0 | Dothideomycetes | Capnodiales | Capnodiales | Capnodiales |
| ASVF_10 | 0 | -1.134 | 0 | Sordariomycetes | Sordariales | Lasiosphaeriaceae | Podospora |
| ASVF_6 | 2 | -1.046 | 0.625 | Sordariomycetes | Sordariales | Chaetomiaceae | Chaetomium |
| ASVF_8 | 2 | -0.737 | 0.375 | Sordariomycetes | Sordariales | Chaetomiaceae | Chaetomium |
| ASVF_4 | 2 | 1.424 | 0.655 | Agaricomycetes | Cantharellales | Cantharellales_fam_Incertae_sedis | Sistotrema |
| ASVF_13 | 0 | -1.134 | 0 | Sordariomycetes | Hypocreales | Stachybotryaceae | Stachybotrys |
| ASVF_22 | 0 | -1.134 | 0.72 | Dothideomycetes | Pleosporales | Sporormiaceae | Preussia |
| ASVF_16 | 1 | -0.76 | 0.611 | Sordariomycetes | Hypocreales | Hypocreales_fam_Incertae_sedis | Sarocladium |
| ASVF_7 | 3 | 1.573 | 0.581 | Dothideomycetes | Pleosporales | Pleosporaceae | Alternaria |
| ASVF_31 | 3 | -0.476 | 0.531 | Dothideomycetes | Pleosporales | Sporormiaceae | Preussia |
| ASVF_20 | 3 | -0.848 | 0.375 | Dothideomycetes | Pleosporales | Phaeosphaeriaceae | Phaeosphaeriaceae |
| ASVF_5 | 3 | -0.476 | 0.644 | Dothideomycetes | Pleosporales | Pleosporaceae | Alternaria |
| ASVF_19 | 2 | -0.119 | 0.531 | Dothideomycetes | Pleosporales | Pleosporaceae | Alternaria |
| ASVF_45 | 0 | 2.226 | 0.732 | Sordariomycetes | Sordariales | Lasiosphaeriaceae | Apodus |
| ASVF_30 | 2 | -1.046 | 0.444 | Sordariomycetes | Sordariales | Lasiosphaeriaceae | Podospora |
| ASVF_21 | 3 | -0.848 | 0.611 | Sordariomycetes | Sordariales | Lasiosphaeriaceae | Schizothecium |
| ASVF_18 | 1 | -0.512 | 0.489 | Dothideomycetes | Capnodiales | Cladosporiaceae | Cladosporium |
| ASVF_46 | 3 | -0.662 | 0.5 | Sordariomycetes | Sordariales | Sordariales | Sordariales |
| ASVF_39 | 0 | -1.134 | 0.722 | Sordariomycetes | Microascales | Microascales | Microascales |
| ASVF_43 | 0 | -0.387 | 0.74 | Sordariomycetes | Sordariales | Lasiosphaeriaceae | Lasiosphaeriaceae |
| ASVF_35 | 3 | 0.455 | 0.665 | Sordariomycetes | Sordariales | Lasiosphaeriaceae | Lasiosphaeriaceae |
| ASVF_69 | 1 | 0.23 | 0.684 | Sordariomycetes | Sordariales | Lasiosphaeriaceae | Podospora |
| ASVF_72 | 1 | 0.725 | 0.695 | Fungi_unclassified | Fungi_unclassified | Fungi_unclassified | Fungi_unclassified |
| ASVF_12 | 1 | 2.707 | 0.703 | Sordariomycetes | Hypocreales | Nectriaceae | Gibberella |
| ASVF_59 | 1 | -0.76 | 0.693 | Leotiomycetes | Thelebolales | Pseudeurotiaceae | Pseudogymnoascus |
| ASVF_55 | 0 | 1.853 | 0.57 | Sordariomycetes | Hypocreales | Hypocreales | Hypocreales |
| ASVF_24 | 1 | -0.76 | 0.56 | Sordariomycetes | Trichosphaeriales | Trichosphaeriaceae | Nigrospora |
| ASVF_37 | 2 | 1.733 | 0.69 | Sordariomycetes | Glomerellales | Plectosphaerellaceae | Gibellulopsis |
| ASVF_85 | 0 | 0.732 | 0.711 | Sordariomycetes | Sordariales | Lasiosphaeriaceae | Lasiosphaeriaceae |
| ASVF_29 | 0 | 0.359 | 0.468 | Sordariomycetes | Glomerellales | Plectosphaerellaceae | Gibellulopsis |
| ASVF_126 | 0 | -0.014 | 0.444 | Sordariomycetes | Hypocreales | Hypocreales_fam_Incertae_sedis | Sarocladium |
| ASVF_133 | 0 | 1.853 | 0.675 | Sordariomycetes | Hypocreales | Hypocreales_fam_Incertae_sedis | Acremonium |
| ASVF_127 | 1 | 1.468 | 0.346 | Eurotiomycetes | Chaetothyriales | Herpotrichiellaceae | Exophiala |
| ASVF_73 | 2 | -0.119 | 0.719 | Tremellomycetes | Tremellales | Bulleribasidiaceae | Vishniacozyma |
| ASVF_97 | 1 | 0.973 | 0.623 | Sordariomycetes | Hypocreales | Nectriaceae | Fusarium |
| ASVF_15 | 0 | 0.359 | 0.726 | Sordariomycetes | Xylariales | Xylariales | Xylariales |
| ASVF_64 | 2 | 3.586 | 0.529 | Rhizophlyctidomycetes | Rhizophlyctidales | Rhizophlyctidaceae | Rhizophlyctis |
| ASVF_110 | 3 | -0.662 | 0.666 | Dothideomycetes | Pleosporales | Phaeosphaeriaceae | Phaeosphaeria |
| ASVF_11 | 3 | 0.082 | 0.648 | Sordariomycetes | Hypocreales | Nectriaceae | Gibberella |
